# The MarR-like protein PchR (YvmB) regulates expression of genes involved in pulcherriminic acid biosynthesis and in the initiation of sporulation in *Bacillus subtilis*

**DOI:** 10.1186/s12866-016-0807-3

**Published:** 2016-08-20

**Authors:** Paola Randazzo, Anne Aubert-Frambourg, Alain Guillot, Sandrine Auger

**Affiliations:** Micalis Institute, INRA, AgroParisTech, Université Paris-Saclay, 78350 Jouy-en-Josas, France

**Keywords:** Pulcherriminic acid, YvmB, MarR-type regulator, *B. subtilis*, Iron metabolism, Sporulation

## Abstract

**Background:**

Cyclodipeptides and their derivatives constitute a large class of peptide natural products with noteworthy biological activities. In some yeasts and bacterial species, pulcherriminic acid derived from cyclo-L-leucyl-L-leucyl is excreted and chelates free ferric ions to form the pulcherrimin. In *Bacillus subtilis*, the enzymes YvmC and CypX are known to be involved in pulcherriminic acid biosynthesis. However, the mechanisms controlling the transcription of the *yvmC*-*cypX* operon are still unknown.

**Results:**

In this work, we demonstrated that the *B. subtilis* YvmB MarR-like regulator is the major transcription factor controlling *yvmC*-*cypX* expression. A comprehensive quantitative proteomic analysis revealed a wide and prominent effect of *yvmB* deletion on proteins involved in cellular processes depending on iron availability. In addition, expression of *yvmB* depends on iron availability. Further analysis with real-time *in vivo* transcriptional profiling allowed us to define the YvmB regulon. We identified *yvmBA*, *yvmC-cypX* and *yvnB* for negative regulation and *yisI* for positive regulation. In combination with genetic approaches, gel mobility shift assays indicated that a 14-bp palindromic motif constitutes the YvmB binding site. It was unexpected that YvmB controls expression of *yisI*, whose encoding protein plays a negative role in the regulation of the sporulation initiation pathway. YvmB appears as an additional regulatory element into the cell’s decision to grow or sporulate.

**Conclusion:**

Our findings reveal a possible role of the *B. subtilis* YvmB regulator in the regulatory networks connected to iron metabolism and to the control of proper timing of sporulation. YvmB was renamed as PchR controlling the pulcherriminic acid biosynthetic pathway of *B. subtilis*.

**Electronic supplementary material:**

The online version of this article (doi:10.1186/s12866-016-0807-3) contains supplementary material, which is available to authorized users.

## Background

Cyclodipeptides and their derivatives constitute a large class of secondary metabolites that are mainly synthesized by microorganisms. The biological role of theses peptide natural products is mostly unknown but such compounds are of interest due to their potential antibacterial, antifungal, antitumoral or antiinflammatory activities. Cyclodipeptides from *Pseudomonas aeruginosa* exhibit cytotoxic properties towards human tumor cells [1], albonoursin produced by *Actinomyces tumemacerans* present antibacterial activities [2–4] whereas mycocyclosin may be essential for *Mycobacterium tuberculosis* viability [5, 6]. Some yeasts and bacterial *Bacillus* species synthesize pulcherriminic acid that is derived from cyclo-L-leucyl-L-leucyl (cLL) [7–10]. Pulcherriminic acid is secreted in the growth medium and chelates ferric ions by a non-enzymatic reaction to form an extracellular red pigment, the pulcherrimin. In *Metschnikowia pulcherrimina* strains, pulcherrimin appears to be involved in their antagonistic effects on the other microorganisms by depletion of iron in the growth medium [11–13]. In *Bacillus subtilis*, the enzymes involved in pulcherriminic acid synthesis were more recently identified. The cyclodipeptide synthase YvmC utilizes charged leucyl tRNAs as substrate to catalyze the formation of cLL [14, 15]. The gene downstream of *yvmC*, *cypX*, encodes a cytochrome P450, which is implicated in transformation of cLL into pulcherriminic acid [16].

The extended bacterial MarR family of transcriptional regulators is involved in the regulation of various cellular processes such as regulation of response to antibiotics, aromatic compounds and oxidative stress agents [17–22]. They can also play a crucial role in virulence factor production, enabling the pathogenic bacteria to adapt to host environments [23–26]. The majority of MarR family members have been characterized as transcriptional repressors although a few examples of transcriptional activators have also been reported [27–29]. They function as homodimers and bind inverted repeat nucleic sequence [30–32]. A genomic locus generally consists of divergently oriented genes encoding the MarR homolog and the gene(s) under its control. By binding to a specific site in the intergenic regions between divergently transcribed genes, the MarR homolog represses transcription of both [32]. Another defining feature of MarR homologs is their response to specific ligands. In absence of ligand, specific DNA binding occurs, most often resulting in repression of the transcription, while binding of the ligand results in attenuated DNA binding and hence de-repressed gene expression [31]. For many MarR-type homologues, as well as for many transcription factors, the natural ligand is still unknown.

The objective of this study was to identify the mechanisms of regulation of the pulcherriminic acid biosynthetic pathway in the soil bacterium *B. subtilis*. We demonstrated that the uncharacterized *B. subtilis* YvmB MarR-type regulator is the major regulator controlling expression of the *yvmCcypX* operon. In combination with genetic approaches, gel mobility shift assays allowed us to define a palindromic motif that constitutes the YvmB-binding site. We also characterized the YvmB regulon, which is composed of four transcriptional units. In addition, a comprehensive quantitative proteomic analysis showed significant repercussions of *yvmB* deletion for proteins associated to various cellular processes such as metabolism, translation, stress response and biosynthesis of cell wall.

## Results

### Identification of YvmB as repressor of *yvmC*-*cypX* and *yvmBA*

Pairwise alignment of the amino acid sequence of YvmB with proteins from databases, reveals similarity in the secondary structures between YvmB and other characterized MarR regulators (Additional file 1: Figure S1).

**Fig. 1:**
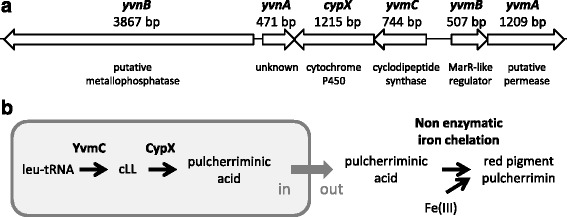
Involvement of the *yvmC-cypX* operon in pulcherriminic acid biosynthesis. **a** Genetic organisation of the *yvnB*, *yvnA*, *yvmC-cypX* and *yvmBA* genes in the *B. subtilis* genome. The function of the corresponding encoded proteins is indicated. **b** Scheme of the pulcherriminic acid biosynthetic pathway in *B. subtilis*. The cyclodipeptide synthase YvmC utilizes charged leucyl tRNAs as substrate to catalyze the formation of cyclo-L-leucyl-L-leucyl (cLL). The cytochrome P450 CypX is implicated in the transformation of cLL into pulcherriminic acid. Pulcherriminic acid is excreted from the cells by an unknown mechanism and chelates free Fe(III) by a non-enzymatic reaction to form an extracellular red pigment, the pulcherrimin

**Fig. 2:**
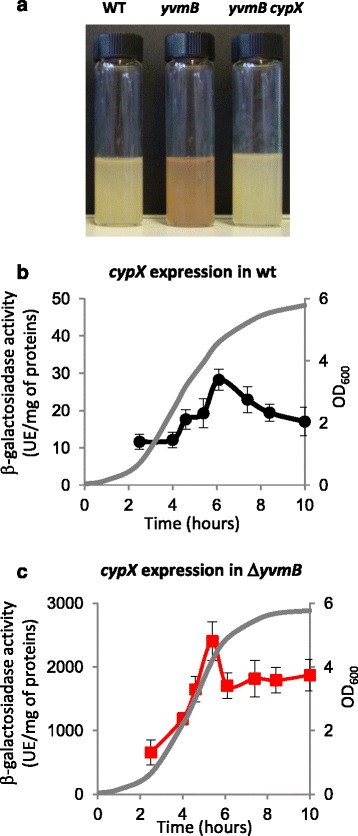
Involvement of YvmB as repressor in the control of *yvmC-cypX* expression. **a** The strains were cultivated in MS medium containing 100 μM FeCl_3_ for 16 h. at 37 °C in flasks with continuous agitation at 200 rpm. BSB1 wild-type strain; BSAS82 Δ*yvmB*::*aphA3*; BSAS147 Δ*yvmB*::*aphA3 cypX*::*lacZ*. Cultures were transferred in tubes for the pictures. **b** and **c** Expression of a *cypX*::*lacZ* transcriptional fusion was compared in wild-type and Δ*yvmB* cells. Strains were grown in LB medium. Growth was monitored by measuring OD_600_: grey curves. β-galactosidase activity of the *cypX*::*lacZ* fusion is indicated: *black circles*, in the wild-type (strain BFA815); *red squares*, in Δ*yvmB* cells (strain BSAS147). Values are the means from at least four independent experiments

The *yvmB* gene is divergently transcribed from the *yvmC*-*cypX* operon (Fig. 1) [33]. We observed that cells carrying a *yvmB* deletion overproduced a red pigment after entry into the stationary growth phase, in the iron-rich MS medium (Fig. 2a). To test whether YvmB controls *yvmC*-*cypX* expression, a transcriptional fusion between *cypX* and the *lacZ* reporter gene was constructed and studied in the wild-type and Δ*yvmB* cells (Table 1). In the wild-type, measurement of β-galactosidase activity revealed a slight increased of *cypX* expression during entry into stationary phase (Fig. 2b). In the Δ*yvmB* deletion mutant, *cypX*::*lacZ* was 100-fold up-regulated (Fig. 2c), suggesting that YvmB is the transcriptional repressor of *yvmC*-*cypX* operon expression. In addition, inactivation of the *cypX* gene in Δ*yvmB* cells abolished red pigment production confirming that the observed red color was probably due to the presence of pulcherrimin (Fig. 2a).

**Table 1:** *Bacillus subtilis* strains used in this study

Strain	Genotype^a^	Source
BSB1	*trp* ^*+*^	(Nicolas et al. 2012 [33])
BFS815^b^	*trpC2 cypX’*::*lacZ erm*	Laboratory stock
BSAS82	Δ*yvmB*::*aphA3*	This work
BSAS108	*amyE*::p*yvmB’*-*lacZ cat*	This work
BSAS109	*amyE*::p*yvmB’*-*lacZ cat* Δ*yvmB*::*aphA3*	This work
BSAS153	*amyE*::p*yvmB’*-*lacZ cat* Δ*ccpA*::*spc*	This work
BSAS122	*amyE*::pA*yvmC*’-*lacZ cat*	This work
BSAS143	*amyE*::pA*yvmC’*-*lacZ cat* Δ*yvmB*::*aphA3*	This work
BSAS147	*trpC2 cypX’*::*lacZ erm* Δ*yvmB*::*aphA3*	This work
BSAS155	*amyE*::pA*yvmC’-lacZ cat Δ*yvmB*::*aphA3*	This work
BSAS156	*amyE*::pA*yvmC’-lacZ cat	This work
BSAS185	*amyE*::pC*yvmC’-lacZ cat*	This work
BSAS189	*amyE*::pB*yvmC’-lacZ cat*	This work
BSAS195	*amyE*::pC*yvmC’-lacZ cat* Δ*yvmB*::*aphA3*	This work
BSAS199	*amyE*::pB*yvmC’-lacZ cat* Δ*yvmB*::*aphA3*	This work
BLUC135	*amyE*::pA*yvmB*’-*luc cat*	This work
BLUC136	*amyE*::pB*yvmB*’-*luc cat*	This work
BLUC137	*amyE*::pC*yvmB*’-*luc cat*	This work
BLUC138	*amyE*::pE*yvmB*’-*luc cat*	This work
BLUC139	*amyE*::pD*yvmB*’-*luc cat*	This work
BLUC169	*amyE*::pF*yvmB*’-*luc cat*	This work
BSPR276	*yvnB*’-*luc cat*	This work
BSPR289	*yvnB*’-*luc cat* Δ*yvmB*::*aphA3*	This work
BSPR452	*yisI*’-*luc cat*	This work
BSPR453	*yisI*’-*luc cat* Δ*yvmB*::*aphA3*	This work

^a^ *cat* pC194 chloramphenicol acetyl-transferase gene, *aphA3 Enterococcus faecalis* kanamycin-resistance gene, *erm* erythromycin-resistance gene, *spc Staphylococcus aureus* spectinomycin-resistance gene

*Strain constructed as part of the EC project for the functional characterization of the *B. subtilis* genome

**Fig. 3:**
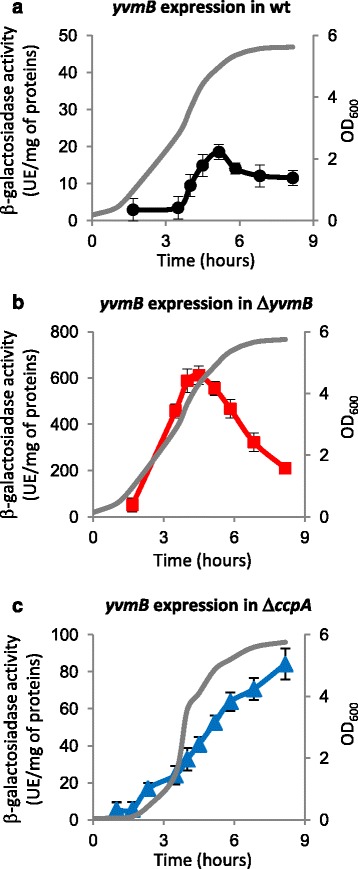
Involvement of YvmB CcpA as repressors in the control of *yvmB* expression. **a**, **b** and **c**) Expression of a p*yvmB’-lacZ* transcriptional fusion was compared in wild-type (strain BSAS108), Δ*yvmB* cells (strain BSAS109) and Δ*ccpA* cells (strain BSAS153). Strains were grown in LB medium. Growth was monitored by measuring OD_600_: grey curves. β-galactosidase activity of the p*yvmB’-lacZ* fusion is indicated: black circles, in the wild-type; red squares, in Δ*yvmB* cells; blue triangles, in Δ*ccpA* cells. Values are the means from at least four independent experiments

The *yvmB* gene is coexpressed with *yvmA* leading to a *yvmBA* transcript [33]. To ask whether *yvmB* might be subject to autoregulation like other MarR-type repressors, transcription analyses were performed. A transcriptional fusion between the *yvmB* promoter region (from nucleotide −291 to +9 relative to the translational start site) and the *lacZ* reporter gene was constructed and integrated into the *amyE* locus of the wild-type and Δ*yvmB* cells. Analysis of the β-galactosidase activity revealed that expression of *yvmB* increased during entry into stationary phase (Fig. 3a). The 30-fold up-regulation of *yvmB*-promoter-*lacZ* acitivity in Δ*yvmB* cells indicated that *yvmB* is subject to autorepression (Fig. 3b).

### Expression of *yvmB* is repressed by CcpA in stationary growth phase

Expression of *yvmB* was proposed to be subject to repression by glucose by direct binding of the carbon metabolism regulator CcpA to a *cre* site, which overlaps the −35 region [34]. Therefore, we investigated expression of the *yvmB*-promoter-*lacZ* fusion in a *ccpA* mutant. Expression of *yvmB* was up-regulated in Δ*ccpA* cells upon the stationary growth phase (Fig. 3c). This result confirmed that CcpA acts as negative regulator of *yvmB* expression. Therefore, *yvmB* expression undergoes a dual regulation by YvmB and CcpA depending on growth phase.

### Transcription of *yvmB* is induced by iron starvation

Pulcherriminic acid is known to be secreted in the growth medium and to chelate ferric ions. Here, we examined the effect of iron starvation on *cypX*::*lacZ* expression. The BFS815 strain (Table 1) was cultivated in LB medium and a sample was spread onto solid medium containing X-gal. A drop of 10 mM bipyridyl, a specific iron chelator, was deposited at the center of the plate. After incubation, a white ring was observed around the bipyridyl drop (Additional file 2: Figure S2) which indicates that iron starvation does not induce *yvmC-cypX* expression. We further tested the effect of iron starvation on the *yvmB*-promoter-*lacZ* fusion (strain BSAS108) using the same experimental procedure. Remarkably, a characteristic blue ring was observed around the bipyridyl drop (Additional file 2: Figure S2) indicating that iron deficiency induces *yvmB* expression.

### Effect of *yvmB* deletion on *B. subtilis* proteome

To understand the physiological state of cells overproducing pulcherriminic acid, we performed a comparative analysis of the cytosolic and membrane proteome of the wild-type and Δ*yvmB* cells. The optimized analyses of four technical replicates resolved more than 1700 proteins (Additional file 3: Table S1). Only the statistically significant (*p* < 0.05; Kruskal-Wallis test and one way ANOVA) differentially expressed proteins were considered (Additional file 4: Table S2). Among the 32 differentially abundant proteins (17 up-regulated and 15 down-regulated), 4 were common between the membrane and the cytosolic fractions. The results are summarized in the Table 2.

**Table 2:** Proteins up- or down-regulated in a *B. subtilis yvmB* mutant

Protein	Δ*yvmB* vs wt^a^	Protein description
Carbon metabolism and transport		
GlpK	24,50	Glycerol kinase
Tkt	16,00	Transketolase
Pyk	6,25	Pyruvate kinase
CitB	−13,75	Aconitate hydratase
CitZ*	−7,25	Citrate synthase 2
PtsH	−7,25	Phosphocarrier protein HPr of the PTS
Pgm	−5,75	phosphoglycerate mutase
Iron metabolism/electron transport/respiration		
CypX*	24,25	Cytochrome P450, cLL dipeptide oxidase
Ndh	19,50	NADH dehydrogenase
SufD	6,25	FeS cluster assembly protein
YumC	6,00	ferredoxin/flavodoxin reductase
YjlC	−6,00	Putative NADH dehydrogenase
Amino acid/nitrogen/nucleotides metabolism		
PyrG	12,25	CTP synthase (NH3, glutamine)
MtnK	11,25	Methylthioribose kinase
RocD	8,00	Ornithine aminotransferase
PupG	−8,00	Purine nucleoside phosphorylase
OppA	−6,75	Oligopeptide-binding protein
Cell wall synthesis/lipid metabolism		
MurAA	8,25	UDP-N-acetylglucosamine 1-carboxyvinyltransferase
YkuQ	8,25	Similar to tetrahydrodipicolinate succinylase
PgcA	7,50	Phosphoglucomutase
FabI	−18,50	Enoyl-[acyl-carrier-protein] reductase [NADH]
Translation		
RplL*	−41,00	50S ribosomal protein L7/L12
RpsH	−18,00	30S ribosomal protein S8
ProS	−9,25	Proline-tRNA ligase
ArgS	−7,50	Arginyl-tRNA synthetase
Transcription factor and their control		
SalA	5,75	MRP family regulator
Stress response		
CspD	11,25	Cold-shock protein, molecular chaperone
KatA	8,75	Vegetative catalase
BrxB	−6,00	Bacilliredoxin
Other functions		
YvmA	13,00	Putative permease
YkkA	−9,50	putative Pit accessory protein
Hbs*	−9,50	DNA-binding protein HU 1

^a^Experiment was performed in four replicates

*Proteins with an asterisk were identified both in the cytosolic and membrane fractions

Proteomic analysis of a *yvmB* mutant confirmed a 24- and 13-fold enhanced production of the YvmB primary targets, CypX and YvmA. It also revealed a wide and prominent effect on proteins associated with carbon metabolism, translation, stress response and biosynthesis of cell wall. Remarkably, several of these proteins have a function related to metal ions metabolism. Shifts in metabolic pathways particularly occur in response to iron availability [35, 36]. CitB and CitZ are down-regulated in a *yvmB* mutant. Both proteins are known to be less synthesized under conditions of iron limitation [37–39]. Moreover, the down-regulated ribosomal proteins RplL and RpsH belong to the stringent response, which could be initiated by iron limitation [40]. Iron participates in many metabolic processes related to electron transfer and redox state of the cell, as well as to pH homeostasis [36, 41]. Interestingly, proteins involved in FeS cluster assembly and in respiration, like SufD and Ndh, respectively, are more abundant in a *yvmB* mutant. In the same line, a *yvmB* mutant shows increased level of the ferredoxin-dependent reductase YumC, which is involved in electron transport. The up-regulated protein KatA is part of the iron-dependent peroxide stress response mediated by PerR, which is activated when aerobically growing cells enter the stationary phase [42]. Most of the PerR regulon was shown to be repressed when cells are exposed to high levels of iron [36]. Up-regulated MurAA, YkuQ and PgcA are related to cell wall synthesis and lipid biosynthesis. Membrane structure remodeling can result from interaction of ferric ion with external membrane phospholipids bilayer [43–45]. Therefore, deletion of *yvmB* induces disruptions of proteins involved in cellular processes that depend on iron availability.

### Determination of the YvmB recognition site in the *yvmC* promoter

**Fig. 4:**
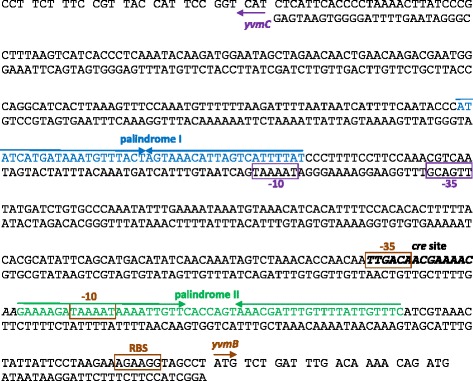
Promoter region of the *yvmC*-*yvmB* divergent genes. The −10 and −35 regions of *yvmC* (in *purple*) and the −10 and −35 regions of *yvmB* (in *brown*) are indicated according to Nicolas et al. [33]. The potential RBS (Ribosomal Binding Site) of the *yvmB* gene is indicated. The bold letters correspond to the *cre* site. The *blue arrows* and the *blue letters* indicate the palindromic sequence I. The *green arrows* and the *green letters* indicate the palindromic sequence II

**Fig. 5:**
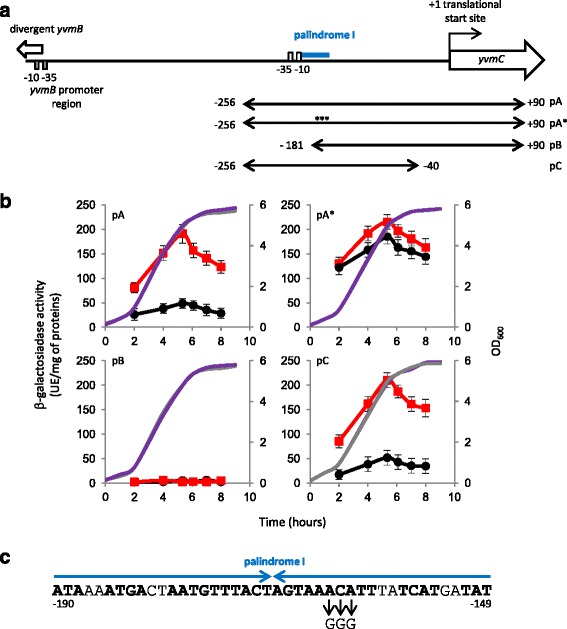
Expression of various *yvmC* promoter *lacZ* fusions in wild-type and Δ*yvmB* strains. **a** Structure of the *yvmC* operator region. The −10 and −35 regions similar to the consensus sequence of σ^A^-dependent promoters are indicated: white boxes. Location of the palindromic sequence, palindrome I, is indicated in blue. The DNA fragments used to construct p*yvmC’*-*lacZ* transcriptional fusions are diagrammed. They are numbered with respect to the translational start site of *yvmC*. Stars indicated the location of three point mutations introduced in the pA*yvmC’-*lacZ* fusion. The −10 and −35 regions located in the *yvmB* promoter region are indicated: grey boxes. **b** Cells were grown in LB medium. Growth was monitored by measuring OD_600_: grey line, wild-type; purple line, Δ*yvmB*. Expression of the four p*yvmC’-lacZ* transcriptional fusions was measured: black circles, in the wild-type; red squares, in Δ*yvmB* cells. Values are the means from at least three independent experiments. **c** Nucleotide sequence of the palindromic motif (*blue arrows*), palindrome I. The nucleotides in bold belong to the inverted repeat nucleic sequence. The A, C and A nucleotides at position −162, −163 and −164 (relative to the *yvmC* translational start site) were replaced by three nucleotides G to construct the pA*yvmC’-*lacZ* fusion

In order to identify genes directly regulated by YvmB, we first attempted to identify the YvmB DNA-binding site. The DNA sequence of the *yvmC* promoter region is presented in Fig. 4. The −10 (TAAAAT) and −35 (TTGACG) σ^A^-dependent regions are indicated according to the genome reannotation data [33]. To identify the DNA regions involved in *yvmC* regulation, four different parts of the promoter region of the *yvmC* gene were each fused with the *lacZ* reporter gene and integrated at the *amyE* locus of the wild-type strain (Fig. 5a) (Table 1). β-galactosidase activities were measured during growth in LB medium (Fig. 5b). The pA*yvmC* and pC*yvmC* fusions were 4-fold more expressed in Δ*yvmB* cells than in the wild-type. The absence of pB*yvmC* expression was consistent with the position of the −10 and −35 σ^A^-dependent regions. Remarkably, a 42-bp palindromic sequence lies between nucleotides −189 to −149 relative to the *yvmC* translational start site (Fig. 5c) and was called palindrome I. Three point mutations were introduced in this palindromic sequence. The A, C and A nucleotides at position −162, −163 and −164 were replaced by three nucleotides G (Fig. 5c). The resulting pA* fusion was constitutively expressed in the wild-type strain (Fig. 5b), indicating that palindrome I is the major *cis*-regulatory sequence involved in *yvmC* repression by YvmB.

### Detection of several regions involved in the control of *yvmB* by YvmB

**Fig. 6:**
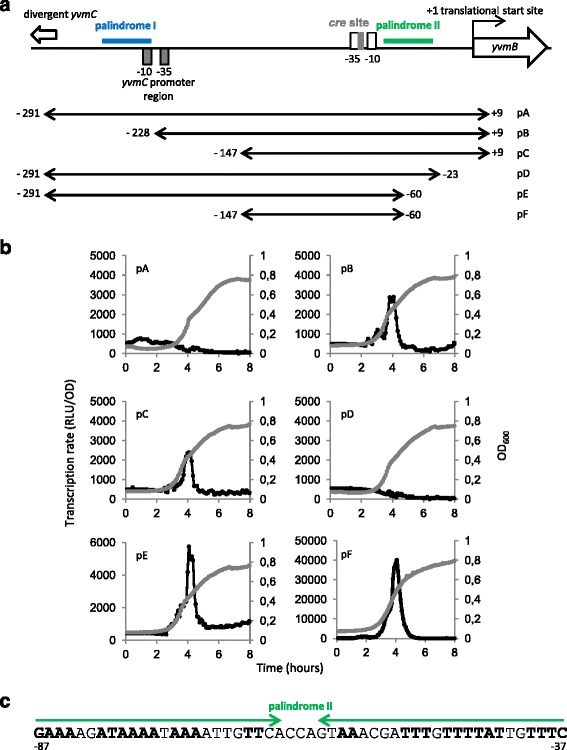
Expression of various *yvmB* promoter *luc* fusions. **a** Structure of the *yvmB* operator region. The −10 and −35 regions similar to the consensus sequence of σ^A^-dependent promoters are indicated: white boxes. The location of the *cre* site (Blencke et al. [34]) is indicated: grey box. Location of the palindromic sequence, palindrome I, is indicated in blue. Location of the palindromic sequence, palindrome II, is indicated in green. The different fusions with the *luc* gene are numbered with respect to the translational start site of *yvmB*. The −10 and −35 regions located in the *yvmC* promoter region are indicated: grey boxes. **b** Cells were grown in LB medium. Growth was monitored by measuring OD_600_: grey lines. Expression of the six p*yvmB’-luc* transcriptional fusions was measured in the wild-type: black lines. For each strain, one representative curve, out of three independent replicates realized, is shown. Note that the X-axe is different for the pF fusion to get a better view of the data. **c** Nucleotide sequence of the palindromic motif (*green arrows*), palindrome II. The nucleotides in bold belong to an inverted repeat nucleic sequence

The DNA sequence of the *yvmB* promoter region is shown in Fig. 4. The −10 (TAAAAT) and −35 (TTGACA) σ^A^-dependent regions are indicated [33]. To determine the regions involved in *yvmB* regulation, strains carrying transcriptional *luc* fusions using different promoter sub-regions of *yvmB* at the *amyE* locus were constructed (Fig. 6a) (Table 1). Expression was very low for the large pA*yvmB* fusion (Fig. 6b). The pB*yvmB* and pC*yvmB* fusions were 6- and 5-fold up-regulated during entry in the stationary phase indicating that the −291 to - 228 region was involved in *yvmB* repression. This corresponded to the location of palindrome I identified above, which lies from nucleotides −278 to −236 relative to the *yvmB* translational start site (Fig. 4). Thus, YvmB is involved in the control of its own transcription via palindrome I.

A second set of *luc* fusions was constructed. Expression of the pD*yvmB* fusion was not detected whereas the pE*yvmB* fusion was 10-fold up-regulated in entry to stationary phase (Fig. 6b). Therefore, the −60 to −23 region was also involved in *yvmB* repression. Remarkably, this region overlaps a 60-bp imperfect palindromic sequence that we named palindrome II (Fig. 6c). The short pF*yvmB* fusion, without palindrome I nor palindrome II, was fully depressed in entry to stationary phase (Fig. 6b) showing a cumulative repressive effect of palindromes I and II. Remarkably, the pF*yvmB* fusion yielded a very high and transient induction probably due to the repressive effect of CcpA in stationary phase, as shown above (Fig. 3c).

### Definition of a YvmB box consensus and prediction of YvmB boxes within the whole-genome sequence

**Fig. 7:**
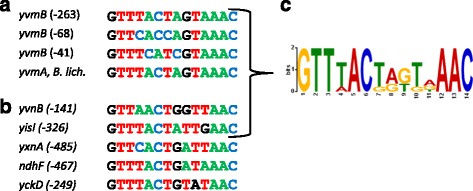
Identification of a 14 bp YvmB-box consensus sequence. **a** Alignment of potential 14 bp YvmB box motifs identified in the promoter region of the *B. subtilis yvmB* gene and in the promoter region of the *B. licheniformis yvmA* gene. Position of the boxes are indicated relative to the translational start sites. **b** Identification of additional putative YvmB boxes. Position of the boxes are indicated relative to the translational start sites: *yvnB*; similar to metallophosphatase; *yisI*, Spo0A-P phosphatase; *yxnA*, similar to glucose 1-dehydrogenase; *ndhF*, NADH dehydrogenase (subunit 5); *yckD*, unknown. **c** The 14-nt palindromic consensus sequence of the YvmB box motif. The size of the nucleotides at each position indicates its relative prevalence in sequences used as training set for MEME algorithm

Browsing the genome sequence of A-T-rich Firmicutes spp., we detected YvmA, YvmC and CypX-like proteins in the genome of *Bacillus licheniformis* ATCC 14580. A sequence similar to palindrome I is present upstream of *yvmA* (Additional file 5: Figure S3). Inspection of the promoter regions of *B. subtilis yvmB* and *B. licheniformis yvmA* genes revealed conservation of a 14-bp core region containing an inverted repeat (Fig. 7a). We showed above that mutation of the conserved nucleotides in position 13 and 14 resulted in full up-regulation of *yvmC* expression (Fig. 5). This motif could be viewed as the YvmB binding site (hereafter called the YvmB box). Indeed, three YvmB boxes were identified in the *yvmB* promoter region: (i) one in palindrome I; (ii) one in palindrome II; (iii) the third one, 41 bp upstream of the *yvmB* translational start site (Fig. 7a). This allowed us to propose a first YvmB box consensus sequence, GTTYMMYMGTAAAC.

We used this YvmB box consensus to search putative YvmB boxes within the *B. subtilis* whole-genome sequence, less than 500 bp upstream of a translational start and in a define promoter region according to Nicolas et al. [33]. This allowed the identification of 5 highly similar motifs preceding the *yckD*, *yisI*, *yvnB*, *yxnA* and *ndhF* genes (Fig. 7b). The corresponding proteins were not detected in our proteomic analysis. However, we assumed that YvmB could be involved in the transcriptional regulation of these 5 genes. By contrast, no evident YvmB box-like motif was detected upstream of the transcriptional units encoding proteins differentially abundant in the *yvmB* mutant suggesting an indirect effect of *yvmB* deletion.

### Interaction of YvmB with the *yvmB*, *yvnB* and *yisI* promoter regions

His-tagged YvmB protein was overproduced in *E. coli* and purified in a single step by Ni-chelate affinity chromatography to greater than 95 % homogeneity, as revealed by SDS-PAGE (Additional file 6: Figure S4). A sample of the purified protein was fractionated by chromatography on a gel-filtration column. The YvmB^His-tag^ protein eluted as a major peak with an apparent molecular mass of 45 ± 5 kDa (Additional file 6: Figure S4). As the YvmB^His-tag^ polypeptide has a molecular mass of 19.2 kDa, this means that the native protein is most likely a dimer.

**Fig. 8:**
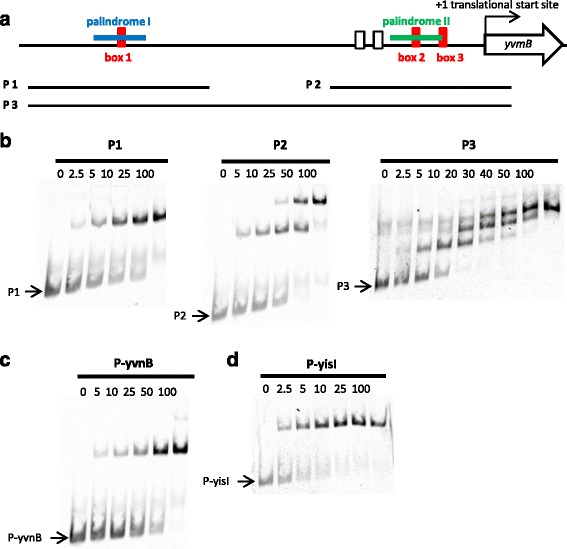
YvmB binding to the *yvmB-yvmC* intergenic region and to the *yvnB* and *yisI* promoter regions. **a** Location of the three identified YvmB boxes in the promoter region of *yvmB*. The −10 and −35 regions similar to the consensus sequence of σ^A^-dependent promoters are indicated: white boxes. Location of the palindromic sequence, palindrome I, is indicated in blue. Location of the palindromic sequence, palindrome II, is indicated in green. Location of the three YvmB boxes is indicated in red. The DNA fragments P1, P2 and P3 used to perform gel shift assays are diagrammed. The DNA probes used were: (**b**) probe P1, probe P2 and probe P3; (**c**) probe P-yvnB and (**d**) prove P-yisI. Increasing concentrations of purified YvmB^His-tag^ were used (nM)

Ability of YvmB^His-tag^ to bind to the *yvmB*-*yvmC* intergenic region was tested by gel retardation assays using three different DNA fragments as probes (Additional file 7: Table S3). The P1 fragment contains palindrome I, the P2 fragment contains palindrome II while the large P3 fragment covers the entire DNA region located between the *yvmB* and *yvmC* genes (Fig. 8a). One YvmB-DNA complex, with an apparent dissociation constant of 5 nM, was detected with P1 probe (Fig. 8b) in agreement with prediction of one YvmB recognition motif in this region. Two YvmB-DNA complexes were detected with P2 probe (Fig. 8b) in agreement with detection of two YvmB box motifs. Finally, gel retardation assays with P3 probe revealed three YvmB-DNA complexes, confirming the presence of three YvmB binding sites (Fig. 8b).

Gel shift assays were also performed with a DNA fragment corresponding to the *yvnB, yisI*, *yxnA*, *ndhF*, *yckD* promoter regions (Additional file 7: Table S3). Fragments (110 bp long) carrying putative YvmB boxes in the centre were subjected to gel retardation analysis with YvmB^His-tag^. No interaction was detected with probes corresponding to *yxnA*, *ndhF* and *yckD* promoter regions (data not shown). Binding of YvmB^His-tag^ to the P-yvnB and P-yisI probes resulted in shifts, for which we determined the apparent dissociation constant at 25 nM and 2.5 nM, respectively (Fig. 8c and d). It should be noted that a minor second protein-DNA complex formed in the presence of 100 nM YvmB^His-tag^ and probe P-yvnB. This could be due to the presence of a degenerated YvmB motif (GTTTACTGGGTAAT) located 119 bp upstream of *yvnB*. To conclude, the predicted YvmB box upstream of *yvnB* and *yisI* appears functional for YvmB binding *in vitro*. We assumed that YvmB could be involved in the transcriptional regulation of *yisI* and *yvnB.*

### Dual role of YvmB as repressor of *yvnB* and as activator of *yisI*

**Fig. 9:**
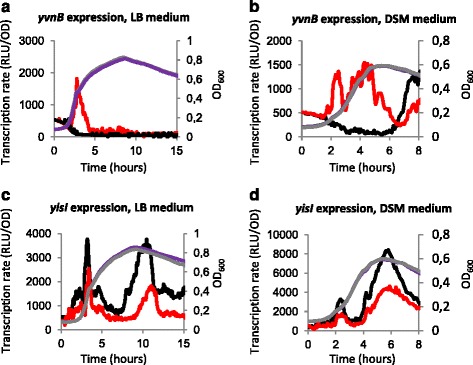
Involvement of YvmB in the control of *yvnB* and *yisI* expression. Growth was monitored by measuring OD_600_ every 5 min: grey line, wild-type; purple line, Δ*yvmB*. **a** Promoter activity (RLU/OD) of the transcriptional fusion *yvnB*’-*luc* in LB medium is indicated: black line, wild-type; red line, Δ*yvmB.* **b** Promoter activity (RLU/OD) of the transcriptional fusion *yvnB*’-*luc* in DSM sprorulation medium is indicated: black line, wild-type; red line, Δ*yvmB.* **c** Promoter activity (RLU/OD) of the transcriptional fusion *yisI*’-*luc* in LB medium is indicated: black line, wild-type; red line, Δ*yvmB.* **d** Promoter activity (RLU/OD) of the transcriptional fusion *yisI*’-*luc* in DSM sporulation medium is indicated: black line, wild-type; red line, Δ*yvmB.* One representative curve, out of three independent replicates realized, is shown

The DNA sequence of the *yvnB* and *yisI* promoter regions is shown in Additional file 8: Figure S5. The *yvnB* gene is located less than 1 kb upstream of the *yvmCcypX* operon (Fig. 1). We then tested the correlation between the presence of a YvmB box and YvmB dependent expression. For this purpose, we constructed a transcriptional fusion between the promoter region of *yvnB* and the luciferase gene in wild-type and Δ*yvmB* cells. Accordingly to Nicolas et al. [33], *yvnB* gene is highly expressed during sporulation condition. Luciferase activity was recorded during growth in standard LB medium and sporulation medium. Expression of the p*yvnB* fusion showed 10-fold up-regulation in Δ*yvmB* cells in LB and DSM media (Fig. 9a and b). This result validated the YvmB-dependent repression of the *yvnB* gene.

We also tested the activity of YvmB as transcriptional regulator of the *yisI* gene, which encodes a protein involved in the sporulation process. We constructed a transcriptional fusion between the promoter region of *yisI* and the luciferase gene in wild-type and Δ*yvmB* cells. Expression of the *yisI* fusion is 2-fold down-regulated in Δ*yvmB* cells in the stationary growth phase in LB and DSM media (Fig. 9c and d). Thus, YvmB positively regulates *yisI* expression.

Subsequently, we used the MEME standard bioinformatic method [46] to redefine the YvmB box consensus from the YvmB box sequences upstream of the *yvmB*, *yvmC*, *yvnB* and *yisI* promoter regions. The resulting new YvmB consensus sequence exhibited an inverted repeat sequence with highly conserved nucleotides at the external positions 1, 2, 3, 12, 13 and 14 and in the mid-positions 5 and 6 (Fig. 7c).

## Discussion

In this study, we identified the *B. subtilis* YvmB uncharacterized MarR-like protein as the main negative regulator of the pulcherriminic acid biosynthetic pathway. To our knowledge, this is the first report of the molecular and genetic mechanisms involved in the regulation of this pathway in microorganisms. Our genetic analysis, gel-shift assays and computational analysis highlight a 14-bp core perfect inverted repeat, which likely defines the YvmB binding site in the *yvmC*-*cypX* operator region. This motif overlaps the −10 promoter element of the *yvmC* target gene, as previously described for MarR-type regulators DNA-binding sites [32]. In addition, our work led to the identification of the YvmB regulon, which extends to *yvmBA* (divergent from *yvmC)*, *yvnB* and *yisI* genes. A sequence analysis of the YvmB boxes using the MEME suite produced a consensus composed of highly conserved nucleotides at the external positions 1, 2, 3, 12, 13 and 14 and at the mid-positions 5 and 6 (Fig. 7c). As these nucleotides are also present in the unbound YvmB boxes preceding the *yckD*, *yxnA* and *ndhF* genes, the local sequence environment might also be important for the specific DNA binding of YvmB.

The regulation of *yvmB* expression is complex since the activity of its promoter is modulated by at least two transcriptional regulators, CcpA and YvmB. CcpA seems to act as repressor of *yvmB* expression in the stationary growth phase while YvmB represses its own expression. Autoregulation is a characteristic of the MarR-like repressor [32]. We showed that YvmB binds specifically to three regions containing a YvmB box. The three YvmB boxes play a cumulative effect in *yvmB* repression. In addition, preliminary analysis of the purified YvmB^His-tag^ protein by analytical chromatography suggests that YvmB is a dimer in the absence of the effector. The nature of the metabolite controlling YvmB repressor activity by binding to DNA remains to be clarified. Remarkably, expression of *yvmB* is also up-regulated in response to iron starvation. Our results support the existence of an auxiliary transcriptional mechanism, independent of YvmB, to control *yvmB* expression in response to iron availability.

An unexpected finding is the activation of the *yisI* gene by YvmB during entry into the stationary growth phase. To our knowledge there are few examples of MarR-like regulator showing a dual role as a repressor and an activator [29]. Remarkably, the YisI protein is an aspartyl-phosphate phosphatase, which specifically dephosphorylates the sporulation transcription factor Spo0A-P and negatively regulates the sporulation initiation pathway in order to control the proper timing of sporulation [47]. It is known that the *yisI* gene is expressed mainly during the transition phase between exponential and stationary phase, its induction is dependent upon the presence of Spo0A [47]. By our proteomic analysis, two proteins of the Spo0A regulon are induced in a *yvmB* mutant. These are represented by the RocD ornithine aminotransferase and the Tkt transketolase, which might be due to a difference in phosphorylated/unphosphorylated form of the Spo0A regulator in a *yvmB* mutant. Therefore, YvmB appears an additional regulatory element into the cell’s decision to grow or sporulate. A database search revealed that *yvmBA*, *yvmC*-*cypX* and *yisI* genes are present in *B. licheniformis* and *Bacillus thuringiensis*, suggesting the functional conservation of the role of YvmB in pulcherriminic acid synthesis and in sporulation in other Bacilli.

The exact role of pulcherriminic acid produced by *B. subtilis* is still unclear. Expression of *yvmC*-*cypX* is not up-regulated in response to iron deficiency in agreement with Kupfer et al., who showed that similar amounts of pulcherriminic acid were detected in the presence of both low and high concentrations of ferric ions [48]. Since pulcherrimin is a large nondiffusible complex [8, 9, 49], mechanism of iron depletion is different from the mechanisms operating in microbes that release siderophores into the environment for iron acquisition. Our comprehensive quantitative proteomic analysis revealed that *yvmB* deletion induces changes of proteins involved in cellular processes depending on iron availability. However, we did not identify evident YvmB-box motifs upstream of the transcriptional units encoding these proteins. We assume that chelation of external iron by pulcherriminic acid could disrupt iron-dependent processes that can explain the wide indirect cellular changes into Δ*yvmB* cells.

Here, we demonstrated that YvmB controls expression of the *yvmA* and *yvnB* genes, which encodes a putative permease and a putative metallophosphatase, respectively. In *M. pulcherrimina*, pulcherrimin is involved in antimicrobial effects by depletion of iron in the growth medium [11–13]. *M. pulcherrimina* also secretes lytic enzymes such as chitinase that may contribute to the overall antagonistic effects of related *M. pulcherrimina* strains [50]. The role of YvmA and YvnB in potential antagonist effects of pulcherrimin produced by *B. subtilis* deserve further studies.

## Conclusions

This study allowed to identify the mechanism of regulation of the genes involved in the biosynthesis of pulcherriminic acid in *B. subtilis*. We identified the uncharacterized YvmB MarR-like protein as the main regulator of the *yvmCcypX* operon. Additionally, we defined the YvmB regulon comprising at least four transcriptional units. Our results also highlight a complex impact of YvmB on cellular processes depending on iron availability and on the regulation of the initiation of sporulation. Further studies will be required to investigate the role of the genes controlled by YvmB.

## Methods

### Bacterial strains and growth conditions

The *B. subtilis* strains used in this work are listed in Table 1. *B. subtilis* strains are derivatives of the BaSysBio reference strain BSB1 which is a tryptophan-prototrophic (*trp*+) 168 strain [33]. *Escherichia coli* strains used were TG1 (Lab strain) for plasmid construction and ER2566 (New England Biolabs) for protein expression and purification. *B. subtilis* cells were grown in Luria-Bertani (LB) medium or in MS medium containing 62 mM K_2_HPO_4_, 44 mM KH_2_PO_4_, 17 mM trisodium citrate, 11 mM K_2_SO_4_, 0.4 % glucose, 0.06 % L-glutamine, 0.01 % L-tryptophane, 0.1 % casamino acids, 2 mM MgSO_4_, 1 mM CaCl_2_, 100 μM FeCl_3_ citrate, 112 μM ZnCl_2_; 5 μM MnCl_2_; 2.5 μM CuCl_2_. *E. coli* cells were propagated in LB medium. Antibiotics were added at the following concentrations when required: 100 μg ampicillin ml^−1^; 5 μg chloramphenicol ml^−1^; 100 μg spectinomycin ml^−1^; 5 μg kanamycin ml^−1^; 10 μg erythromycin ml^−1^. Solid media were prepared by addition of 20 g Agar noble l^−1^ (Difco). Standard procedures were used to transform *E. coli* [51] and *B. subtilis* [52].

The loss of amylase activity was detected as described by Stülke et al. [53]. β-galactosidase specific activity was measured as described by Miller [54] with cell extracts obtained by lysozyme treatment. One unit of β-galactosidase activity was defined as the amount of enzyme that produces 1 nmol *o*-nitrophenol min^−1^ at 28 °C. The mean values of three independent experiments are presented. Standard deviations were less than 20 % of the mean.

### DNA manipulations

DNA manipulations and cloning procedures were performed as described elsewhere [51]. Restriction enzymes, *Pfu* DNA polymerase and phage T4 DNA ligase were used as recommended by the manufacturer (Biolabs). DNA fragments were purified from agarose gels using the QIAquick kit (Qiagen).

### Construction of plasmids and strains

The BSAS82 *yvmB* mutant was constructed by homologous replacement of the YvmB coding sequence with a kanamycin resistance gene (*aphA3*) using a joining PCR technique [55]. The *aphA3* gene was first amplified. The region upstream of the *yvmB* gene (nucleotide −990 to +52 relative to the translational start site) was amplified by PCR with a 24 bp *aphA3* fragment at its 3′ end. The region downstream of *yvmB* (nucleotides +421 to +1495) was amplified with a 24 bp *aphA3* fragment at its 5′ end. The three DNA fragments were combined and then a PCR reaction was performed with the two external oligonucleotides. The final product, corresponding to the two regions flanking *yvmB* with the inserted *aphA3* cassette in between, was purified from a gel and used to transform *B. subtilis*. Integration and deletion were confirmed by PCR and verified by DNA sequencing.

The inactivation of the *cypX* locus while creating a *lacZ* fusion (strain BFS815) was done within the framework of a European project on the functional analysis of the genome of *B. subtilis* (see http://genome.jouy.inra.fr/cgi-bin/micado/index.cgi).

Plasmid pAC6 [53] allowed the construction of a transcriptional fusion between the promoter region of *yvmB* (nucleotides −291 to +9 relative to the translational start site) and the promoterless *lacZ* gene. The *yvmB* promoter region was amplified by PCR, with the creation of *Eco*RI and *Bam*HI sites. The PCR product was inserted into pAC6. The resulting plasmid was linearized with *Sca*I, which allowed the insertion of the transcriptional *lacZ* fusion as a single copy at the *amyE* locus (Table 1). The same procedure was used to construct a series of transcriptional fusions between the promoter region of *yvmC* and the *lacZ* gene.

To construct transcriptional fusions between the promoter region of *yvmB*, *yvnB* and *yisI* and the *luc* reporter gene, we used the assembly Gibson’s procedure [56] as previously described [57]. The PUC18cm-luc plasmid was used as template to amplify the *luc* reporter gene. The sequences of the resulting constructs were verified by DNA sequencing.

### Luciferase assay

For the detection of luciferase activity, strains were first grown in LB medium to an optical density at 600 nm (OD_600_) of 2. Cells were then centrifuged and resuspended in fresh minimal medium, adjusting all the cultures to an OD_600_ of 2. These pre-cultures were then diluted 20 fold in fresh minimal medium and 200 μl was distributed in each of two wells in a 96-well black plate (Corning). 10 μl of luciferin were added to each well to reach a final concentration of 1.5 mg/ml (4.7 mM). The cultures were incubated at 37 °C with agitation in a PerkinElmer Envision 2104 Multilabel Reader equipped with an enhanced sensitivity photomultiplier for luminometry. The temperature of the clear plastic lid was maintained at 38 °C to avoid condensation. Relative Luminescence Unit (RLU) and OD_600_ were measured at 5 min intervals.

### Protein extraction

Four independent cultures of each *B. subtilis* strains were grown in 500 mL of LB medium to an OD_600_ of 0.8. Cultures were harvested by centrifugation (6000 × g, 10 min, 4 °C) and washed twice with a cold low-salt buffer (150 mM NaCl, 10 mM Tris, 0.5 mM EDTA, pH 7.5). Resuspended bacteria were mechanically disrupted by a passage through a One Shot Cell Disrupter (Constant Systems Ltd., Warwickshire, UK) at 2.7 Kbar. Cell lysates were centrifuged (27 000 × g, 20 min, 4 °C) and resulting supernatants were treated to ultracentrifugation (100 000 × g, 1 h, 4 °C). Cytoplasmic fractions were considered as the soluble parts after a single ultracentrifugation step, while the remaining pellets were designated as crude membrane fraction. After resuspension in Bis-Tris NaCl buffer (50 mM bis-tris, 50 mM NaCl, pH7.2) followed by ultra-centrifugation (100 000 × g, 1 h, 4 °C), pellets were resuspended overnight in 150 μL resuspension buffer (20 mM Tris–HCl, 10 mM EDTA, pH7.5) and then added of 1 volume of SDS-PAGE sample buffer (125 mM Tris–HCl pH6.8, 20 % v/v glycerol, 10 % w/v SDS, 5 % β-mercaptoethanol). Protein concentration was determined by NanoDrop. All the samples were loaded on a 10 % NuPAGE Bis-Tris Gels (Invitrogen).

### Tryptic digestion in gel

All Coomassie-stained protein sample detected on SDS-PAGE were cut in small pieces and washed twice with 0.2 % TFA-50 % acetonitrile. The protein disulfide bridges were reduced by DTT 10 mM for one hour at 56 °C and resulting reduced cysteine were alkylated by iodoacetamide (50 mM) for 1 h at room temperature into darkness before the step of enzymatic digestion performed by adding 500 ng of sequencing grade modified trypsin (Promega) diluted in 25 mM NH_4_HCO_3_ for 18 h at 37 °C for each protein sample. Tryptic peptides were recovered by washing the gel pieces twice in 0.2 % TFA-50 % acetonitrile and once in 100 % acetonitrile and the supernatant was evaporated to dryness. The peptides were then resuspended in 25 μL of precolumn loading buffer (0.05 % trifluoroacetic acid (TFA) and 5 % acetonitrile (ACN) in H_2_O), prior to LC-MS/MS analysis.

### Peptide and protein identification

LC-MS/MS analysis was performed NanoLC-Ultra Eksigent (SCIEX) system connected to Q-Exactive mass spectrometer (ThermoFisher) by nanoelectrospray ion source. Tryptic peptide mixtures (4 μl) were loaded at flow rate 7.5 μl min^−1^ onto precolumn Biosphere C18, 5 μm, 20 mm, 100 μm i.d.; NanoSeparations, Nieuwkoop, NL). After 3 min, the precolumn was connected with the separating nanocolumn PepMap100C18, 3 μm, 500 mm, 75 μm i.d.; Dionex), and the linear gradient was started from 5 to 35 % of buffer B (0.1 % formic acid, 100 % acetonitrile) in buffer A (0.1 % formic acid, 5 % acetonitrile) at 300 nl min^−1^ during 120 min for a running time of 130 min with washing and equilibration steps. Ionization was performed on liquid junction with a spray voltage of 1.5 kV applied to an uncoated capillary probe (PicoTip EMITER 10-μm tip inner diameter; New Objective). Peptides ions were automatically analyzed in positive mode by the data-dependent method as follows: full MS scan (m/z 400 to 1400, resolution 70,000 at m/z 400) and MS/MS on the 12 most abundant precursors (resolution 17,500 at m/z 400). In the present study only +2 and +3 charged peptides were subjected to MS/MS experiments with an exclusion window of 40 sec, in HCD fragmentation mode with a normalized collision energy fixed to 26 %. The lock mass option was activated on m/z dimethylcyclosiloxan 445.12003.

The raw data produced on Q-Exactive mass spectrometer were first converted in mzXML file with msconvert (http://proteowizard.sourceforge.net, version 3.0.3706), and protein identification was performed with X!Tandem software (X! Tandem Sledgehammer 2013.09.01.1; http://www.thegpm.org) against a protein database of *B. subtilis* 168 (downloaded to ftp://ftp.ebi.ac.uk/pub/databases/integr8/last_release/fasta/proteomes contain 4253 proteins associated to a classical proteomic contaminant database). The X!Tandem search parameters were as follows: trypsin specificity with two missed cleavage, fixed alkylation of cysteine (+57.0215), and variable oxidation of methionine (+15.9949). The mass tolerance was fixed to 10 ppm for precursor ions and 0.02 Da for fragment ions. For all proteins identified with a protein E-value of < 0.01 in the first step, we searched for additional peptides to reinforce identification using similar parameters except that semitryptic peptides and protein N-terminal acetylations were accepted. All results for each analysis were merged with an homemade program written in java: (X!tandempipeline version 3.3.1; http://pappso.inra.fr/bioinfo/xtandempipeline/). The final search results were filtered by using a multiple threshold filter applied at the protein level and consisting of a Log10 protein E-value lower than −2.6 identified with a minimum of two different peptides sequences, detected in at least one analysis, with a peptide E-value lower than 0.05.

### Relative quantification of peptides and proteins

Control quality of data, normalization, filtration and statistical analysis were performed by using MassChroqR (http://pappso.inra.fr/bioinfo/masschroqr/). The peaks showing a width greater than 100 s or instability in retention time (RT standard deviation greater than 20 s) were filtered out. Only proteins quantified with at least two specific and repeatable peptides were analyzed. Unspecific peptides were discarded. Repeatable peptides were those which were presents in at least 7 of 8 samples. Missing values were imputed by linear regression according to the values of the other peptides of the same protein. Data were normalized to compensate for global variations between LC-MS: for each LC–MS the normalization factor was the median value of the ratios of peptide intensities to their intensity in a reference LC-MS. Protein values were computed by adding the normalized values of their specific and repeatable peptides. The proteins whose number of peaks was significantly different (with a minimum difference of 5 peaks between the mutant and the wild type) were determined by using the Kruskal-Wallis test (Additional file 9: Figure S6, Additional file 10: Figure S7, Additional file 11: Figure S8 and Additional file 12: Figure S9). A one-way ANOVA model was used to analyze changes, with the genotype as a fixed effect. A protein was considered as significantly variable when the *p*-value was < 0.05 (Additional file 4: Table S2).

### Purification of YvmB^6His^

His-tagged YvmB was over-produced as a soluble protein and purified using plasmid pJ411 (DNA2.0) and *E. coli* strain ER2566. ER2566 carries a chromosomal copy of the T7 RNA polymerase gene inserted into the *lacZ* gene, and thus is under the control of the *lac* promoter. The *yvmB* gene was amplified by PCR using a forward primer (5′-gggcatatgtctgatttgacaaaacagatg-3′) which contains a 5′ *Nde*I site and a reverse primer (5′-gggctcgagttaatggtgatggtgatggtgctttacaggtttgtctggagt-3′) which contains a His-tag followed by a *Xho*I site. The amplified fragment was digested with *Nde*I/*Xho*I and ligated *Nde*I/*Xho*I digested-pJ411 vector to construct plasmid pJ411-*yvmB*, which encodes a fusion protein containing YvmB followed by a hexa-his-tag. This plasmid was introduced into *E. coli* strain ER2566. Transformants were grown in 30 μg kanamycin ml^−1^ LB medium at 30 °C to an OD_600_ of 0.8. The expression of the *yvmB* gene was induced by adding 0.5 mM IPTG (isopropyl-β-D-thiogalactopyranoside). The cells were then incubated for an additional 3 h before they were harvested. The cell pellet was resuspended in 1 M NaCl-50 mM Tris–HCl (pH 8.0) and was broken by sonication (Bioblock Sientific, Vibra-Cell^TM^ 72408). The cell lysate was clarified by centrifugation for 90 min. at 40 000 rpm to remove cell debris. Purification was performed by stepwise elution with linear imidazole gradient (20 to 400 mM) from a Ni-affinity column. After sodium dodecyl sulfate-polyacrylamide gel electrophoresis analysis of the fractions, the fraction containing the protein was dialyzed against against 50 mM Tris–HCl (pH 8.0)-0.4 M NaCl-1 mM DTT-50 % glycerol, and stored at −20 °C.

### Denaturing gel electrophoresis

The SDS-polyacrylamide gels electrophoresis (PAGE) were 13.5 % acrylamide. The samples were loaded after being mixed with the sample buffer (125 mM Tris–HCl, 10 % glycerol, 0.1 % bromophenol blue, 2 % SDS, 1 mM DTT) with 10 min. boiling at 90 °C. Electrophoresis were done in Tris-Glycine buffer for 4 to 6 h at 15 mA.

### Gel mobility shift assay

Sequences of PCR primer pairs are shown in Additional file 7: Table S3. The Cy5 5′-labeled oligodeoxynucleotides were purchased from Eurofins Genomics (Ebersberg, Germany). PCRs were performed on DNA using Ex Taq Polimerase (Takara). Amplified fragments were gel-purified using Wizard Kit (Promega). A further PCR was run using the purified fragment as template. Electrophoretic mobility shift assay was carried out by mixing 50 ng of the desired Cy5-labelled purified DNA with a 5× binding buffer (100 mM Tris pH 8, 0.5 M KCl, 2.5 mM dithiothreitol, 25 mM MgCl2, 0.25 mg/ml BSA, 0.25 mg/ml poly(dIdC)) to a final volume of 20 μL. Different amounts of purified His-tagged YvmB were used in the reactions to obtain a clear shifted DNA band compared to the control without protein. After incubation of the reaction mixture for 20 min at 30 °C, the shift reaction was loaded onto a 6 or 10 % native polyacrylamide gel (depending on the fragment size) and run for 1 h 45 min. at 150 V. The migration of the DNA bands was visualized by a Bio-Rad Chemidoc imager with Image Lab software (Cy5-based blot protocol).

## Additional files

Additional file 1: Figure S1. Multiple sequence alignment of members of the MarR family. The alignment was generated using Multalin (Corpet, 1988, Nucleic Acids Res, 16(22):10881–10890). The aligned proteins are YvmB and OhrR from *Bacillus subtilis* and MarR from *Escherichia coli*. Residue numbering is according to entire alignment. Secondary structures elements indicated above the alignment show conservation of a winged helix-turn-helix (wHTH) motif and are based on the *E. coli* MarR crystal structure, with α-helices represented as open green boxes, β-strands open green arrows and the wing as a filled green box [58]. The HTH domain corresponds to helices α3 and α4, with α4 constituting the recognition helix. The β-hairpin “wing” motif is formed by the β2, W1, β3 structural elements. Helices α1, α5 and α6 form the dimerization domain. (PDF 81 kb) Additional file 2: Figure S2. Effect of iron starvation on the expression of *cypX* and *yvmB*. The BFA815 (*cypX::lacZ*) and BSAS108 (p*yvmB’*-*lacZ*) strains were cultivated in LB medium until OD_600_ of 1. Samples of 2 ml of the cultures were spread onto solid LB medium containing 20 μg.ml^−1^ X-gal. A drop of 10 μl 10 mM bipyridyl was deposited at the center of each plate. Blue rings corresponded to expression of the fusion in cells around the inhibition zone of bipyridyl drops. (PDF 75 kb) Additional file 3: Table S1. Whole proteins detected in the membrane and cytosolic fractions from *B. subtilis* wild-type and Δ*yvmB* strains. (XLS 601 kb) Additional file 4: TableS2. Proteins differentially abundant in *B. subtilis* wild-type and Δ*yvmB* strains. (XLS 47 kb) Additional file 5: Figure S3. The *yvmA*-*yvmC-cypX* locus from *B. subtilis* and *B. licheniformis* ATCC 14580. (A) For each gene product of *B. subtilis* and its equivalent in *B. licheniformis* ATCC 14580 a percentage of identity is indicated. The position of the genes on each chromosome is given. The *yvmC* and *cypX* genes encoding the enzymes involved in pulcherriminic synthesis are represented by purple arrows, the MarR-type regulators YvmB by brown arrows, and the MFS-like transporters YvmA by white arrows. The conserved palindromic motif, palindrome I, is indicated by blue boxes. (B) Alignment of the 42-bp palindromic motifs from the promoter regions of *B. subtilis yvmB* and *B. licheniformis yvmA* genes. Stars indicate the seven nucleotides, which are not conserved between the two palindromic sequences. The 14 bp core region highly conserved in the two sequences is boxed. (PDF 147 kb) Additional file 6: Figure S4. Purification and analysis of YvmB^His-tag^ protein. (A) SDS-PAGE analysis of purified YvmB^His-tag^ protein from *E. coli* cells. (B) Fractionated chromatography on a Superdex 200 h/300 gel filtration column. The YvmB^His-tag^ protein eluted as a major peak with an apparent molecular mass of 45 ± 5 kDa. (PDF 164 kb) Additional file 7: Table S3. List of probes used in the gel shift assays. (DOC 32 kb) Additional file 8: Figure S5. Promoter regions of the divergent *yvnA*-*yvnB* genes and of the *yisI* gene. (A) The −10 and −35 regions of *yvnA* (in purple) and the −10 and −35 regions of *yvnB* (in brown) are indicated according to Nicolas et al. [33]. The predicted YvmB box is indicated in red and a degenerated YvmB box motif is indicated in orange. (B) The −10 and −35 regions of *yisI* (in brown) is indicated according to Nicolas et al. [33]. The prdicted YvmB box is indicated in red. (PDF 84 kb) Additional file 9: Figure S6. Proteins differing significantly in Δ*yvmB* cells from proteome analysis of the cytosolic fraction. A Kruskal*-*Wallis one-way analysis of variance (ANOVA) was done on the whole proteomic data. (PDF 20 kb) Additional file 10: Figure S7. Proteins differing significantly in Δ*yvmB* cells from proteome analysis of the membrane fraction. A Kruskal*-*Wallis one-way analysis of variance (ANOVA) was done on the whole proteomic data. (PDF 13 kb) Additional file 11: Figure S8. Heatmap representation of proteins that were found differentially abundant between the Δ*yvmB* mutant and the wild type strain in the cytosolic fraction. Heatmap shows the change in protein levels among four independent samples. Protein levels are indicated by yellow to red colouring, a red shade indicates a higher abundance level. (PDF 7 kb) Additional file 12: Figure S9. Heatmap representation of proteins that were found differentially abundant between the Δ*yvmB* mutant and the wild type strain in the membrane fraction. Heatmap shows the change in protein levels among four independent samples. Protein levels are indicated by yellow to red colouring, a red shade indicates a higher abundance level. (PDF 6 kb)

## References

[1] Vazquez-Rivera D, Gonzalez O, Guzman-Rodriguez J, Diaz-Perez AL, Ochoa-Zarzosa A, Lopez-Bucio J, Meza-Carmen V, Campos-Garcia J (2015). Cytotoxicity of cyclodipeptides from *Pseudomonas aeruginosa* PAO1 leads to apoptosis in human cancer cell lines. Biomed Res Int.

[2] Fukushima K, Yazawa K, Arai T (1973). Biological activities of albonoursin. J Antibiot (Tokyo).

[3] Macwilliam IC (1959). A survey of the antibiotic powers of yeasts. J Gen Microbiol.

[4] Oro L, Ciani M, Comitini F (2014). Antimicrobial activity of *Metschnikowia pulcherrima* on wine yeasts. J Appl Microbiol.

[5] Belin P, Le Du MH, Fielding A, Lequin O, Jacquet M, Charbonnier JB, Lecoq A, Thai R, Courcon M, Masson C (2009). Identification and structural basis of the reaction catalyzed by CYP121, an essential cytochrome P450 in *Mycobacterium tuberculosis*. Proc Natl Acad Sci U S A.

[6] McLean KJ, Carroll P, Lewis DG, Dunford AJ, Seward HE, Neeli R, Cheesman MR, Marsollier L, Douglas P, Smith WE (2008). Characterization of active site structure in CYP121. A cytochrome P450 essential for viability of *Mycobacterium tuberculosis* H37Rv. J Biol Chem.

[7] Canale-Parola E (1963). A red pigment produced by aerobic sporeforming bacteria. Arch Mikrobiol.

[8] Kluyver AJ, van der Walt JP, van Triet AJ (1953). Pulcherrimin, the pigment of *Candida pulcherrima*. Proc Natl Acad Sci U S A.

[9] MacDonald JC (1965). Biosynthesis of pulcherriminic acid. Biochem J.

[10] Uffen RL, Canale-Parola E (1972). Synthesis of pulcherriminic acid by *Bacillus subtilis*. J Bacteriol.

[11] Sipiczki M (2006). *Metschnikowia* strains isolated from botrytized grapes antagonize fungal and bacterial growth by iron depletion. Appl Environ Microbiol.

[12] Turkel S, Ener B (2009). Isolation and characterization of new *Metschnikowia pulcherrima* strains as producers of the antimicrobial pigment pulcherrimin. Z Naturforsch C.

[13] Turkel S, Korukluoglu M, Yavuz M (2014). Biocontrol activity of the local strain of *Metschnikowia pulcherrima* on different postharvest pathogens. Biotechnol Res Int.

[14] Gondry M, Sauguet L, Belin P, Thai R, Amouroux R, Tellier C, Tuphile K, Jacquet M, Braud S, Courcon M (2009). Cyclodipeptide synthases are a family of tRNA-dependent peptide bond-forming enzymes. Nat Chem Biol.

[15] Sauguet L, Moutiez M, Li Y, Belin P, Seguin J, Le Du MH, Thai R, Masson C, Fonvielle M, Pernodet JL (2011). Cyclodipeptide synthases, a family of class-I aminoacyl-tRNA synthetase-like enzymes involved in non-ribosomal peptide synthesis. Nucleic Acids Res.

[16] Cryle MJ, Bell SG, Schlichting I (2010). Structural and biochemical characterization of the cytochrome P450 CypX (CYP134A1) from *Bacillus subtilis*: a cyclo-L-leucyl-L-leucyl dipeptide oxidase. Biochemistry.

[17] Alekshun MN, Levy SB (1999). The *mar* regulon: multiple resistance to antibiotics and other toxic chemicals. Trends Microbiol.

[18] Egland PG, Harwood CS (1999). BadR, a new MarR family member, regulates anaerobic benzoate degradation by *Rhodopseudomonas palustris* in concert with AadR, an Fnr family member. J Bacteriol.

[19] Fuangthong M, Atichartpongkul S, Mongkolsuk S, Helmann JD (2001). OhrR is a repressor of *ohrA*, a key organic hydroperoxide resistance determinant in *Bacillus subtilis*. J Bacteriol.

[20] Otani H, Stogios PJ, Xu X, Nocek B, Li SN, Savchenko A, Eltis LD. The activity of CouR, a MarR family transcriptional regulator, is modulated through a novel molecular mechanism. Nucleic Acids Res. 2016;44(2):595-607. 10.1093/nar/gkv955 PMC4737184 26400178

[21] Seoane AS, Levy SB (1995). Characterization of MarR, the repressor of the multiple antibiotic resistance (*mar*) operon in *Escherichia coli*. J Bacteriol.

[22] Wilkinson SP, Grove A (2004). HucR, a novel uric acid-responsive member of the MarR family of transcriptional regulators from *Deinococcus radiodurans*. J Biol Chem.

[23] Ellison DW, Miller VL (2006). Regulation of virulence by members of the MarR/SlyA family. Curr Opin Microbiol.

[24] Lim D, Poole K, Strynadka NC (2002). Crystal structure of the MexR repressor of the *mexRAB-oprM* multidrug efflux operon of *Pseudomonas aeruginosa*. J Biol Chem.

[25] Luong TT, Newell SW, Lee CY (2003). Mgr, a novel global regulator in *Staphylococcus aureus*. J Bacteriol.

[26] Wei K, Tang DJ, He YQ, Feng JX, Jiang BL, Lu GT, Chen B, Tang JL (2007). *hpaR*, a putative *marR* family transcriptional regulator, is positively controlled by HrpG and HrpX and involved in the pathogenesis, hypersensitive response, and extracellular protease production of *Xanthomonas campestris* pathovar campestris. J Bacteriol.

[27] Di Fiore A, Fiorentino G, Vitale RM, Ronca R, Amodeo P, Pedone C, Bartolucci S, De Simone G (2009). Structural analysis of BldR from *Sulfolobus solfataricus* provides insights into the molecular basis of transcriptional activation in Archaea by MarR family proteins. J Mol Biol.

[28] Ludwig M, Pandelia ME, Chew CY, Zhang B, Golbeck JH, Krebs C, Bryant DA (2014). ChlR protein of *Synechococcus* sp. PCC 7002 is a transcription activator that uses an oxygen-sensitive [4Fe-4S] cluster to control genes involved in pigment biosynthesis. J Biol Chem.

[29] Oh SY, Shin JH, Roe JH (2007). Dual role of OhrR as a repressor and an activator in response to organic hydroperoxides in *Streptomyces coelicolor*. J Bacteriol.

[30] Grove A (2013). MarR family transcription factors. Curr Biol.

[31] Perera IC, Grove A (2010). Molecular mechanisms of ligand-mediated attenuation of DNA binding by MarR family transcriptional regulators. J Mol Cell Biol.

[32] Wilkinson SP, Grove A (2006). Ligand-responsive transcriptional regulation by members of the MarR family of winged helix proteins. Curr Issues Mol Biol.

[33] Nicolas P, Mader U, Dervyn E, Rochat T, Leduc A, Pigeonneau N, Bidnenko E, Marchadier E, Hoebeke M, Aymerich S (2012). Condition-dependent transcriptome reveals high-level regulatory architecture in *Bacillus subtilis*. Science.

[34] Blencke HM, Homuth G, Ludwig H, Mader U, Hecker M, Stülke J (2003). Transcriptional profiling of gene expression in response to glucose in *Bacillus subtilis*: regulation of the central metabolic pathways. Metab Eng.

[35] Smaldone GT, Antelmann H, Gaballa A, Helmann JD (2012). The FsrA sRNA and FbpB protein mediate the iron-dependent induction of the *bacillus subtilis* LutABC iron-sulfur-containing oxidases. J Bacteriol.

[36] Yu WB, Ye BC. Transcriptional profiling analysis of *Bacillus subtilis* in response to high levels of Fe. Curr Microbiol. 2016. 10.1007/s00284-016-0998-8 26858131

[37] Alen C, Sonenshein AL (1999). *Bacillus subtilis* aconitase is an RNA-binding protein. Proc Natl Acad Sci U S A.

[38] Gaballa A, Antelmann H, Aguilar C, Khakh SK, Song KB, Smaldone GT, Helmann JD (2008). The *Bacillus subtilis* iron-sparing response is mediated by a Fur-regulated small RNA and three small, basic proteins. Proc Natl Acad Sci U S A.

[39] Pechter KB, Meyer FM, Serio AW, Stulke J, Sonenshein AL (2013). Two roles for aconitase in the regulation of tricarboxylic acid branch gene expression in *Bacillus subtilis*. J Bacteriol.

[40] Miethke M, Westers H, Blom EJ, Kuipers OP, Marahiel MA (2006). Iron starvation triggers the stringent response and induces amino acid biosynthesis for bacillibactin production in *Bacillus subtilis*. J Bacteriol.

[41] Baichoo N, Wang T, Ye R, Helmann JD (2002). Global analysis of the *Bacillus subtilis* Fur regulon and the iron starvation stimulon. Mol Microbiol.

[42] Herbig AF, Helmann JD (2001). Roles of metal ions and hydrogen peroxide in modulating the interaction of the *Bacillus subtilis* PerR peroxide regulon repressor with operator DNA. Mol Microbiol.

[43] Chamnongpol S, Dodson W, Cromie MJ, Harris ZL, Groisman EA (2002). Fe(III)-mediated cellular toxicity. Mol Microbiol.

[44] Suwalsky M, Martinez F, Cardenas H, Grzyb J, Strzalka K (2005). Iron affects the structure of cell membrane molecular models. Chem Phys Lipids.

[45] Wosten MM, Kox LF, Chamnongpol S, Soncini FC, Groisman EA (2000). A signal transduction system that responds to extracellular iron. Cell.

[46] Bailey TL, Williams N, Misleh C, Li WW (2006). MEME: discovering and analyzing DNA and protein sequence motifs. Nucleic Acids Res.

[47] Perego M (2001). A new family of aspartyl phosphate phosphatases targeting the sporulation transcription factor Spo0A of *Bacillus subtilis*. Mol Microbiol.

[48] Kupfer DG, Uffen RL, Canale-Parola E (1967). The role of iron and molecular oxygen in pulcherrimin synthesis by bacteria. Arch Mikrobiol.

[49] Cook AH, Slater CA (1956). The structure of pulcherrimin. J Chem Soc.

[50] Saravanakumar D, Spadaro D, Garibaldi A, Gullino ML (2009). Detection of enzymatic activity and partial sequence of a chitinase gene in *Metschnikowia pulcherrima* strain MACH1 used as post-harvest biocontrol agent. Eur J Plant Pathol.

[51] Sambrook J, Fristch EF, Maniatis T (1989). Molecular cloning: a laboratory manual.

[52] Kunst F, Rapoport G (1995). Salt stress is an environmental signal affecting degradative enzyme synthesis in *Bacillus subtilis*. J Bacteriol.

[53] Stülke J, Martin-Verstraete I, Zagorec M, Rose M, Klier A, Rapoport G (1997). Induction of the *Bacillus subtilis ptsGHI* operon by glucose is controlled by a novel antiterminator, GlcT. Mol Microbiol.

[54] Miller JH (1972). Assay of B-galactosidase.

[55] Wach A (1996). PCR-synthesis of marker cassettes with long flanking homology regions for gene disruptions in *S. cerevisiae*. Yeast.

[56] Gibson DG, Young L, Chuang RY, Venter JC, Hutchison CA, Smith HO (2009). Enzymatic assembly of DNA molecules up to several hundred kilobases. Nat Methods.

[57] Mirouze N, Prepiak P, Dubnau D (2011). Fluctuations in spo0A transcription control rare developmental transitions in *Bacillus subtilis*. PLoS Genet.

[58] Alekshun MN, Levy SB, Mealy TR, Seaton BA, Head JF. The crystal structure of MarR, a regulator of multiple antibiotic resistance, at 2.3 A resolution. Nat Struct Biol. 2001;8(8):710-4. 10.1038/90429 11473263

