# Supplementary figures and images for: The MarR-like protein PchR (YvmB) regulates expression of genes involved in pulcherriminic acid biosynthesis and in the initiation of sporulation in Bacillus subtilis
Source: BMC Microbiol. 2016 Aug 20;16:190. doi: 10.1186/s12866-016-0807-3 (PMC4992311; doi:10.1186/s12866-016-0807-3)

Figure S1

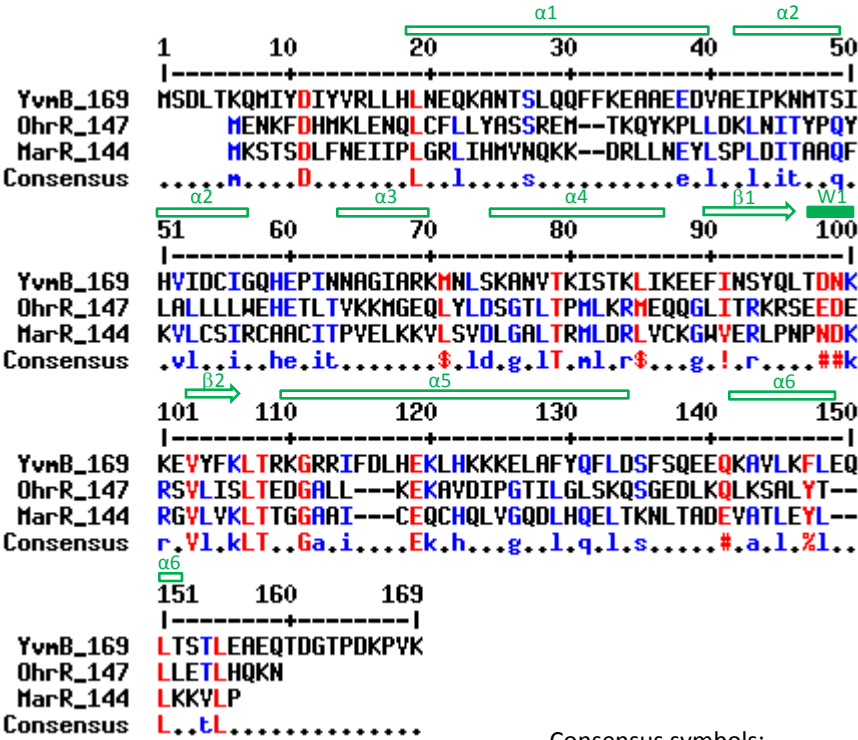

Consensus symbols:  
! is anyone of IV  
\$ is anyone of LM  
% is anyone of FY  
# is anyone of NDQEBZ

Supplement: Additional file 1: Figure S1. — Multiple sequence alignment of members of the MarR family. The alignment was generated using Multalin (Corpet, 1988, Nucleic Acids Res, 16(22):10881–10890). The aligned proteins are YvmB and OhrR from Bacillus subtilis and MarR from Escherichia coli. Residue numbering is according to entire alignment. Secondary structures elements indicated above the alignment show conservation of a winged helix-turn-helix (wHTH) motif and are based on the E. coli MarR crystal structure, with α-helices represented as open green boxes, β-strands open green arrows and the wing as a filled green box [58]. The HTH domain corresponds to helices α3 and α4, with α4 constituting the recognition helix. The β-hairpin “wing” motif is formed by the β2, W1, β3 structural elements. Helices α1, α5 and α6 form the dimerization domain. (PDF 81 kb) [file 12866_2016_807_MOESM1_ESM.pdf]

**Figure S2**

***cypX::lacZ* in WT**

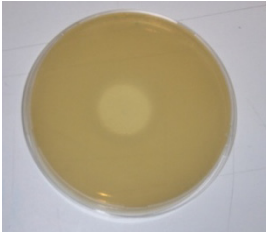

***pyvmB'-lacZ* in WT**

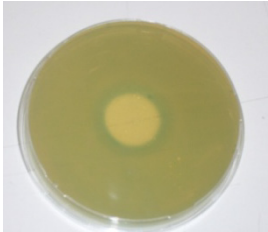

Supplement: Additional file 2: Figure S2. — Effect of iron starvation on the expression of cypX and yvmB. The BFA815 (cypX::lacZ) and BSAS108 (pyvmB’-lacZ) strains were cultivated in LB medium until OD600 of 1. Samples of 2 ml of the cultures were spread onto solid LB medium containing 20 μg.ml−1 X-gal. A drop of 10 μl 10 mM bipyridyl was deposited at the center of each plate. Blue rings corresponded to expression of the fusion in cells around the inhibition zone of bipyridyl drops. (PDF 75 kb) [file 12866_2016_807_MOESM2_ESM.pdf]

Figure S4

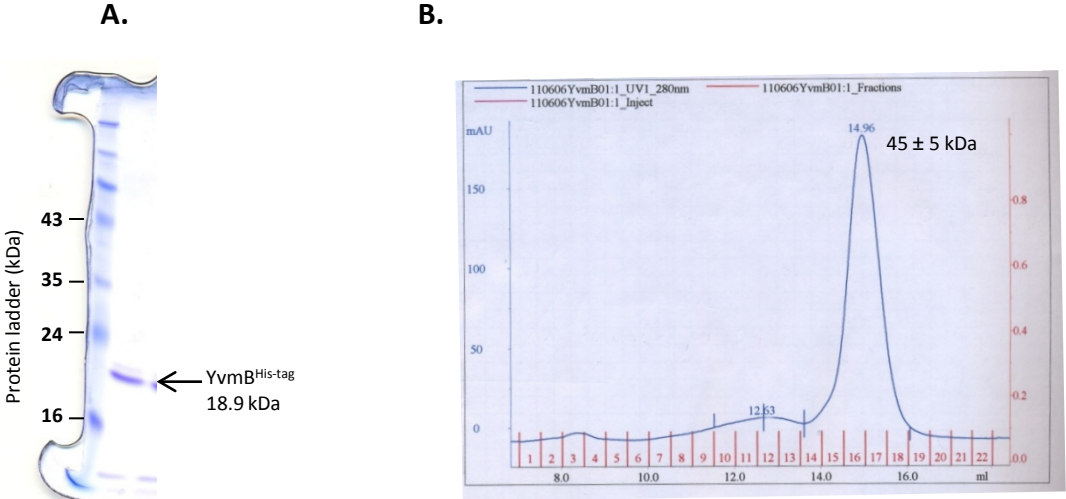

Supplement: Additional file 6: Figure S4. — Purification and analysis of YvmBHis-tag protein. (A) SDS-PAGE analysis of purified YvmBHis-tag protein from E. coli cells. (B) Fractionated chromatography on a Superdex 200 h/300 gel filtration column. The YvmBHis-tag protein eluted as a major peak with an apparent molecular mass of 45 ± 5 kDa. (PDF 164 kb) [file 12866_2016_807_MOESM6_ESM.pdf]

Color Key  
and Histogram

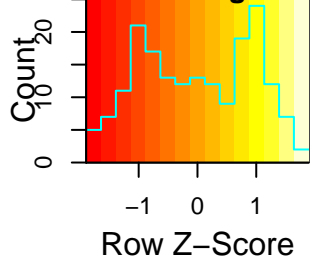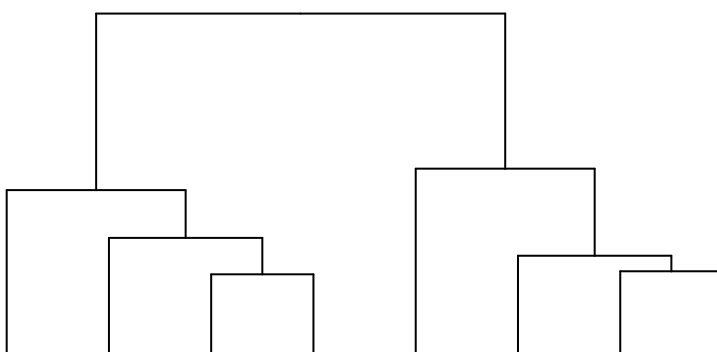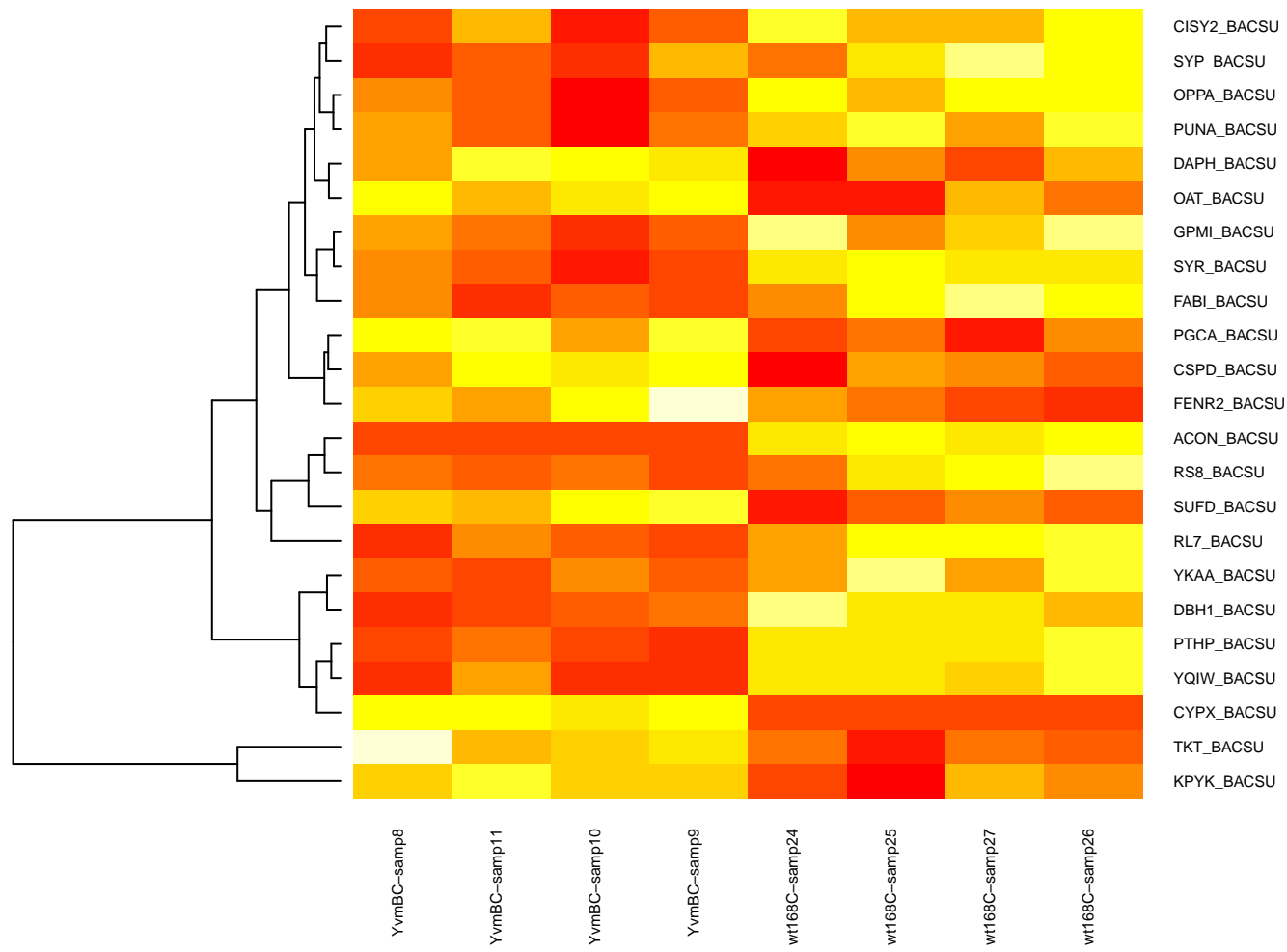

Supplement: Additional file 11: Figure S8. — Heatmap representation of proteins that were found differentially abundant between the ΔyvmB mutant and the wild type strain in the cytosolic fraction. Heatmap shows the change in protein levels among four independent samples. Protein levels are indicated by yellow to red colouring, a red shade indicates a higher abundance level. (PDF 7 kb) [file 12866_2016_807_MOESM11_ESM.pdf]

Color Key

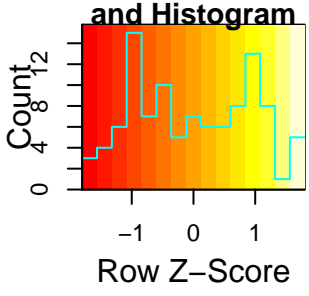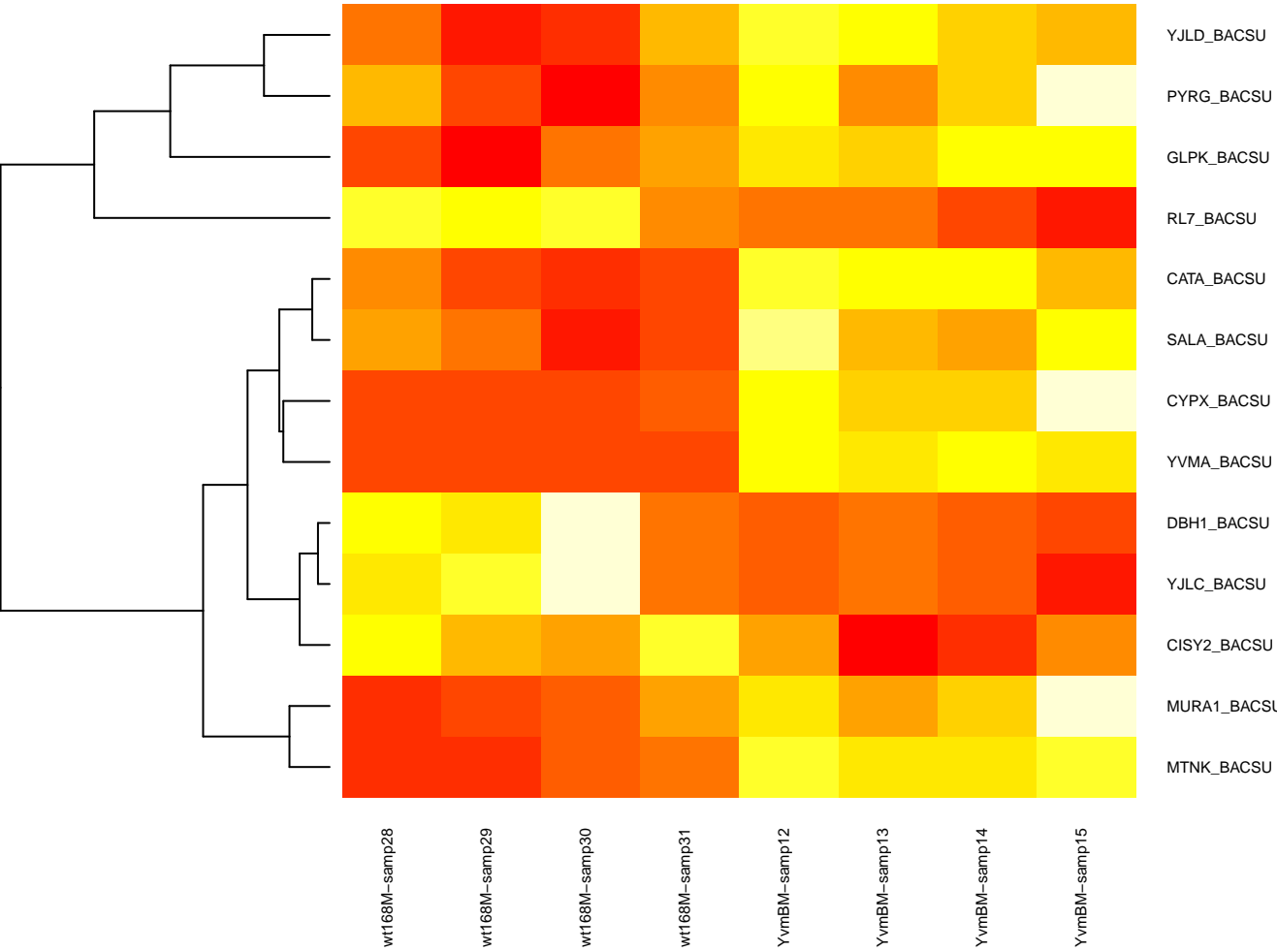

Supplement: Additional file 12: Figure S9. — Heatmap representation of proteins that were found differentially abundant between the ΔyvmB mutant and the wild type strain in the membrane fraction. Heatmap shows the change in protein levels among four independent samples. Protein levels are indicated by yellow to red colouring, a red shade indicates a higher abundance level. (PDF 6 kb) [file 12866_2016_807_MOESM12_ESM.pdf]
