# Supplementary material for: The MarR-like protein PchR (YvmB) regulates expression of genes involved in pulcherriminic acid biosynthesis and in the initiation of sporulation in Bacillus subtilis
Source: BMC Microbiol. 2016 Aug 20;16:190. doi: 10.1186/s12866-016-0807-3 (PMC4992311; doi:10.1186/s12866-016-0807-3)
Supplement: Additional file 5: Figure S3. — The yvmA-yvmC-cypX locus from B. subtilis and B. licheniformis ATCC 14580. (A) For each gene product of B. subtilis and its equivalent in B. licheniformis ATCC 14580 a percentage of identity is indicated. The position of the genes on each chromosome is given. The yvmC and cypX genes encoding the enzymes involved in pulcherriminic synthesis are represented by purple arrows, the MarR-type regulators YvmB by brown arrows, and the MFS-like transporters YvmA by white arrows. The conserved palindromic motif, palindrome I, is indicated by blue boxes. (B) Alignment of the 42-bp palindromic motifs from the promoter regions of B. subtilis yvmB and B. licheniformis yvmA genes. Stars indicate the seven nucleotides, which are not conserved between the two palindromic sequences. The 14 bp core region highly conserved in the two sequences is boxed. (PDF 147 kb) [file 12866_2016_807_MOESM5_ESM.pdf]

## Figure S3

**A.**

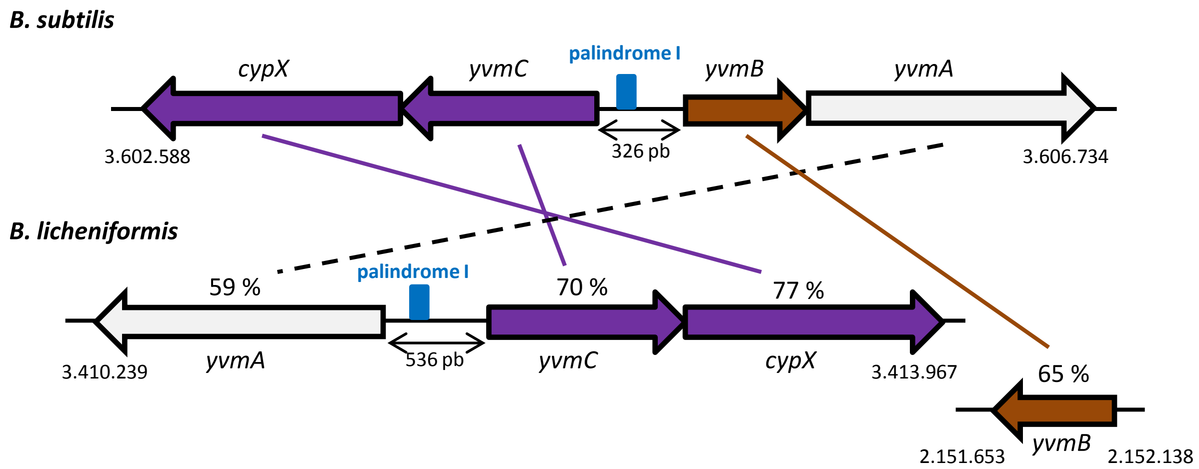


**B.**

```
yvmB B. subtilis       ATATCATGATAAATGTTTACTAGTAAACATTAGTCATTTTAT
yvmA B. licheniformis  GTTACAGCATAAAAGTTTACTAGTAAACAATAGTCATTTTAT
                       * **  **     *               *
```
