# Supplementary material for: The MarR-like protein PchR (YvmB) regulates expression of genes involved in pulcherriminic acid biosynthesis and in the initiation of sporulation in Bacillus subtilis
Source: BMC Microbiol. 2016 Aug 20;16:190. doi: 10.1186/s12866-016-0807-3 (PMC4992311; doi:10.1186/s12866-016-0807-3)
Supplement: Additional file 7: Table S3. — List of probes used in the gel shift assays. (DOC 32 kb) [file 12866_2016_807_MOESM7_ESM.doc]

Table S3. List of probes used in the gel shift assays.

| Probe | Sequence |
| --- | --- |
| P1, 110 bp | TTCCAAATGTTTTTTAAGATTTTAATAATCATTTTCAATACCCATATCATGATAAAT**GTTTACTAGTAAAC**ATTAGTCATTTTATCCCTTTTCCTTCCAAACGTCAATAT |
| P2, 110 bp | CCAACAATTGACAACGAAAACAAGAAAAGATAAAATAAAATT**GTTCACCAGTAAAC**GATTTGTTTTATT**GTTTCATCGTAAAC**TATTATTCCTAAGAAAGAAGGTAGCCT |
| P3, 328 bp | TTCCAAATGTTTTTTAAGATTTTAATAATCATTTTCAATACCCATATCATGATAAAT**GTTTACTAGTAAAC**ATTAGTCATTTTATCCCTTTTCCTTCCAAACGTCAATATgatctgtgcccaaatatttgaaaataaatgtaaacatcacattttccacacactttttacacgcatattcagcatgacatatcaacaaatagtctaaacaccaacaattgacaacgaaaacAAGAAAACAAGAAAAGATAAAATAAAATT**GTTCACCAGTAAAC**GATTTGTTTTATT**GTTTCATCGTAAAC**TATTATTCCTAAGAAAGAAGGTAGCCT |
| P-yvnB, 110 bp | GAATGCTGTTGTCCATCAATTTGCCTCTCTGATTTAATT**GTTAACTGGTTAAC**TATCCTTA**GTTTACTGGGTAAT**AGTGGAATTTTCAAGAGACAGACGTTTTTTTGTTC |
| P-yxnA, 110 bp | TTTCTGGCAAAAACCGCTTCCCAGCTTTTAAACACCTTCATGCAAACCGTCCGCAGCG**GTTCACTGATTAACG**CTGTCTCACTCGCCAGCAAACCGACTGGAATACCTTT |
| P-ndhF, 110 bp | CATCTATAATGGGTTGGATTCTTTTAGAGGAAAGTAAAGACCA**GTTTACTGATAAAC**TTGACAACTTTTAAAATTTAGCATATCTTATACGTAACTTAATAATCGATGCA |
| P-yckD, 110 bp | AGGTCAGTCTGTTAACAATGTTTTTACGGGTTATTGTAGCCGGCCTG**GTTTACTGTATAAC**CTTCGGACTCGGCTTGATCGCCAGCCTGATTCTGATTGCCGTACGAGAA |
| P-yisI, 110 bp | AGGTCGCAATATCACACCTTAATGAAAAACGTGCTGAAGCGGTC**GTTTACTATTGAAC**GATTATCTCCTCTCATCAACGAATGGCACCTTGACATTTGTCAAAGTGCTGC |

In red: sequences of the YvmB-box motifs detected upstream of *yvmB*, *yvnB*, *yxnA*, *yckD* and *yisI* genes.

In orange: sequence of a degenerated YvmB-box motifs detected upstream of the *yvnB* gene.
