# Supplementary material for: The MarR-like protein PchR (YvmB) regulates expression of genes involved in pulcherriminic acid biosynthesis and in the initiation of sporulation in Bacillus subtilis
Source: BMC Microbiol. 2016 Aug 20;16:190. doi: 10.1186/s12866-016-0807-3 (PMC4992311; doi:10.1186/s12866-016-0807-3)
Supplement: Additional file 8: Figure S5. — Promoter regions of the divergent yvnA-yvnB genes and of the yisI gene. (A) The −10 and −35 regions of yvnA (in purple) and the −10 and −35 regions of yvnB (in brown) are indicated according to Nicolas et al. [33]. The predicted YvmB box is indicated in red and a degenerated YvmB box motif is indicated in orange. (B) The −10 and −35 regions of yisI (in brown) is indicated according to Nicolas et al. [33]. The prdicted YvmB box is indicated in red. (PDF 84 kb) [file 12866_2016_807_MOESM8_ESM.pdf]

Figure S5

A.

AGC TTG AAA TAA TTC ATC ATG AAT GCT GTT GTC CAT CAATTTGCCTCTCT
GTTAAACGGAGAGA

yvnA

YvmB box — degenerated YvmB box

GATTTAATTGTTAACTGGTTAACTATCCTTAGTTTACTGGGTAATAGTGGAATTTTCAAGAG
CTAAATTAACAATTGACCAATTGATAGGAATCAAATGACCCATTATCACCTTAAAAGTTCTC

-10 -35

-35 -10

ACAGACGTTTTTTTGTTCTTTATGCACAAAAATGAACTCATAAGCTTCCCTGTTCAGACGCA
TGTCTGCAAAAAAACAAGAAATACGTGTTTTTACTTGAGTATTCGAAGGGACAAGTCTGCGT

yvnB

-10

AAATACATGAAAAGGGAGGTGCTTTG ATG AGG AAA TAC ACG GTT ATT GCT TCT
TTTATGTACTTTTCCCTCCACGAAAC

B.

YvmB box

AATGAAAAACGTGCTGAAGCGGTCGTTTACTATTGAACGATTATCTCCTCTCATCAACGAAT

GGCACCTTGACATTTGTCAAAGTGCTGCTGATGATCCTTTTATCAAAAAAAGCCCCTTTCAA

ATCGAAAAGGAGCAGACCAACATACTTAGAGAAATTGAGGAACGGCAGGACTTTTTGCAAGC

ACACTTAGCAAGACTTTAGTCCTTTCTCCATTAAAAAAGGCCGCAGCAAAAATTGTCGCTTT

TTTAGTCAAATTGACGAAAGGCAACCTTTTGAGCCAAAATAGGTTATACTAACGATATCCTG

yisI

TATTTATATCAAAATAAAATCTAGAATTGGAGATAGCGTA ATG AAC AGT AAA ATT
