# Supplementary material for: The MarR-like protein PchR (YvmB) regulates expression of genes involved in pulcherriminic acid biosynthesis and in the initiation of sporulation in Bacillus subtilis
Source: BMC Microbiol. 2016 Aug 20;16:190. doi: 10.1186/s12866-016-0807-3 (PMC4992311; doi:10.1186/s12866-016-0807-3)
Supplement: Additional file 9: Figure S6. — Proteins differing significantly in ΔyvmB cells from proteome analysis of the cytosolic fraction. A Kruskal-Wallis one-way analysis of variance (ANOVA) was done on the whole proteomic data. (PDF 20 kb) [file 12866_2016_807_MOESM9_ESM.pdf]

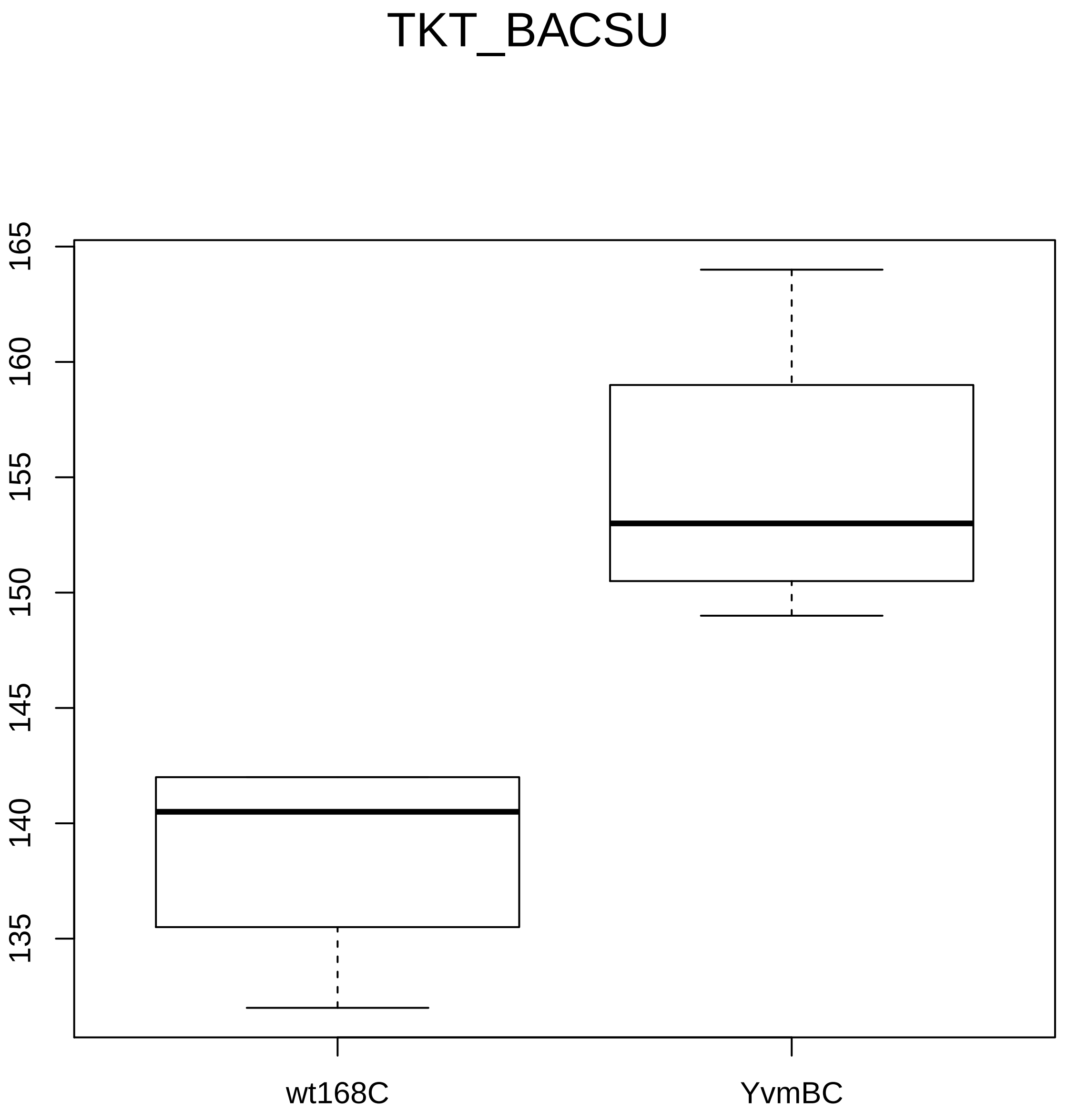
TKT_BACSU
165
160
155
150
145
140
135
wt168C
YvmBC

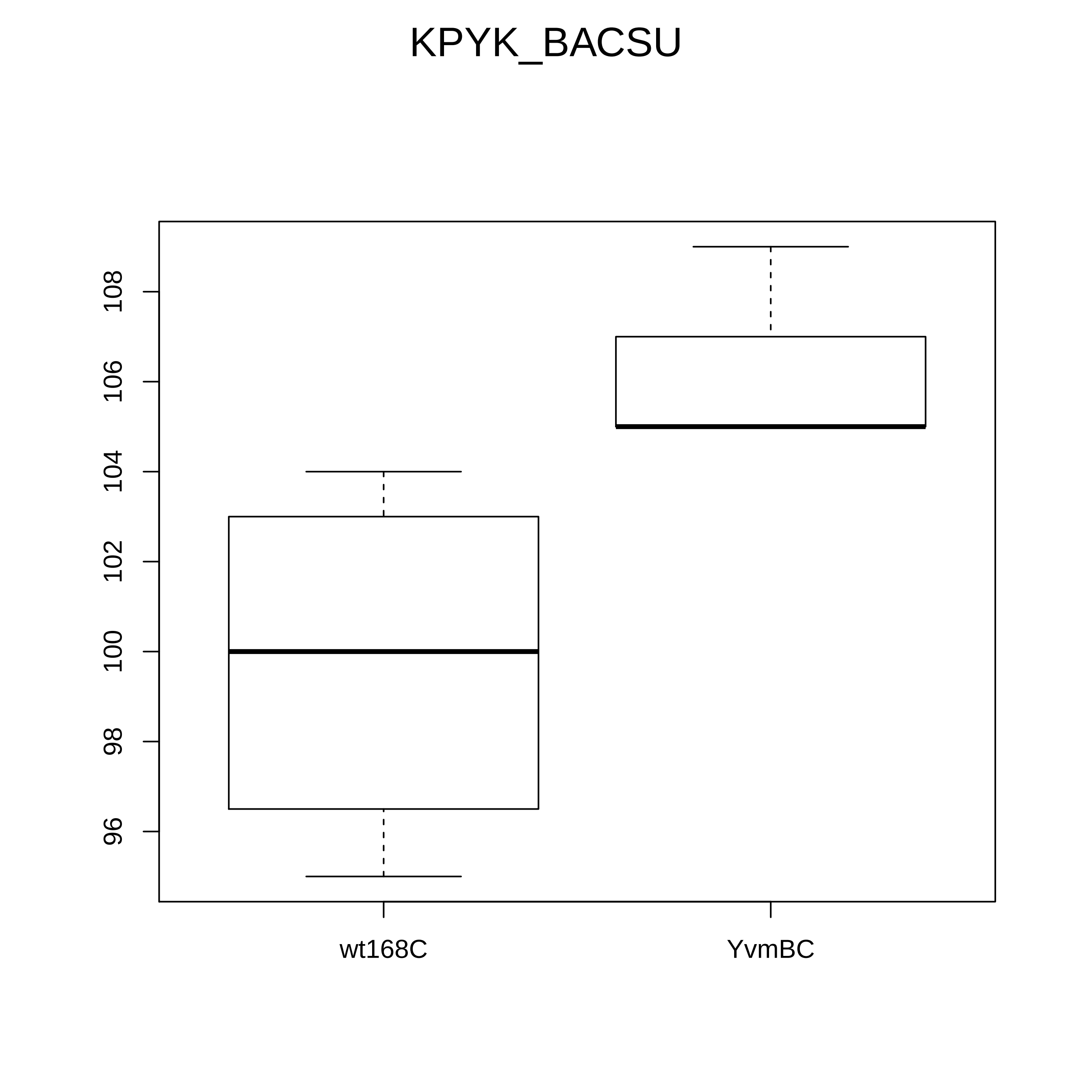
KPYK_BACSU
96
98
100
102
104
106
108
wt168C
YvmBC

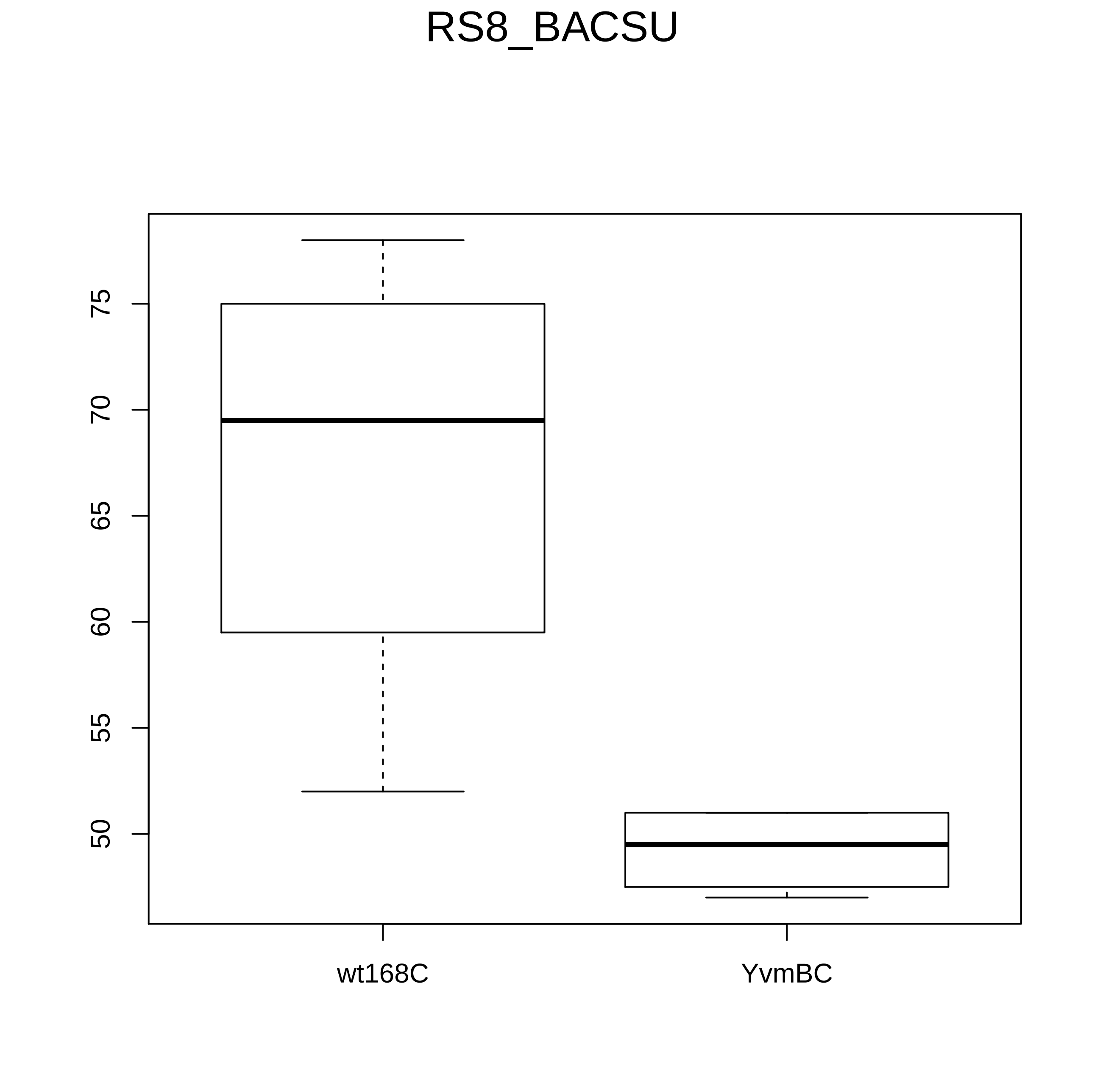
RS8_BACSU
50
55
60
65
70
75
wt168C
YvmBC

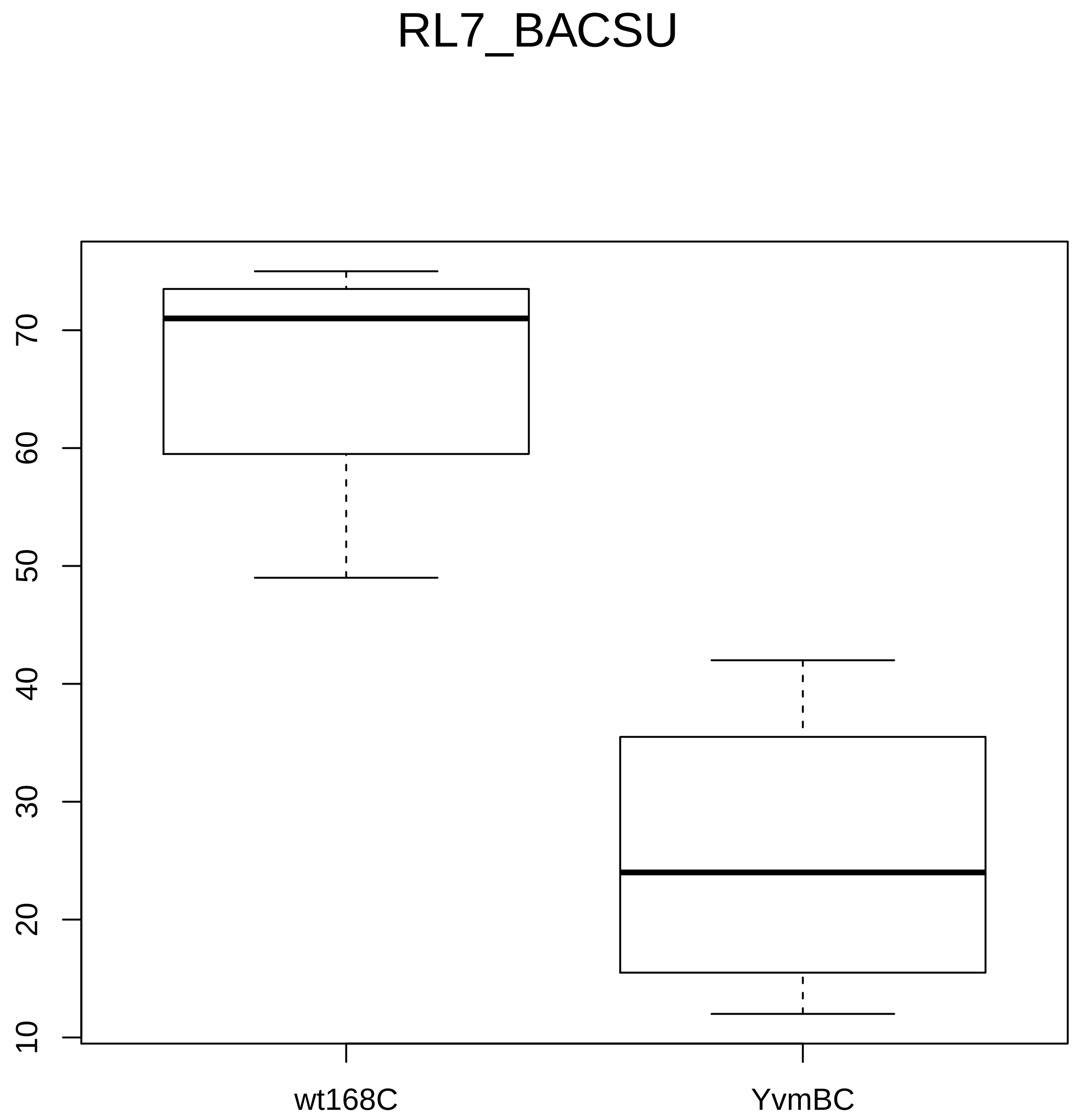

RL7_BACSU
10
20
30
40
50
60
70
wt168C
YvmBC

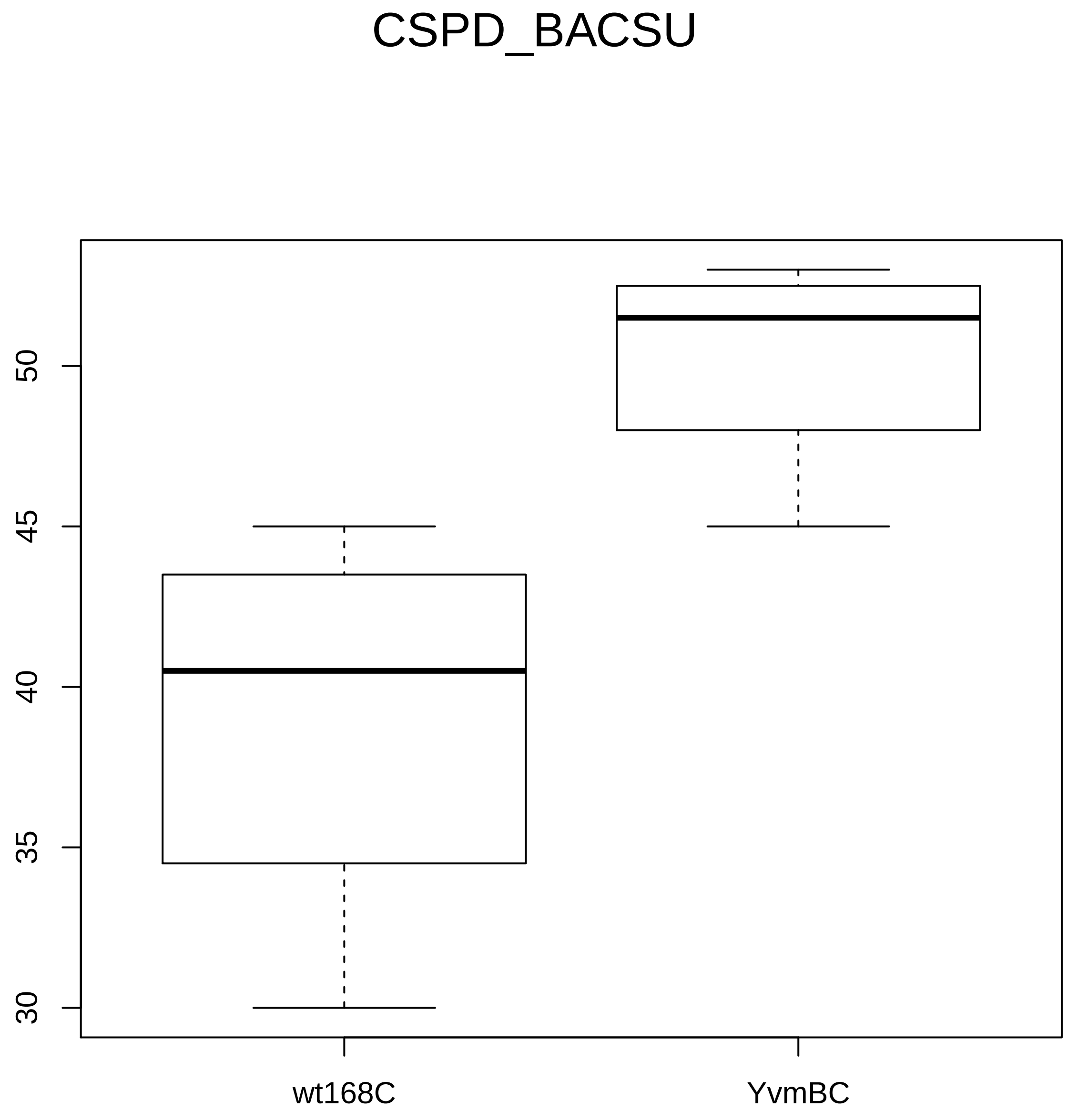

CSPD_BACSU
30
35
40
45
50
wt168C
YvmBC

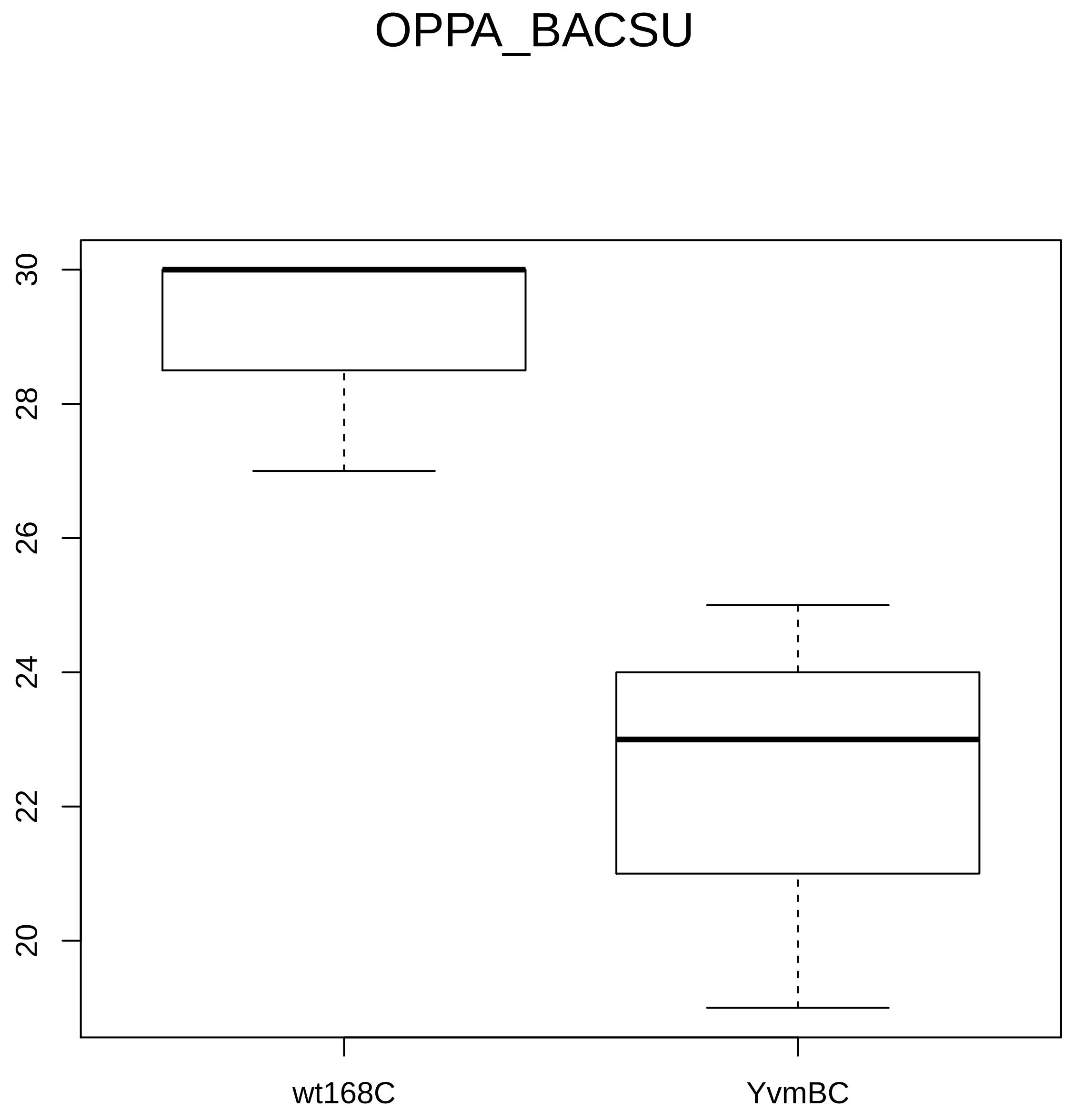
OPPA_BACSU
30
28
26
24
22
20
wt168C
YvmBC

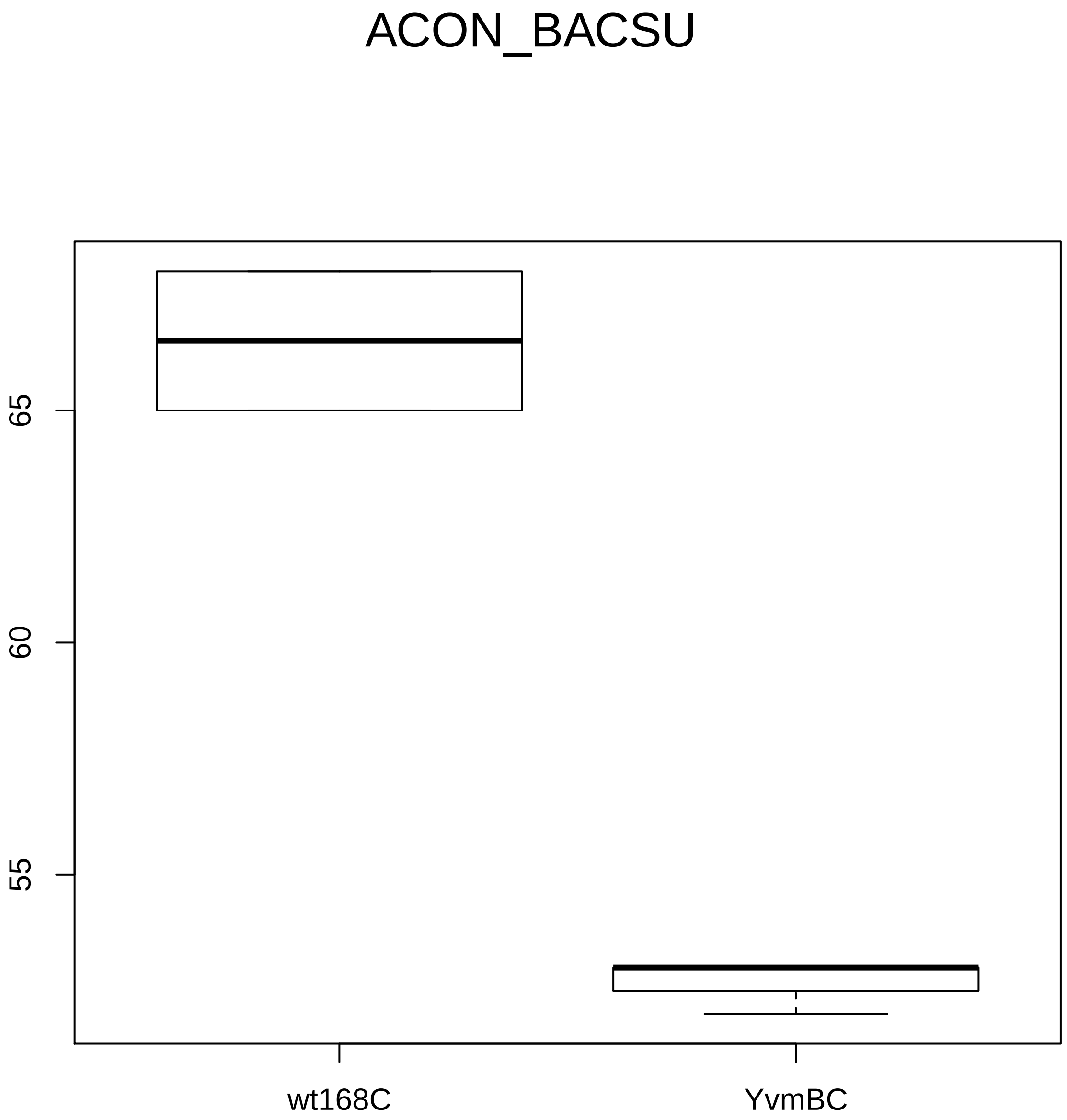
ACON_BACSU
55
60
65
wt168C
YvmBC

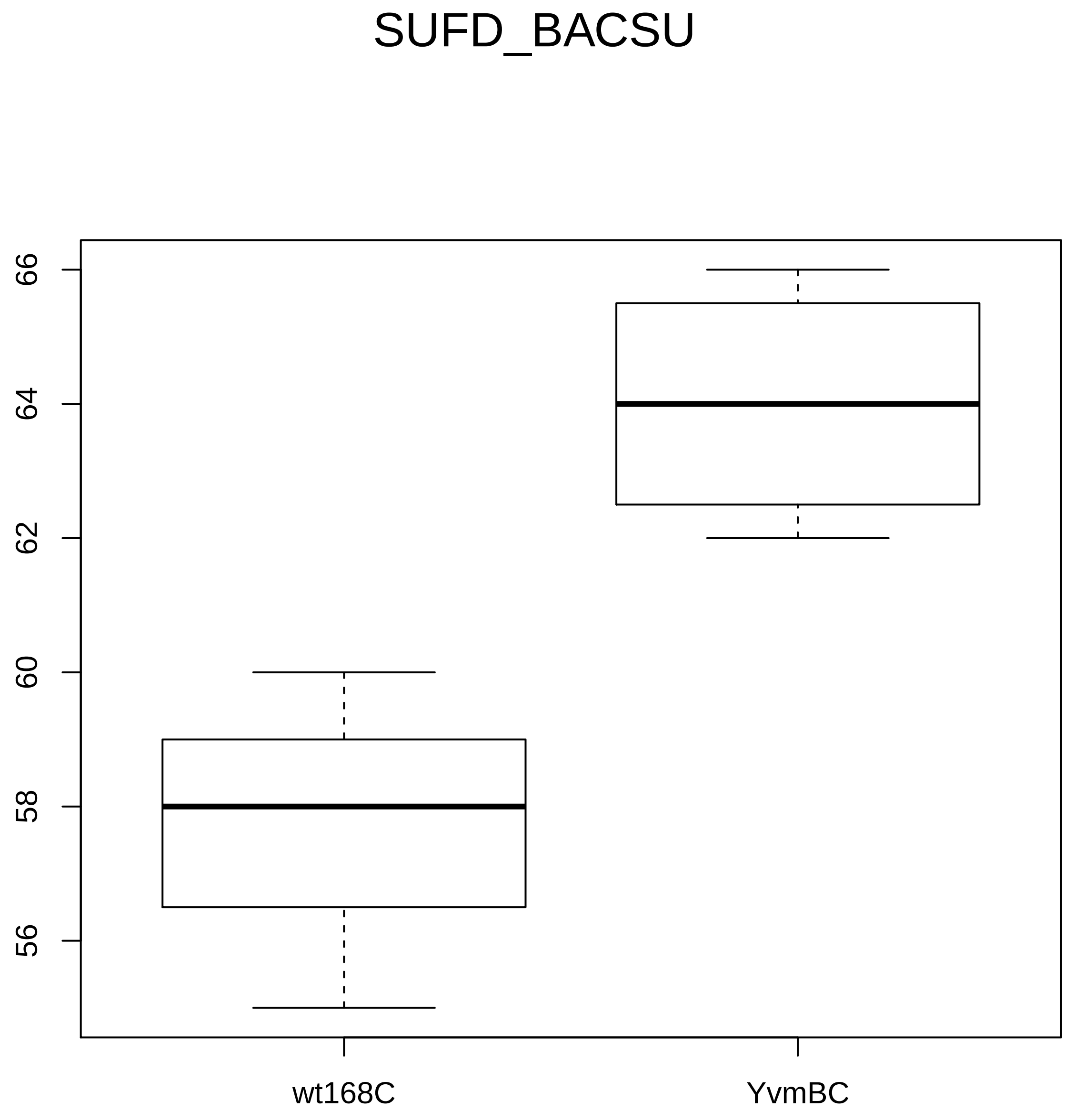

SUFD_BACSU
66
64
62
60
58
56
wt168C
YvmBC

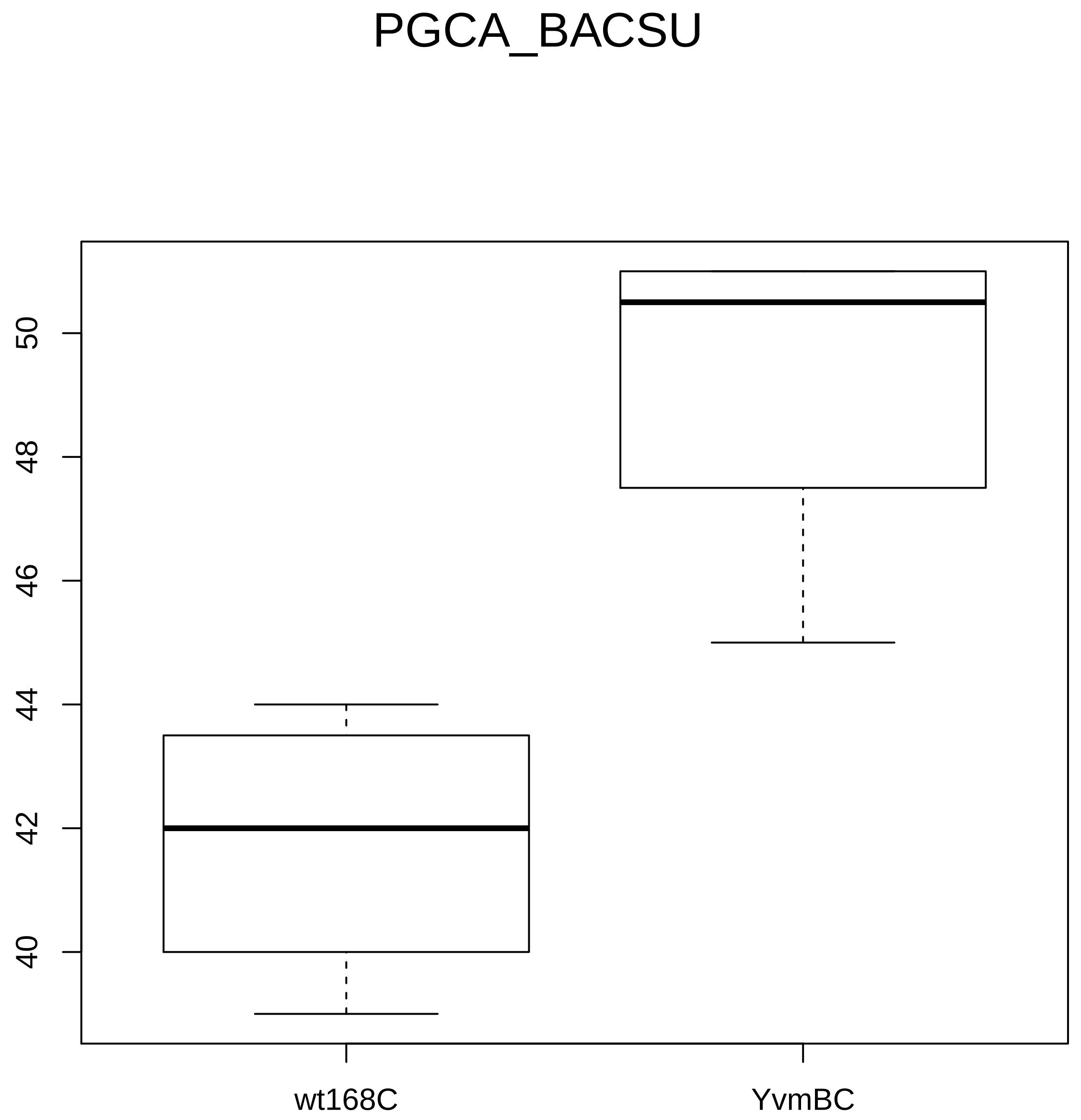

PGCA_BACSU
40
42
44
46
48
50
wt168C
YvmBC

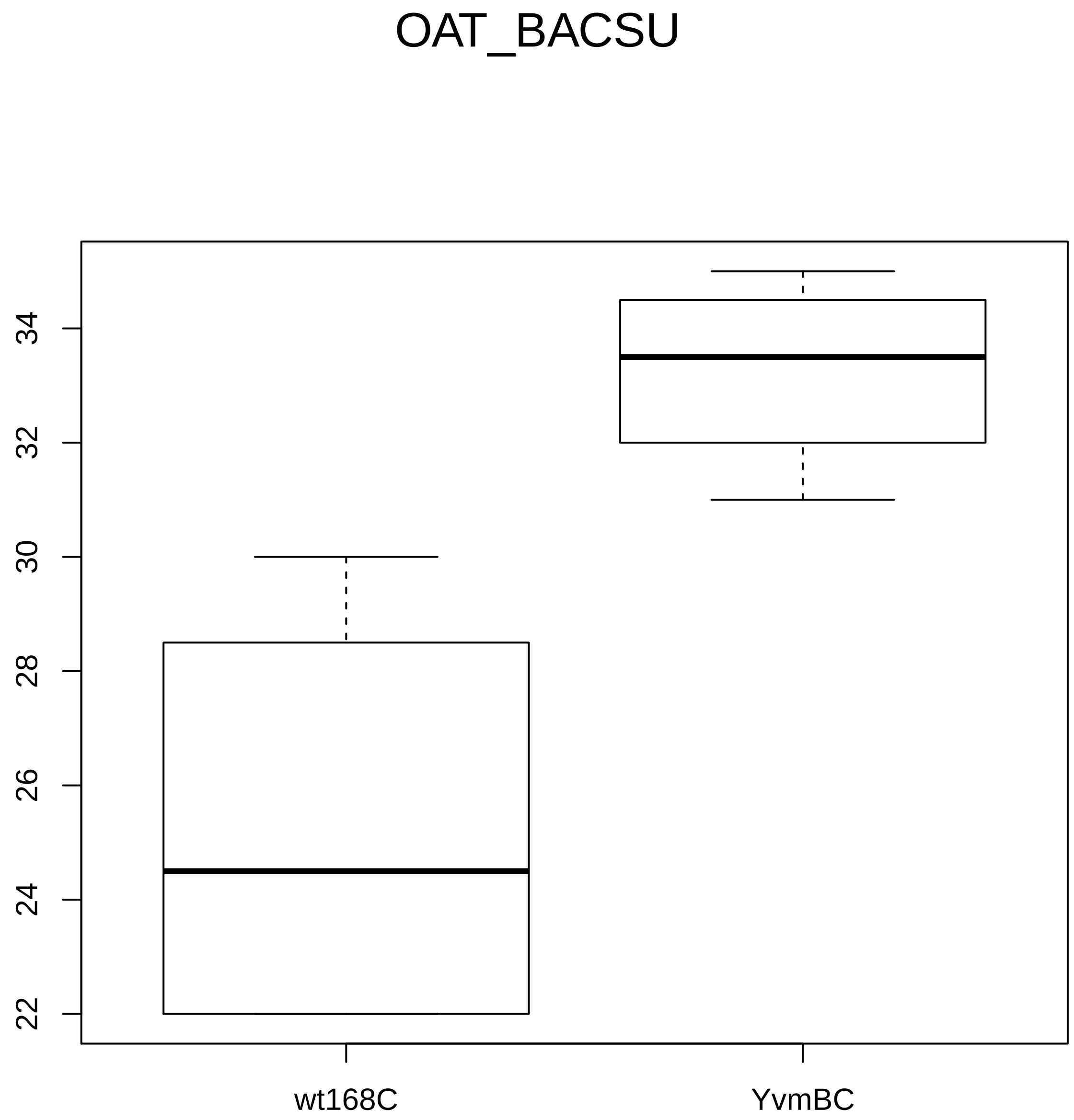
OAT_BACSU
22
24
26
28
30
32
34
wt168C
YvmBC

FENR2_BACSU

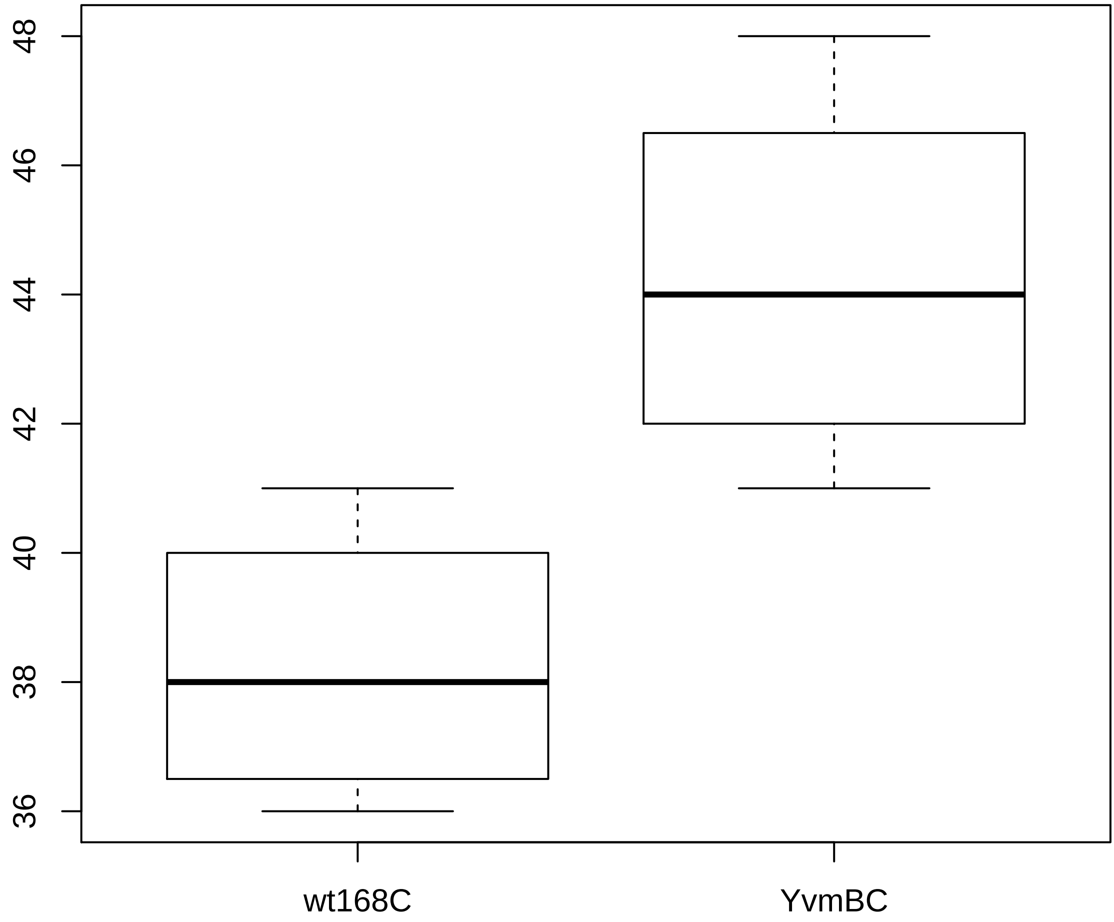

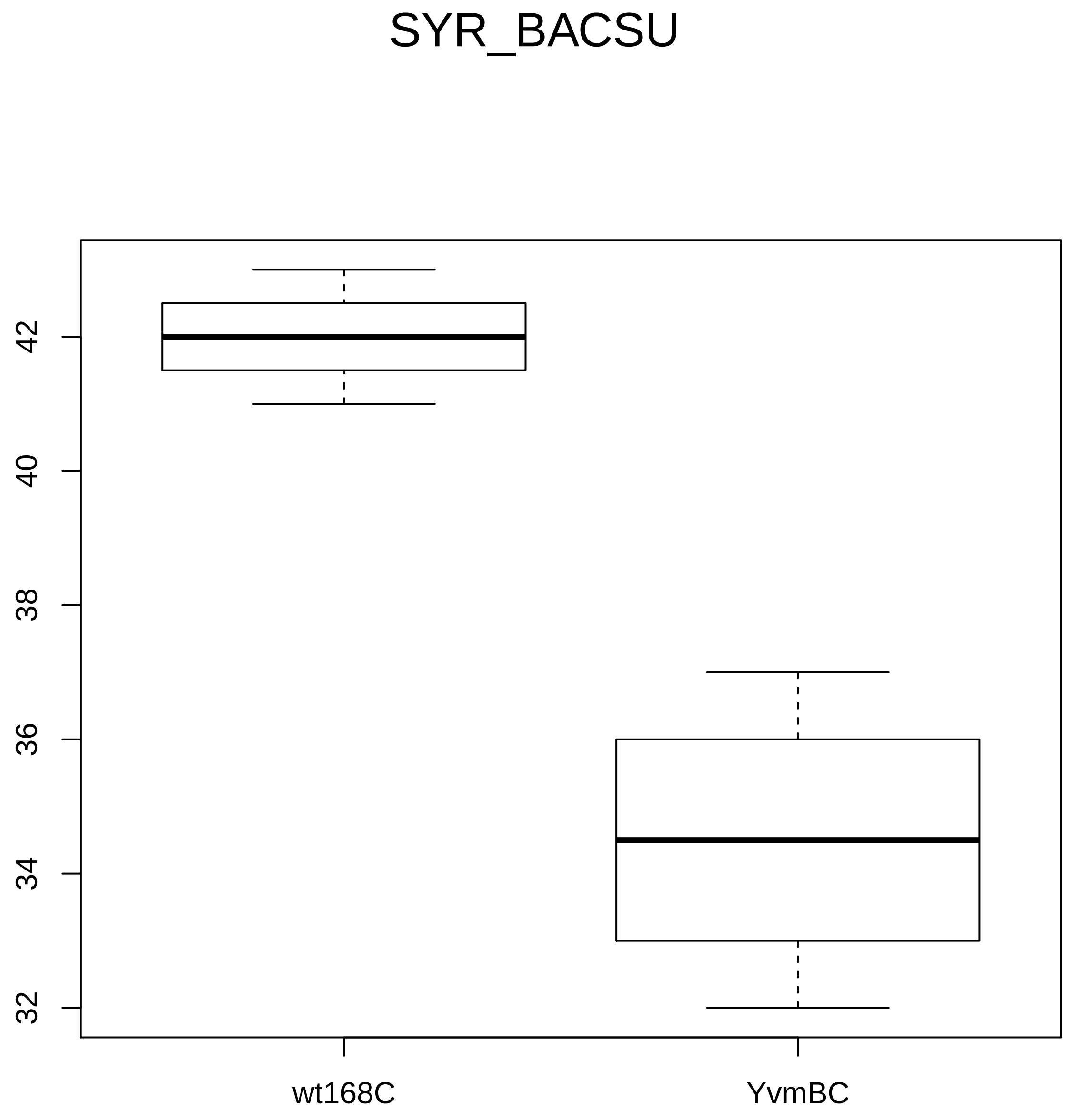

SYR_BACSU
42
40
38
36
34
32
wt168C
YvmBC

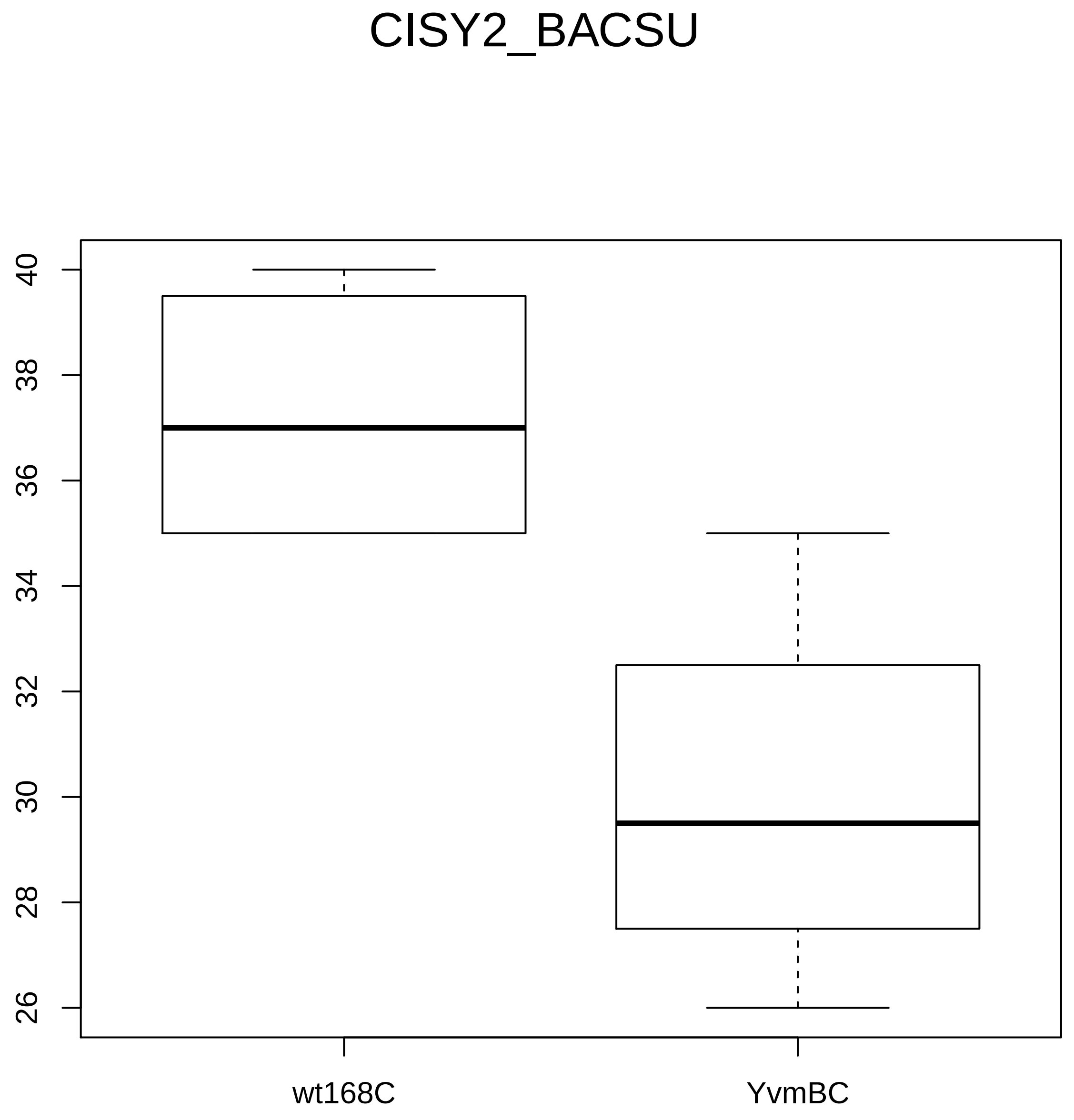
CISY2_BACSU
40
38
36
34
32
30
28
26
wt168C
YvmBC

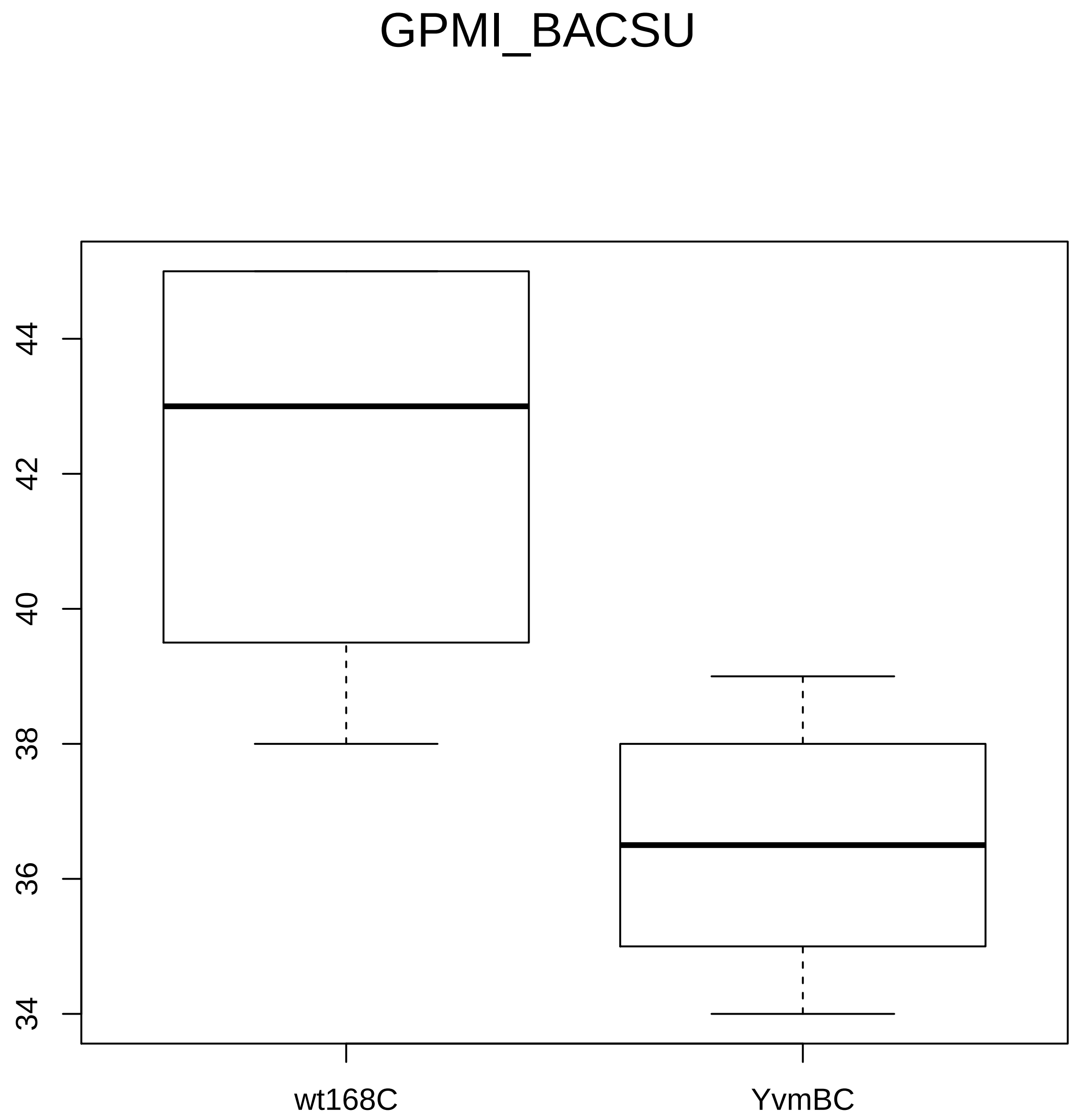
GPMI_BACSU
44
42
40
38
36
34
wt168C
YvmBC

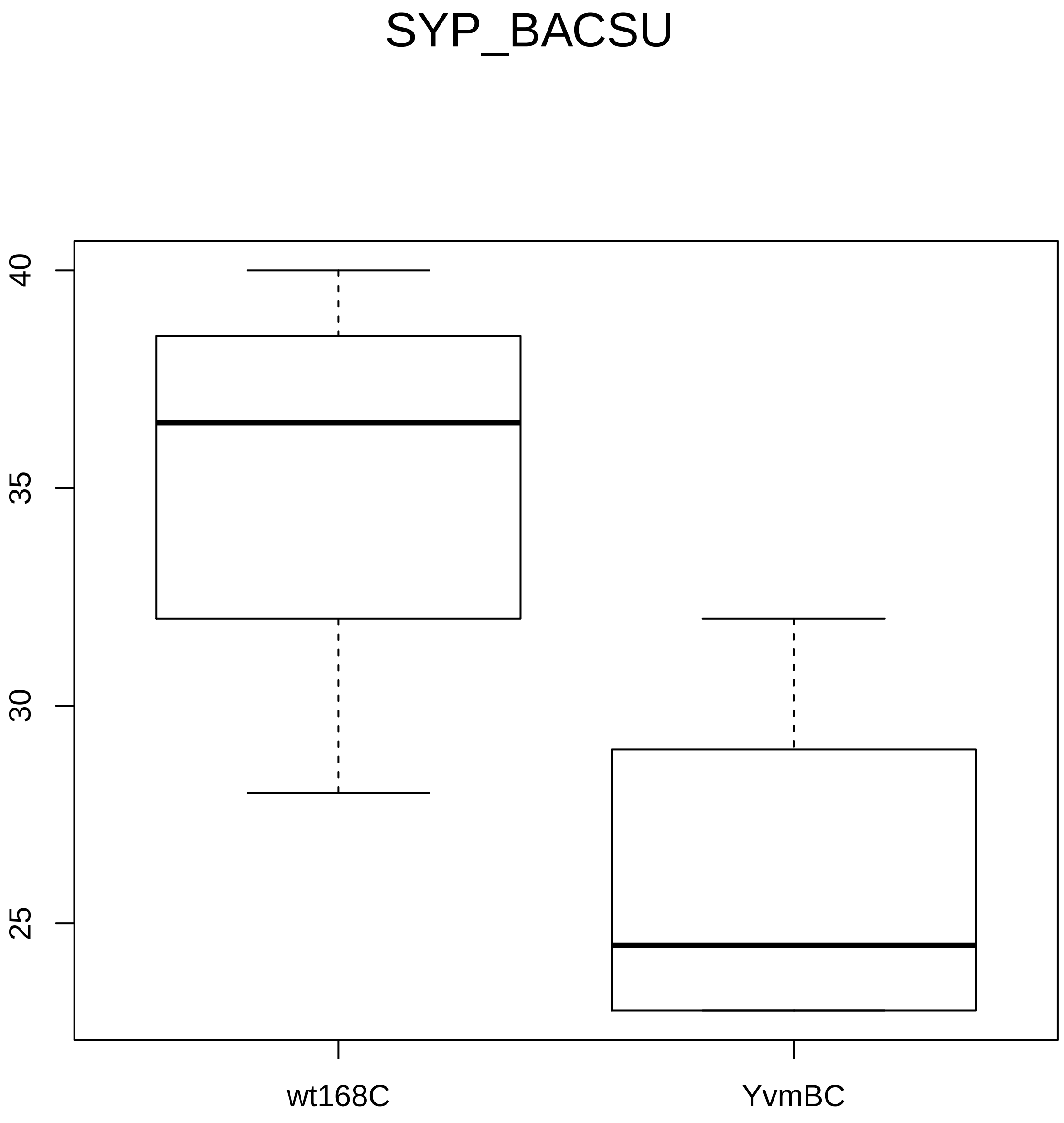

SYP_BACSU
40
35
30
25
wt168C
YvmBC

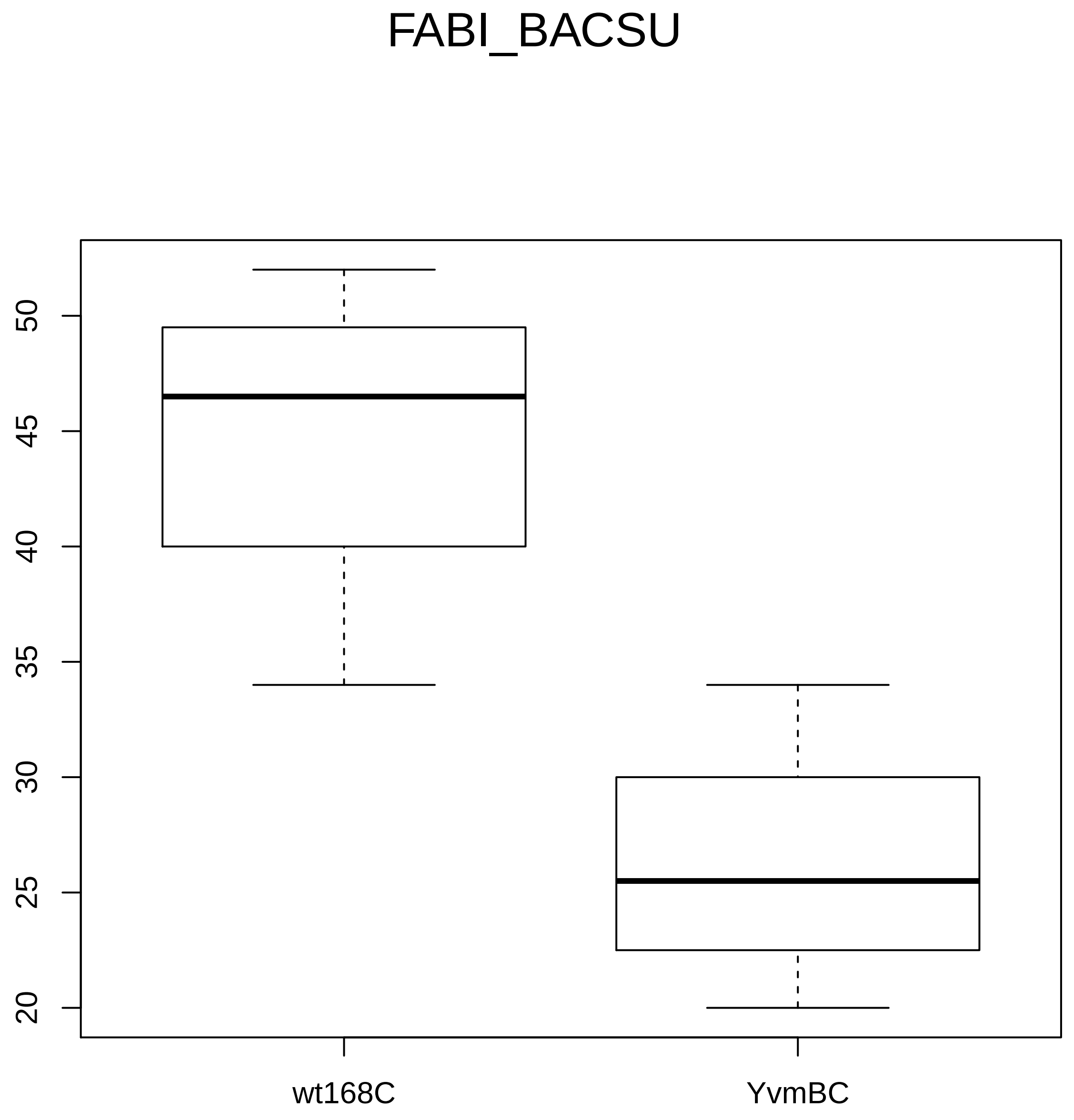

FABI_BACSU
20
25
30
35
40
45
50
wt168C
YvmBC

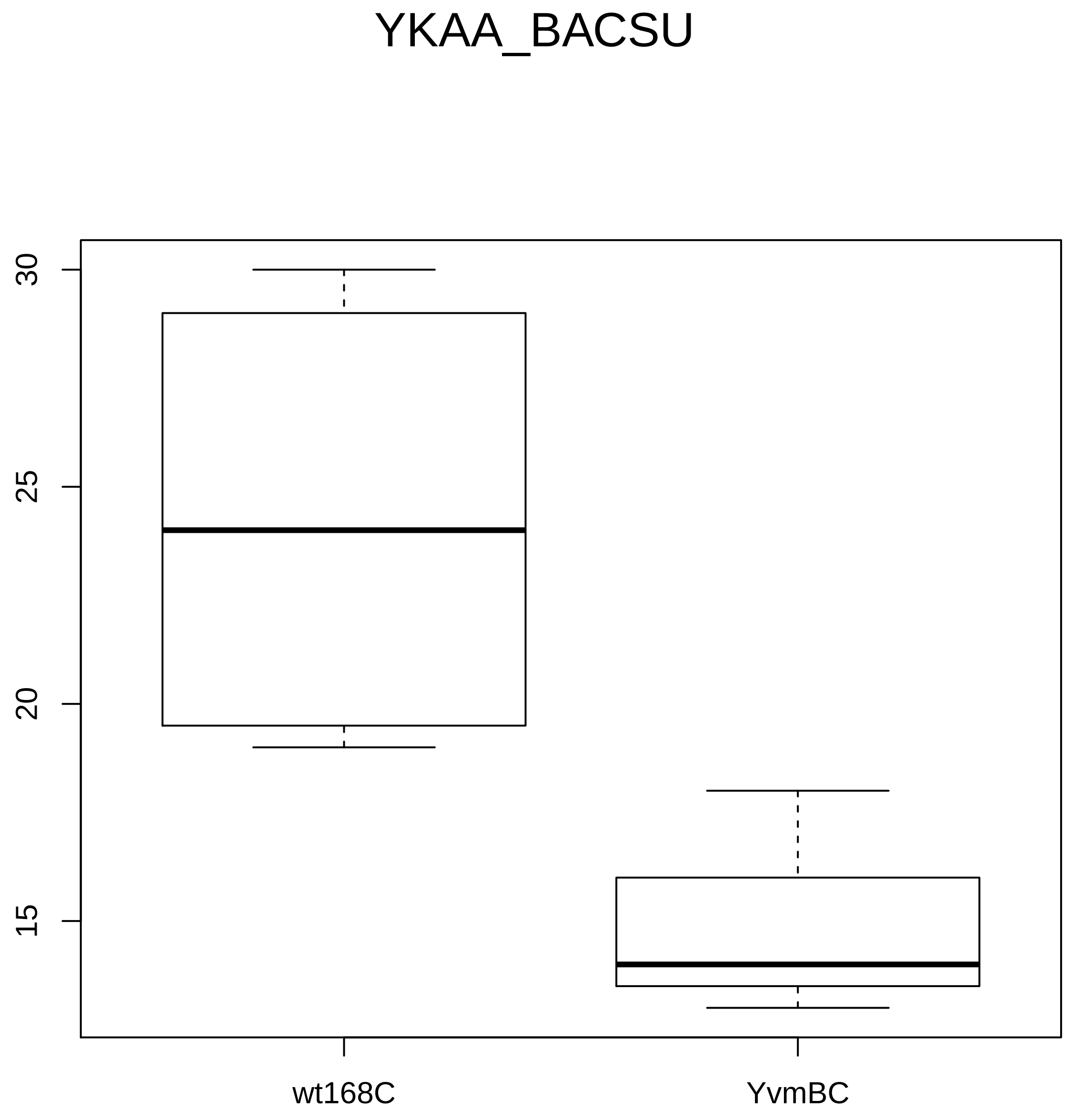
YKAA_BACSU
30
25
20
15
wt168C
YvmBC

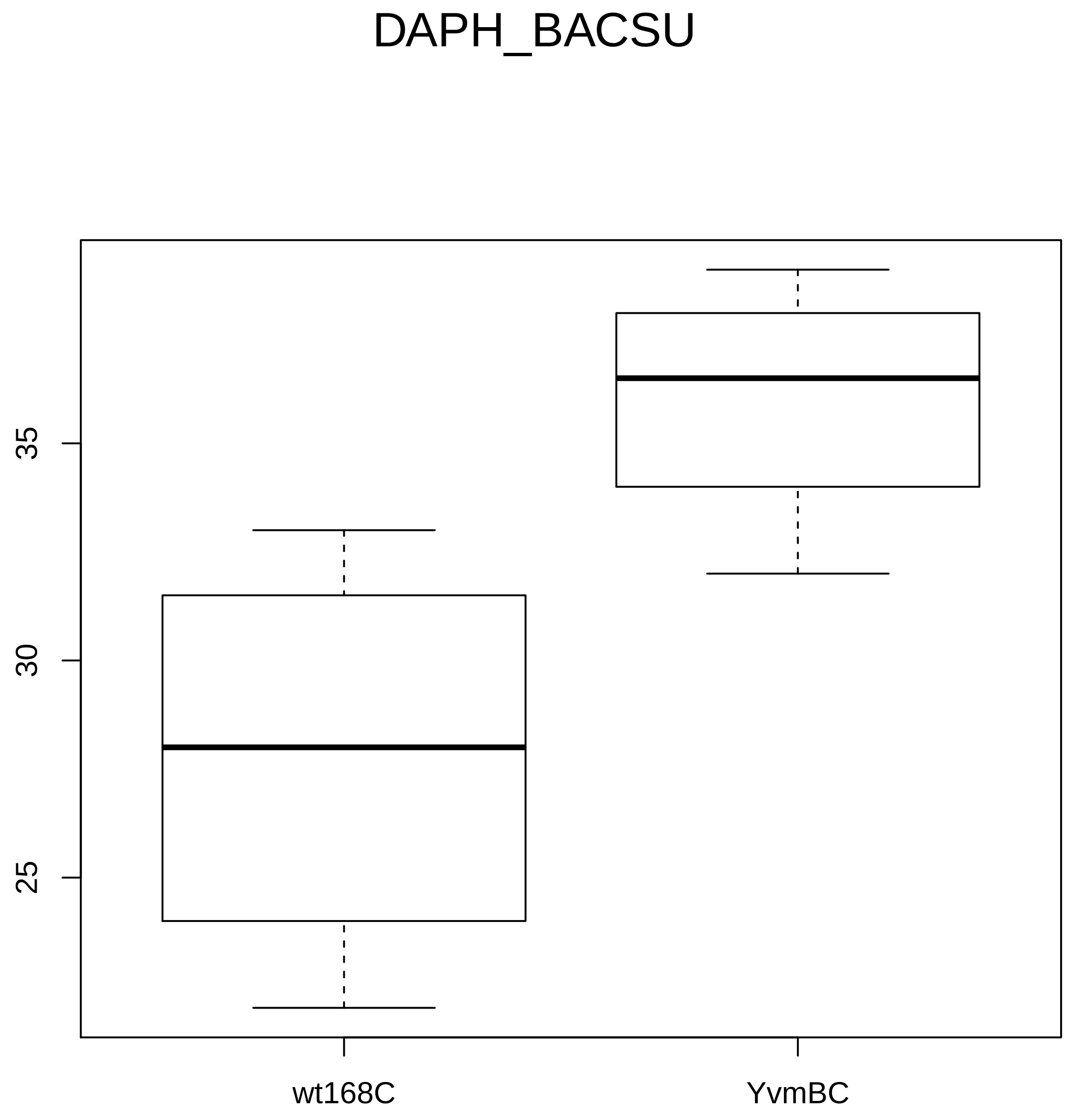

DAPH_BACSU
25
30
35
wt168C
YvmBC

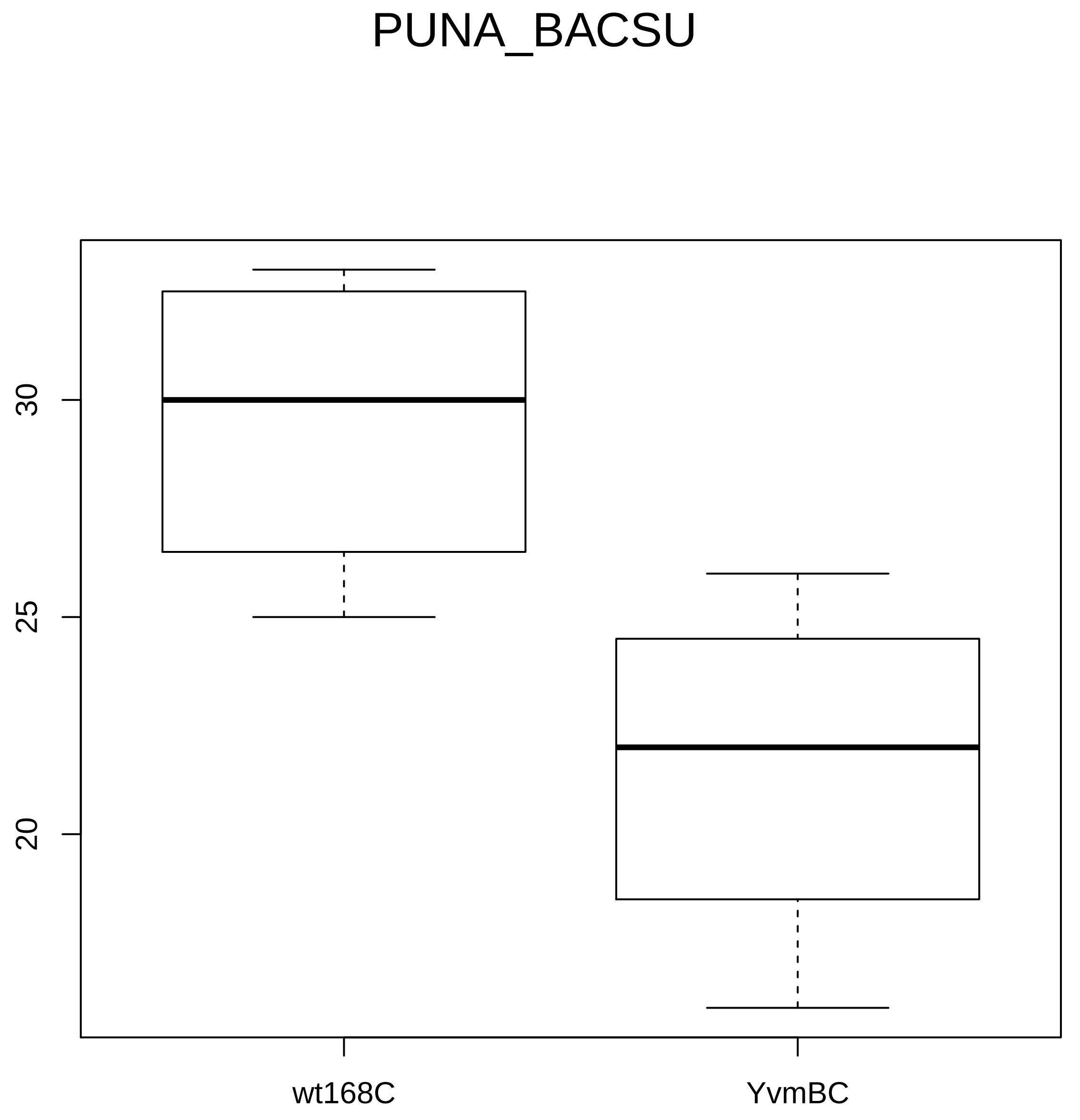
PUNA_BACSU
30
25
20
wt168C
YvmBC

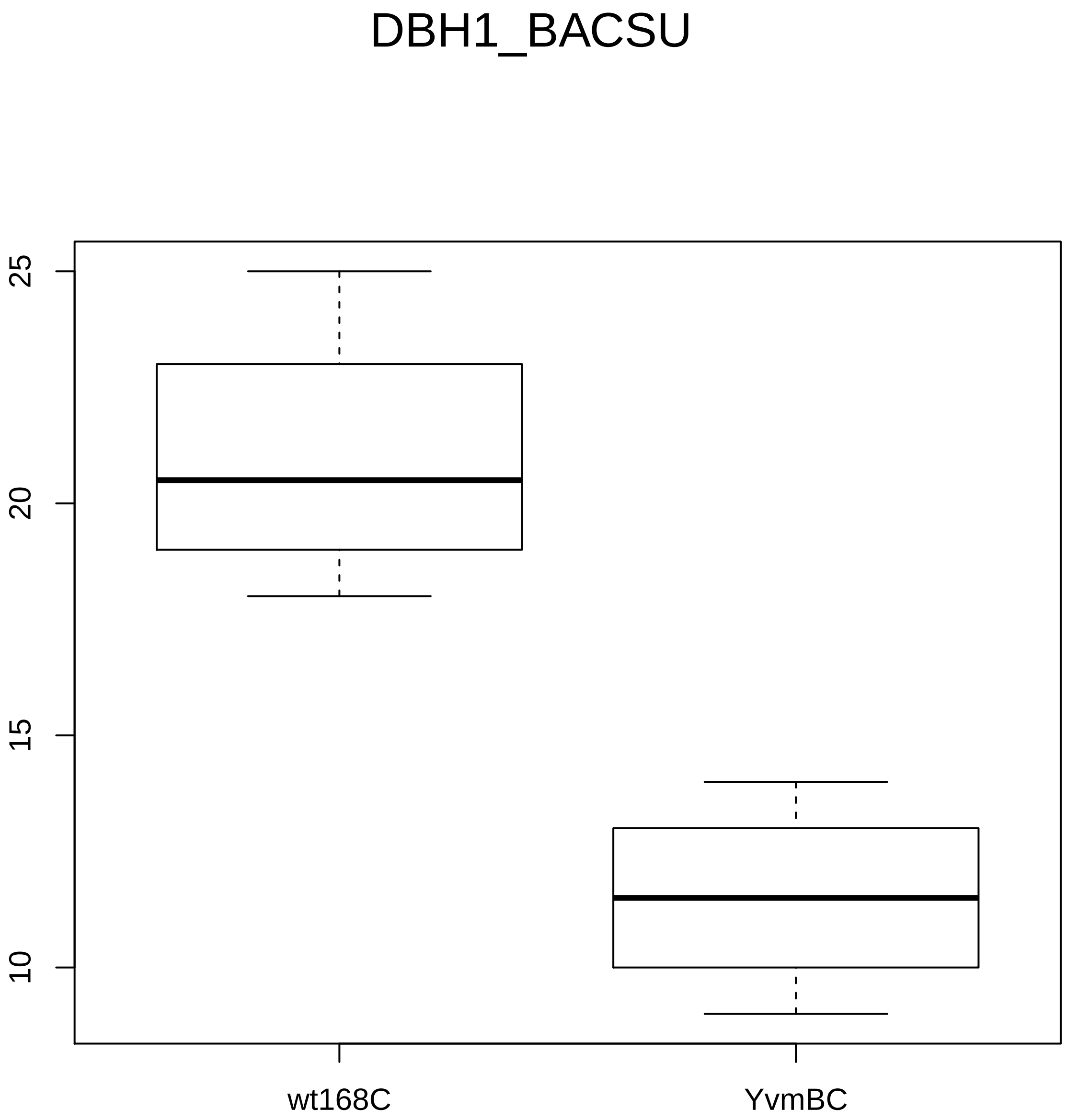
DBH1_BACSU
25
20
15
10
wt168C
YvmBC

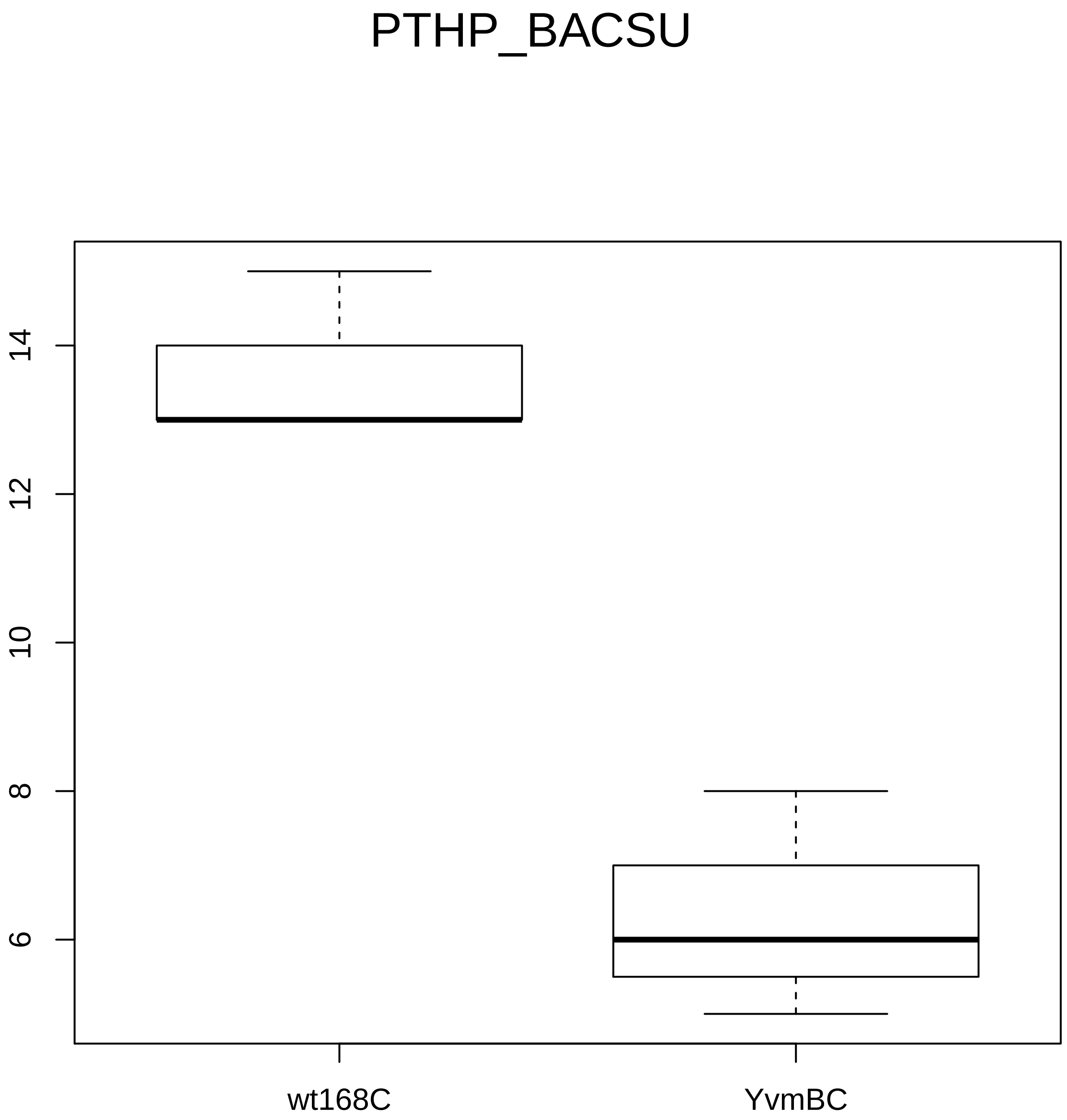
PTHP_BACSU
14
12
10
8
6
wt168C
YvmBC

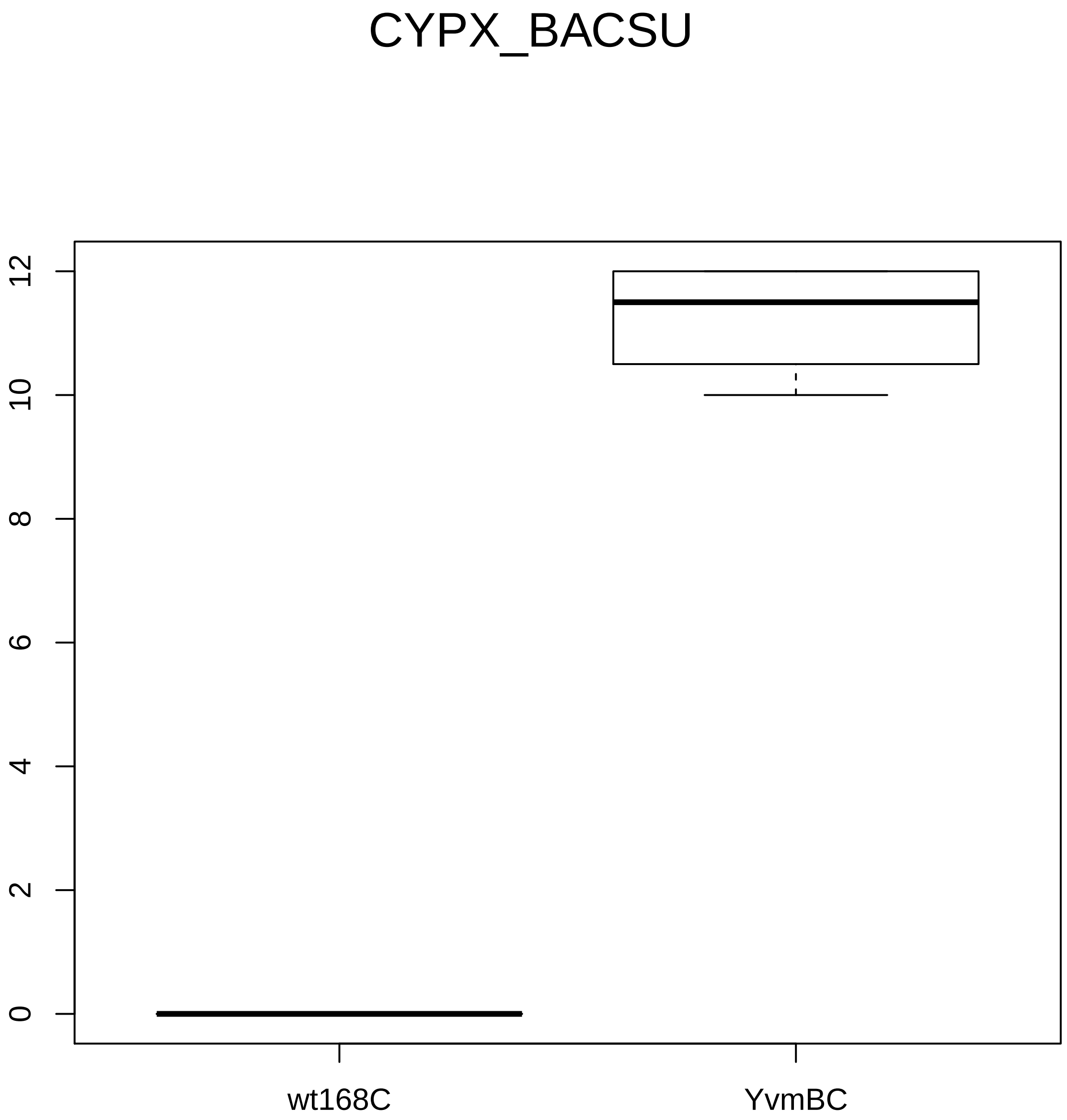
CYPX_BACSU
0
2
4
6
8
10
12
wt168C
YvmBC

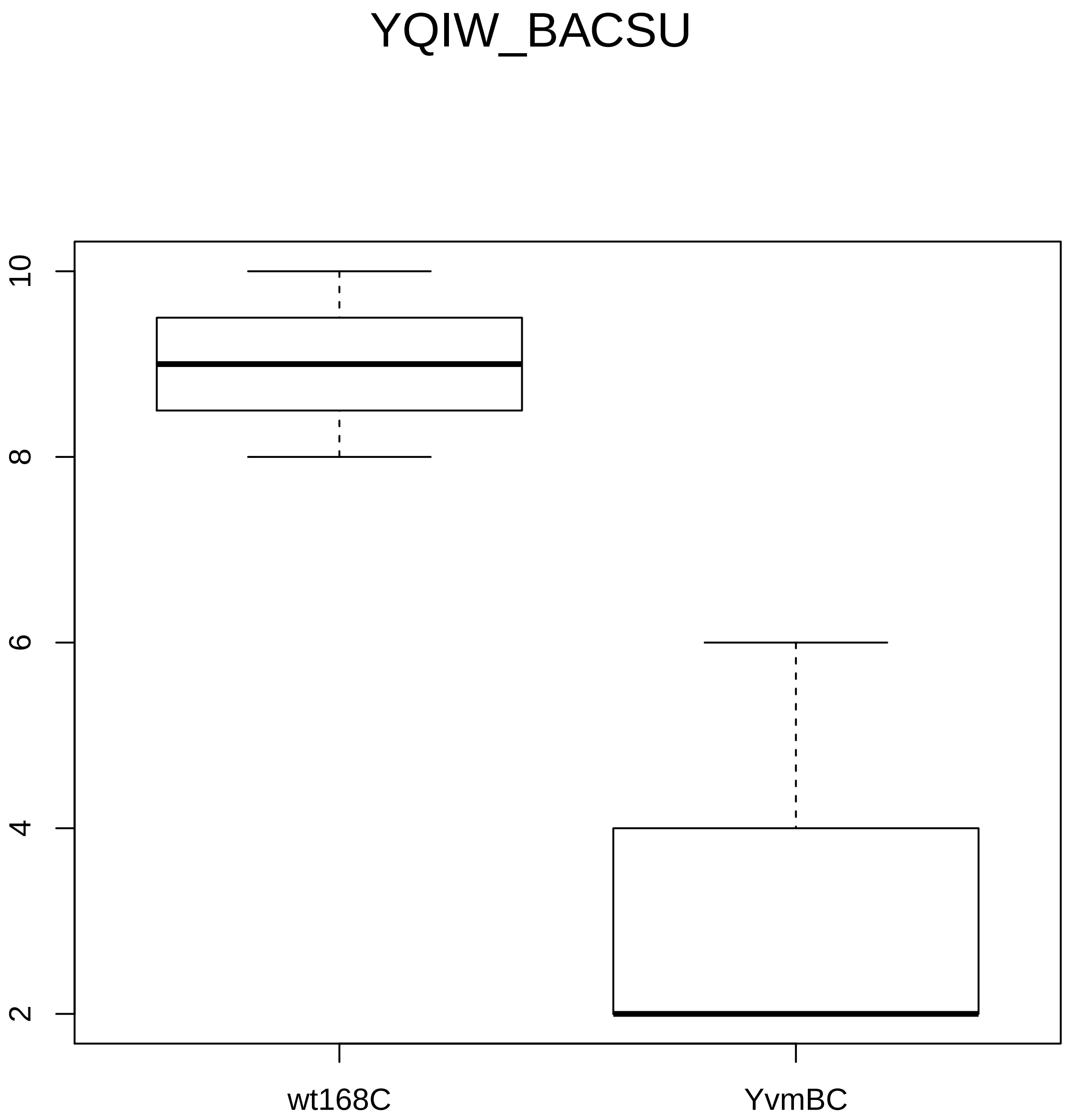
YQIW_BACSU
10
8
6
4
2
wt168C
YvmBC
