# Supplementary material for: The MarR-like protein PchR (YvmB) regulates expression of genes involved in pulcherriminic acid biosynthesis and in the initiation of sporulation in Bacillus subtilis
Source: BMC Microbiol. 2016 Aug 20;16:190. doi: 10.1186/s12866-016-0807-3 (PMC4992311; doi:10.1186/s12866-016-0807-3)
Supplement: Additional file 10: Figure S7. — Proteins differing significantly in ΔyvmB cells from proteome analysis of the membrane fraction. A Kruskal-Wallis one-way analysis of variance (ANOVA) was done on the whole proteomic data. (PDF 13 kb) [file 12866_2016_807_MOESM10_ESM.pdf]

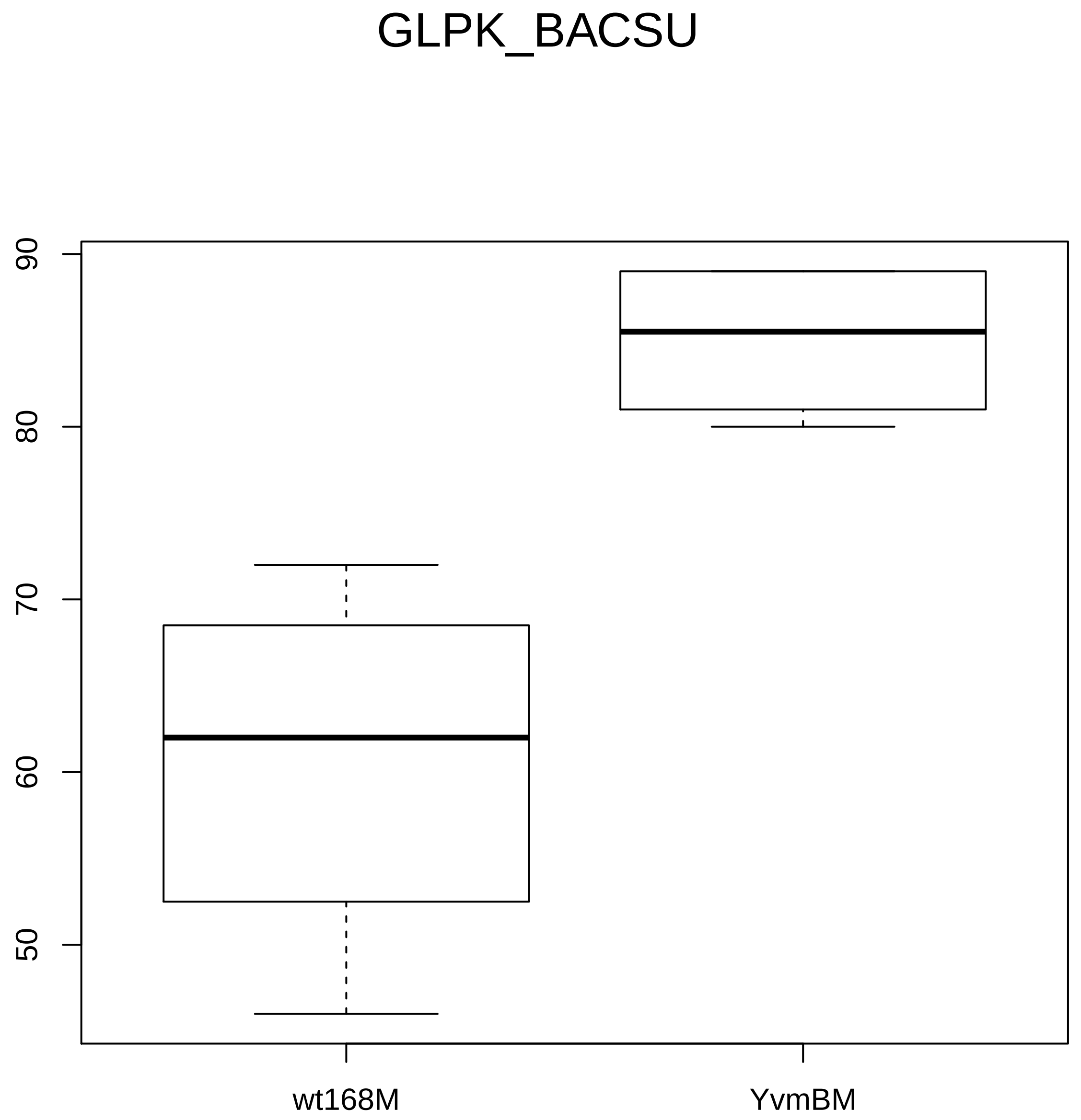
GLPK_BACSU
90
80
70
60
50
wt168M
YvmBM

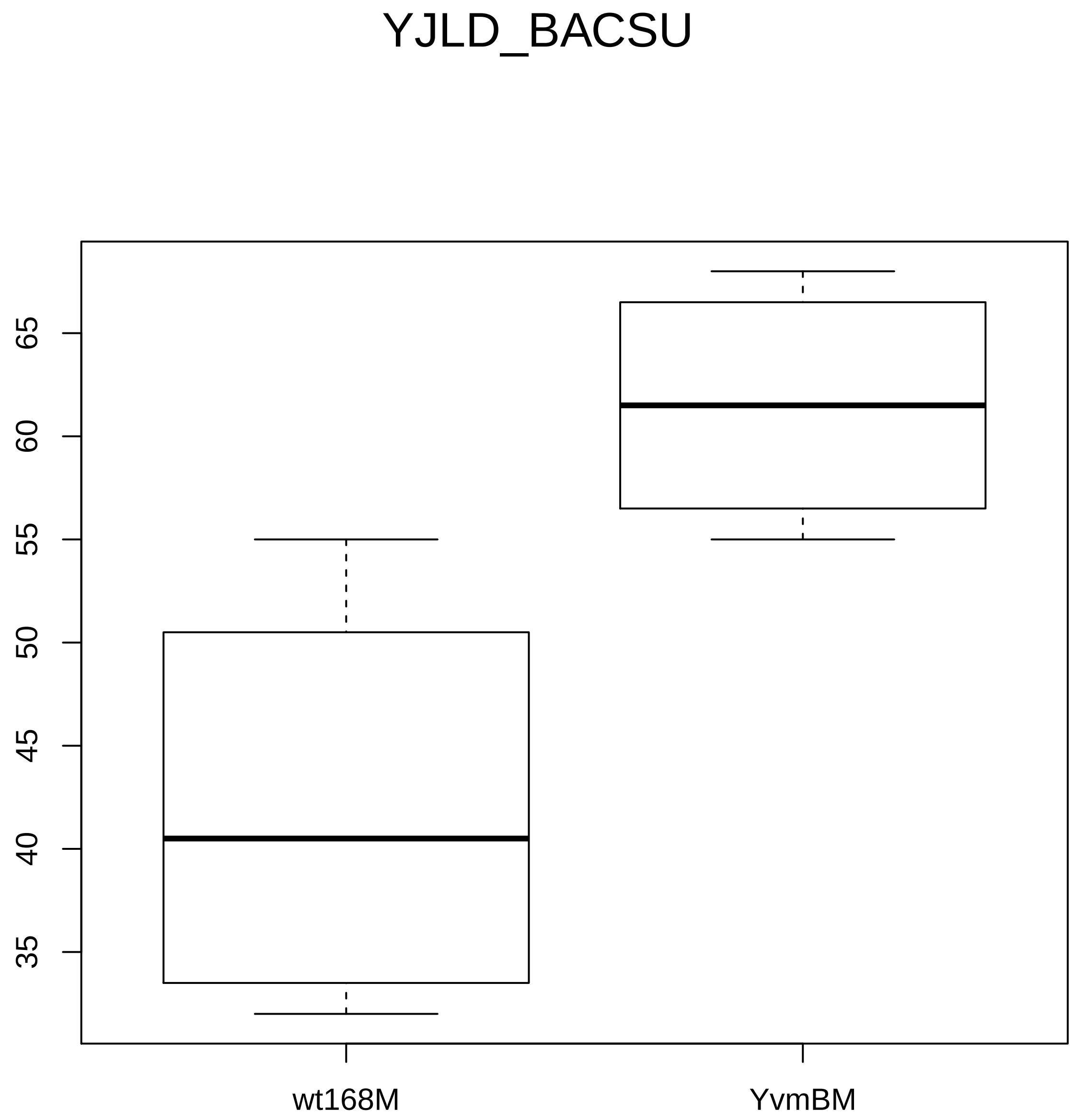
YJLD_BACSU
35
40
45
50
55
60
65
wt168M
YvmBM

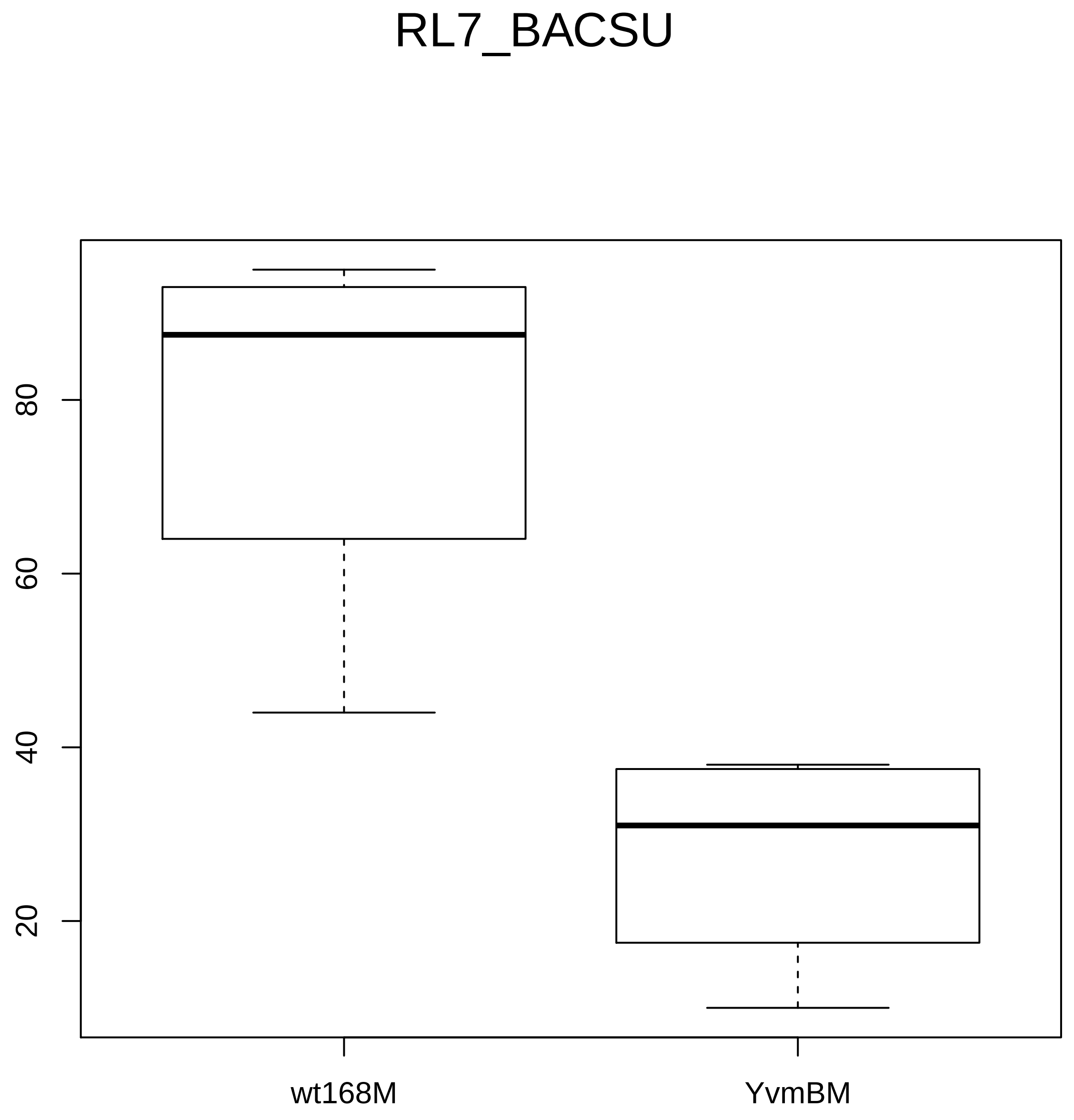
RL7_BACSU
80
60
40
20
wt168M
YvmBM

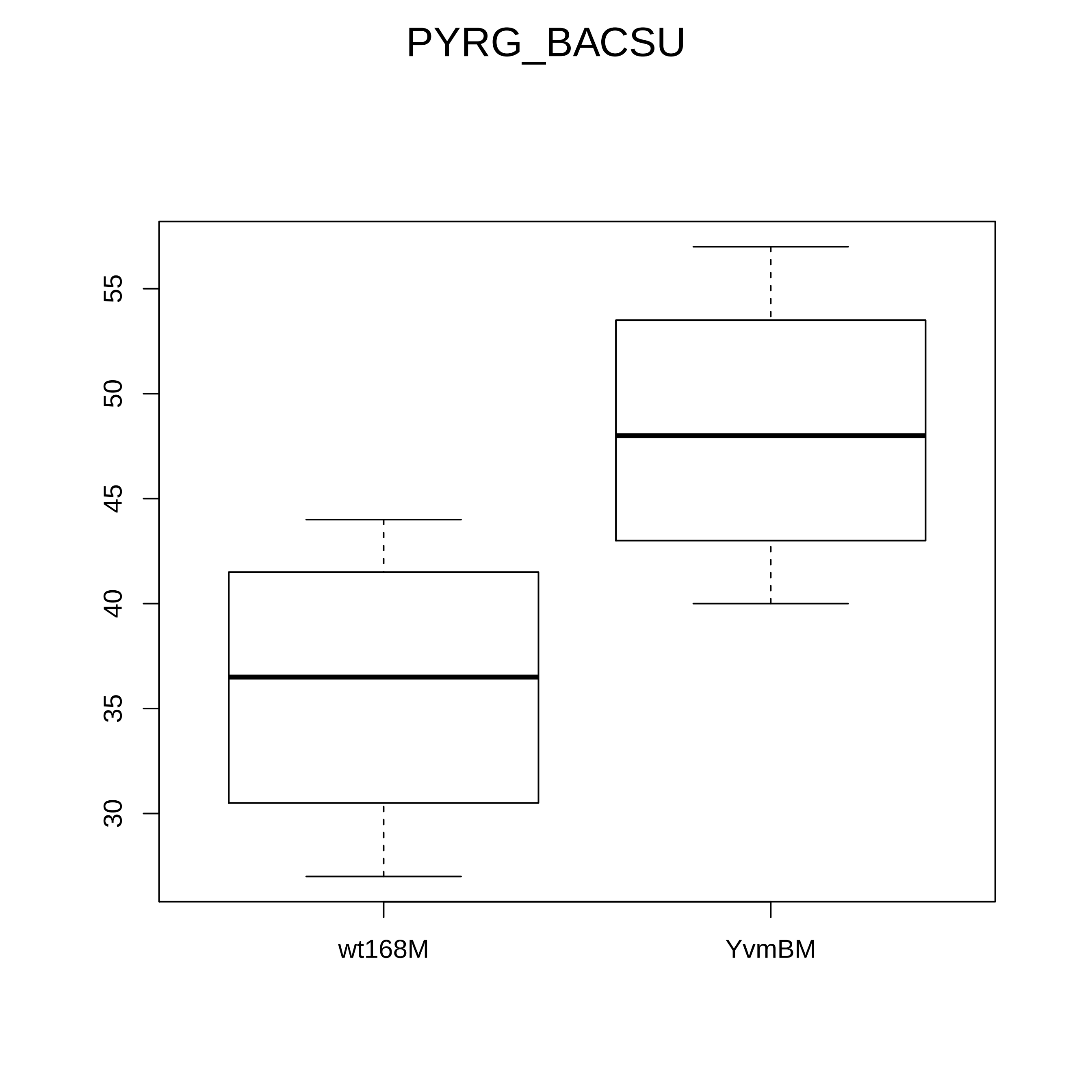
PYRG_BACSU
55
50
45
40
35
30
wt168M
YvmBM

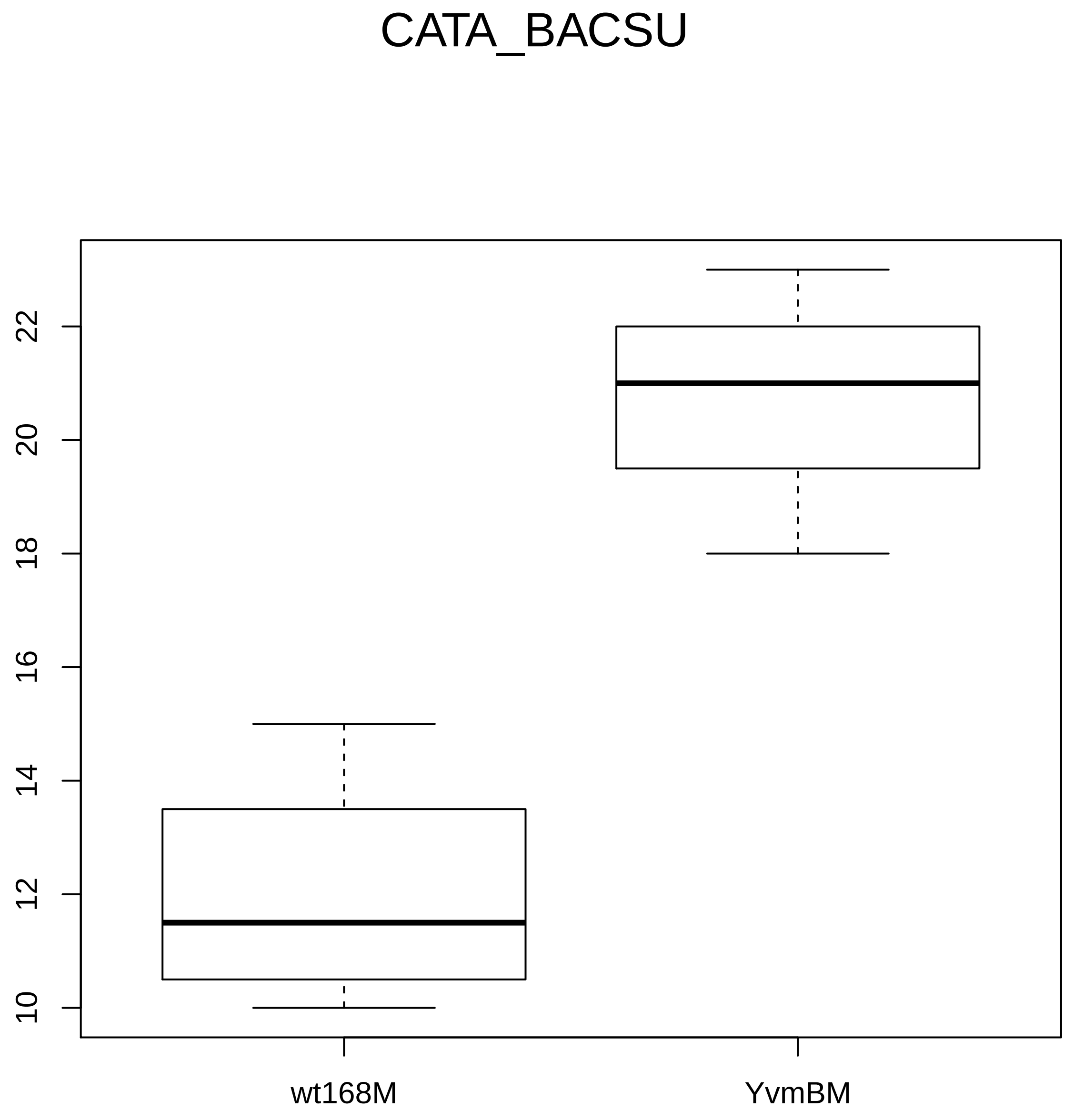

CATA_BACSU
10
12
14
16
18
20
22
wt168M
YvmBM

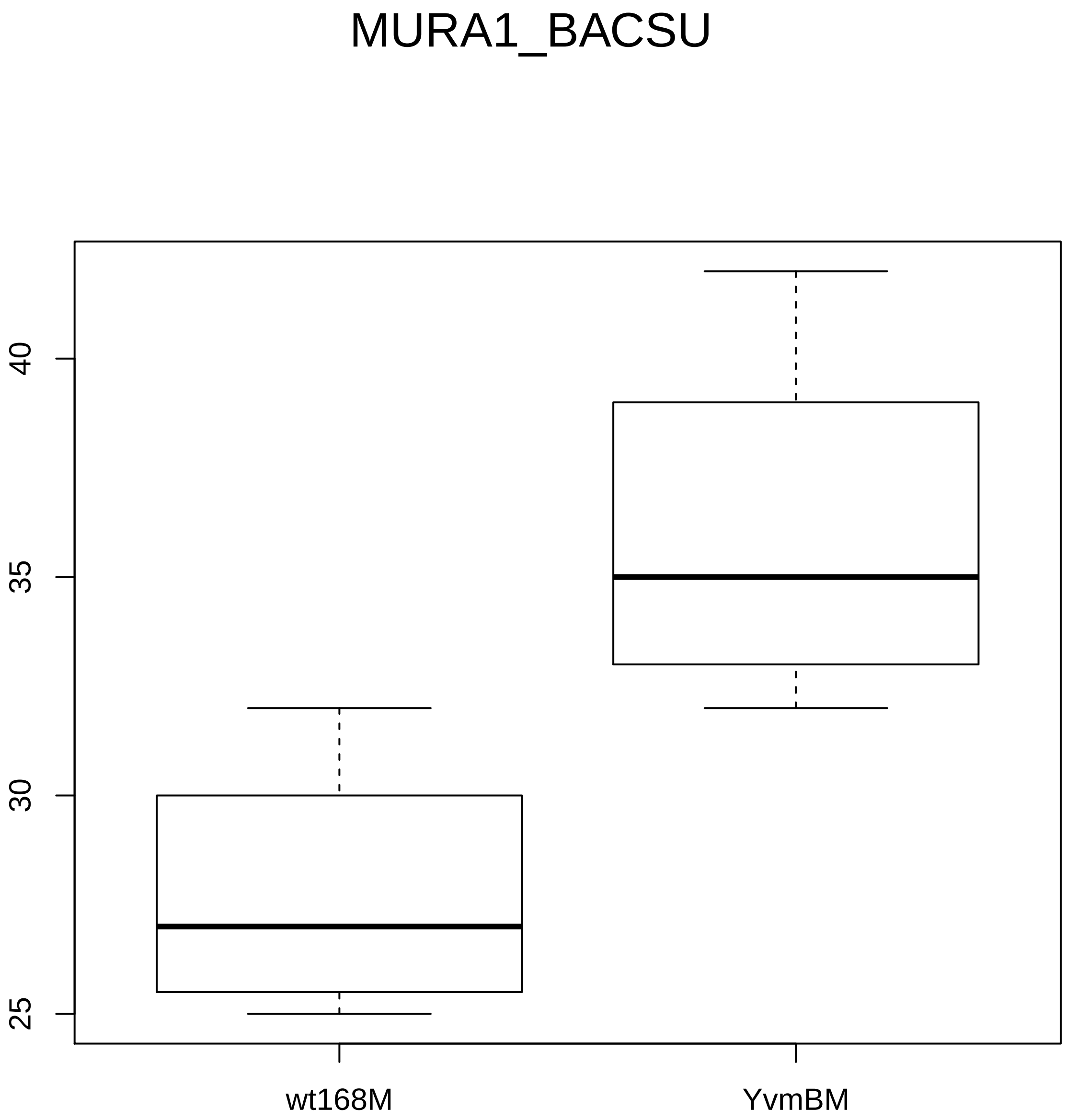
MURA1_BACSU
40
35
30
25
wt168M
YvmBM

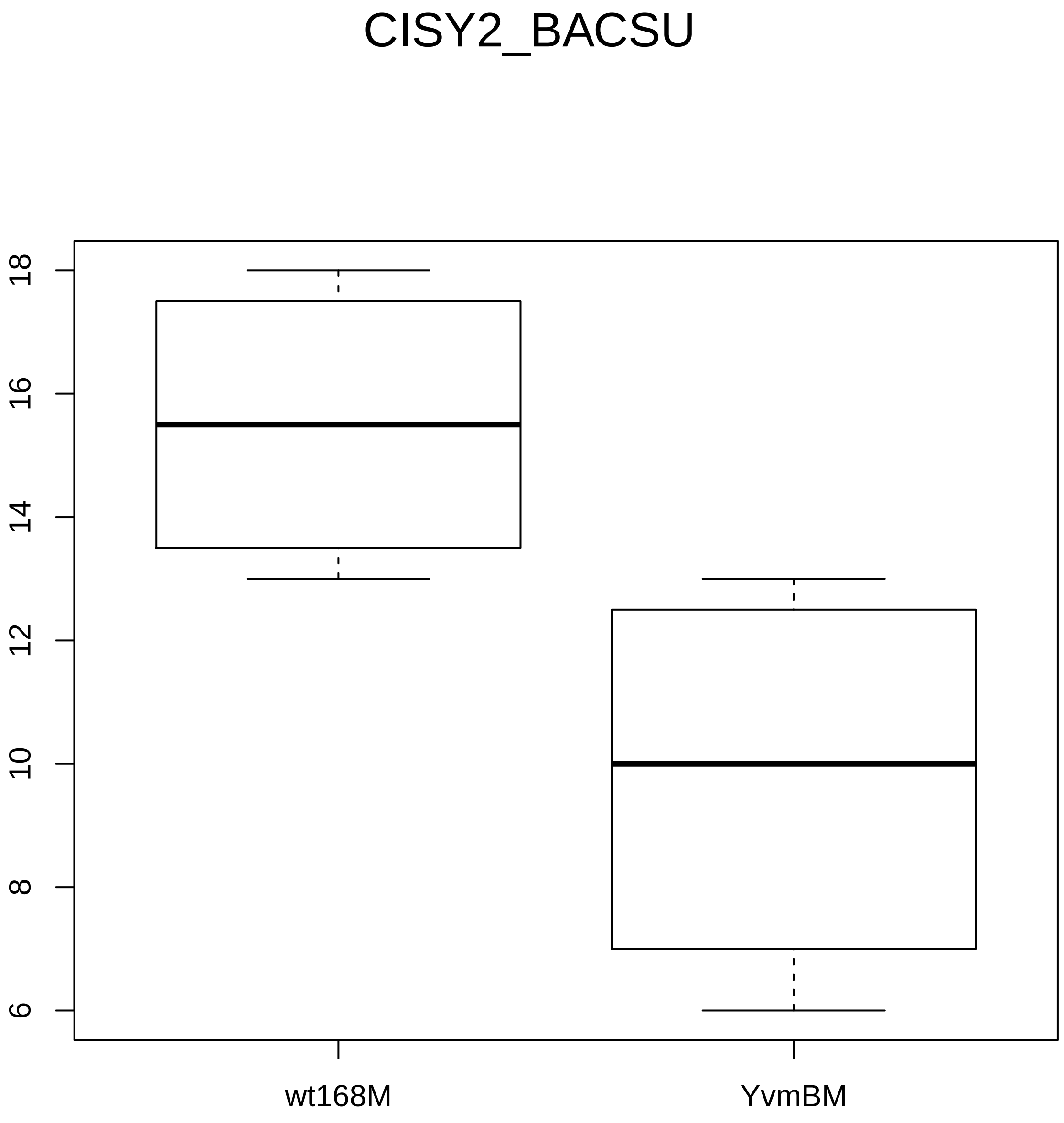
CISY2_BACSU
18
16
14
12
10
8
6
wt168M
YvmBM

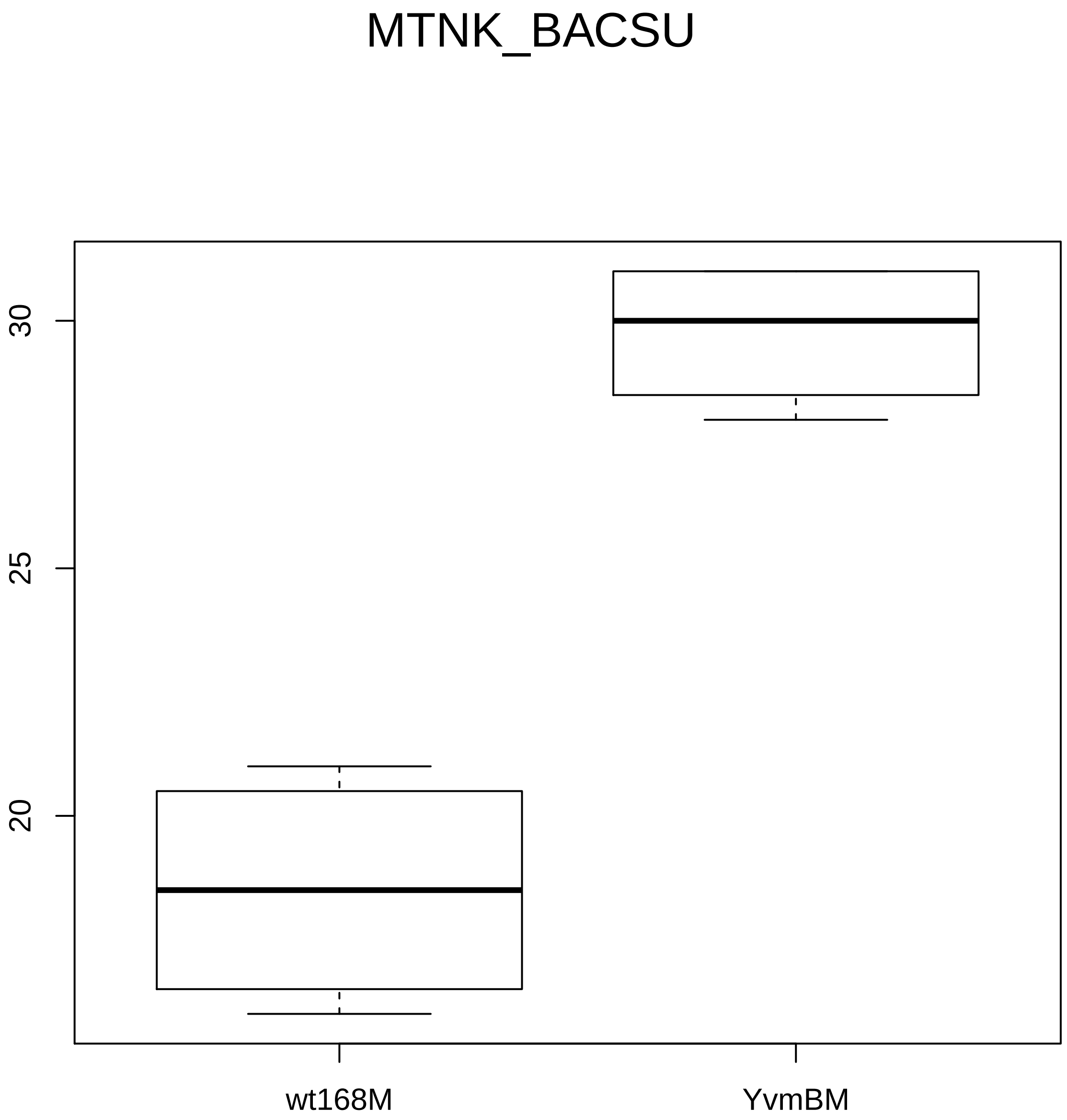
MTNK_BACSU
30
25
20
wt168M
YvmBM

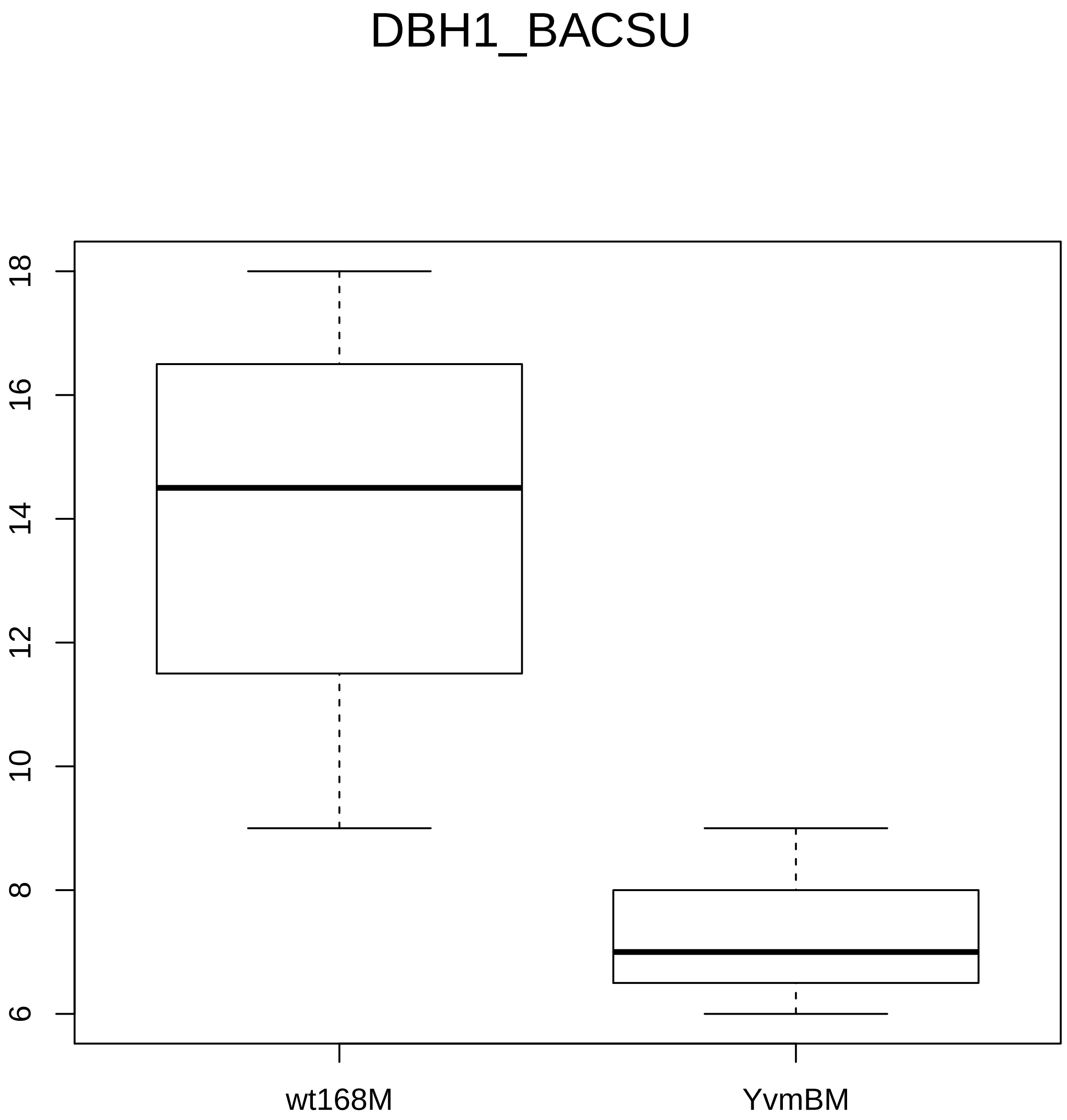
DBH1_BACSU
18
16
14
12
10
8
6
wt168M
YvmBM

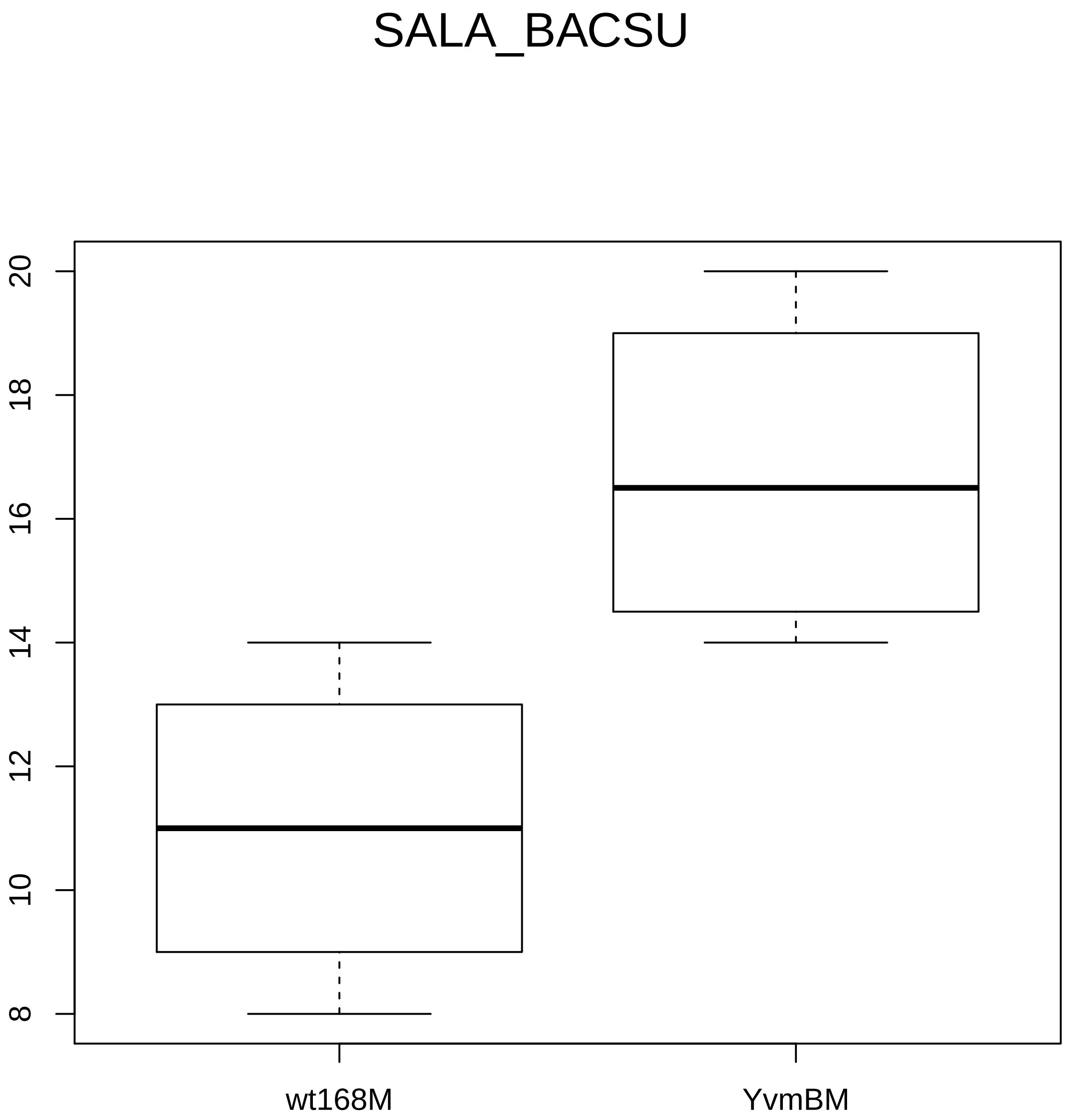

SALA_BACSU
20
18
16
14
12
10
8
wt168M
YvmBM

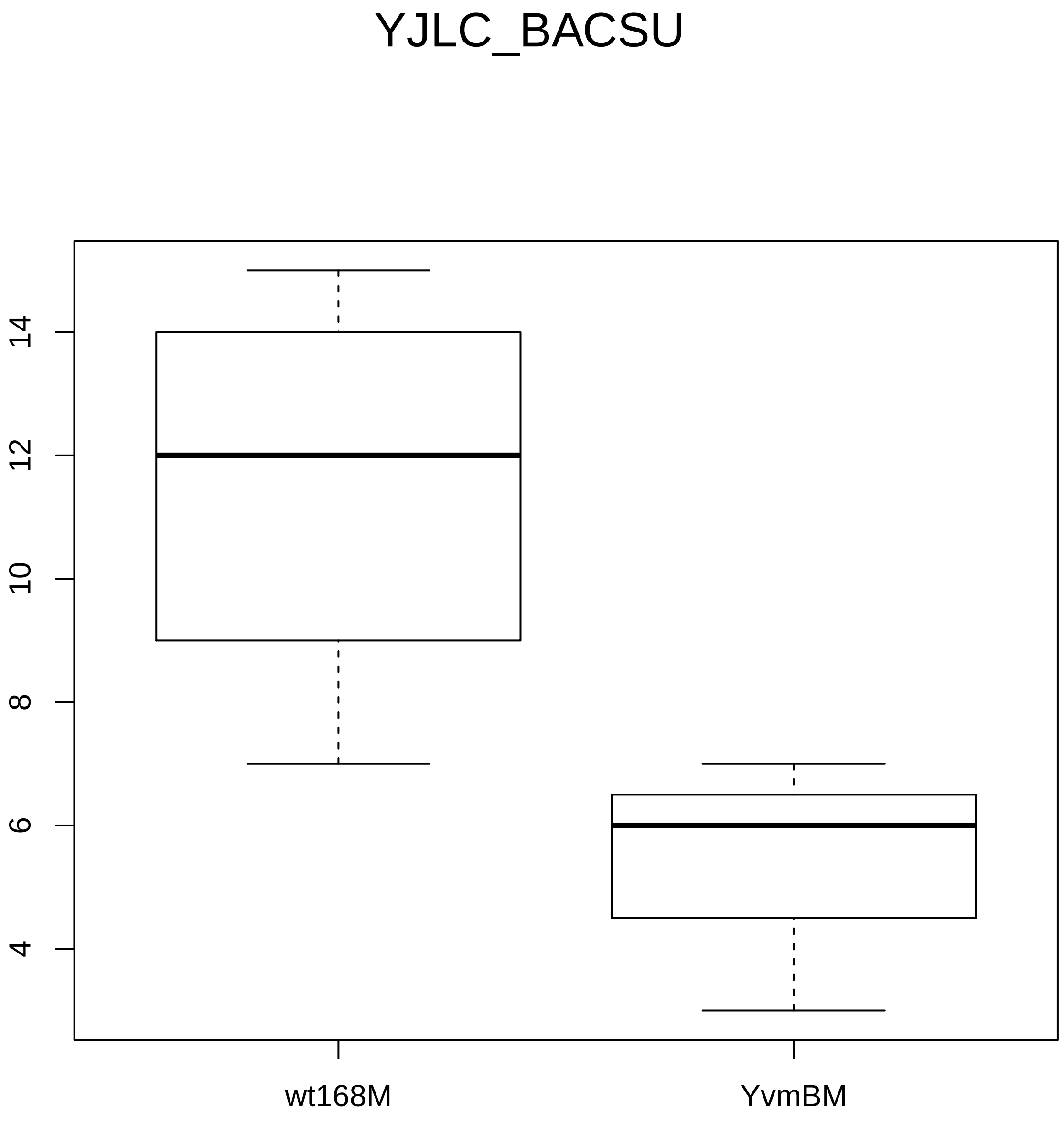
YJLC_BACSU
14
12
10
8
6
4
wt168M
YvmBM

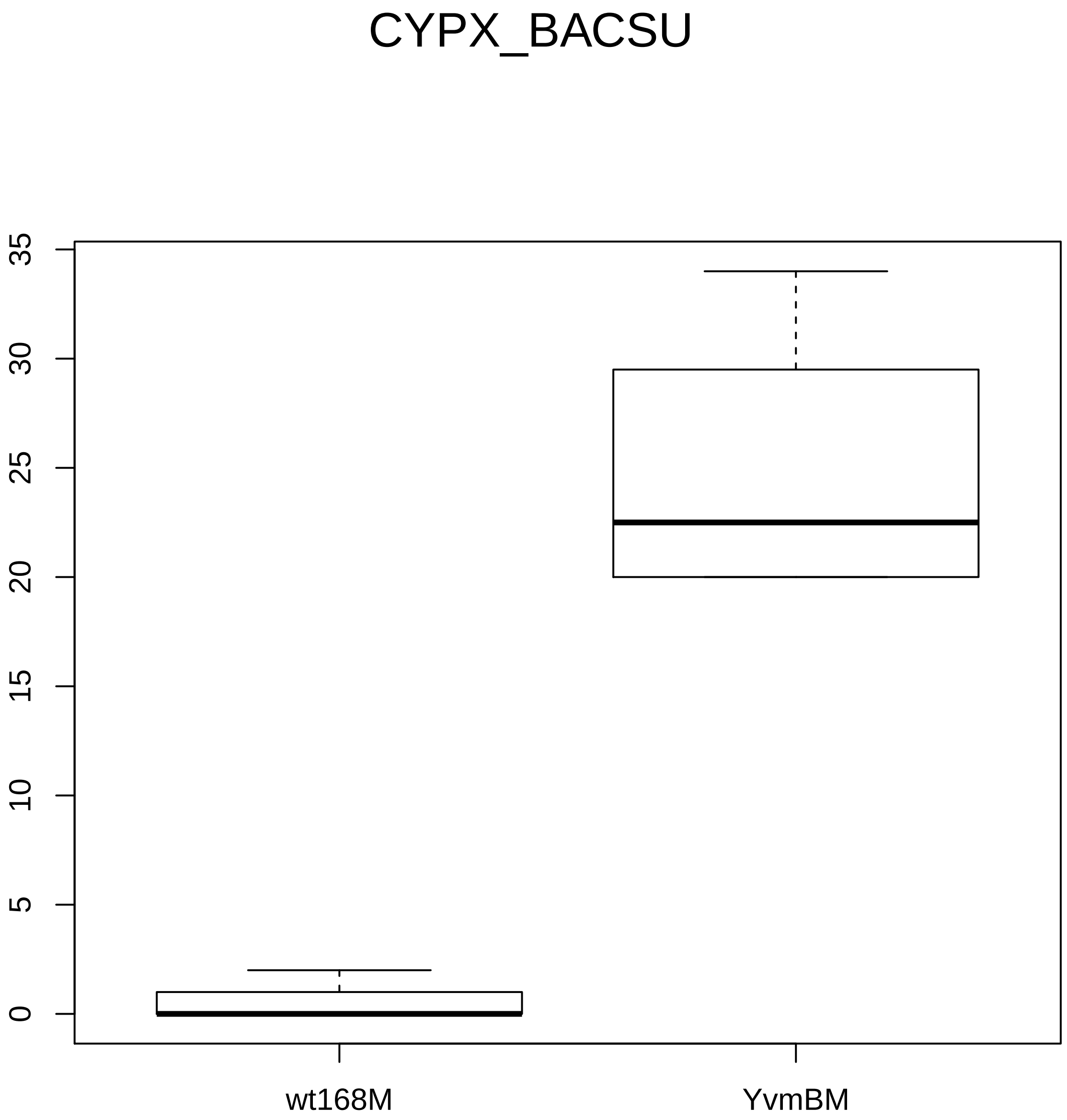
CYPX_BACSU
0
5
10
15
20
25
30
35
wt168M
YvmBM

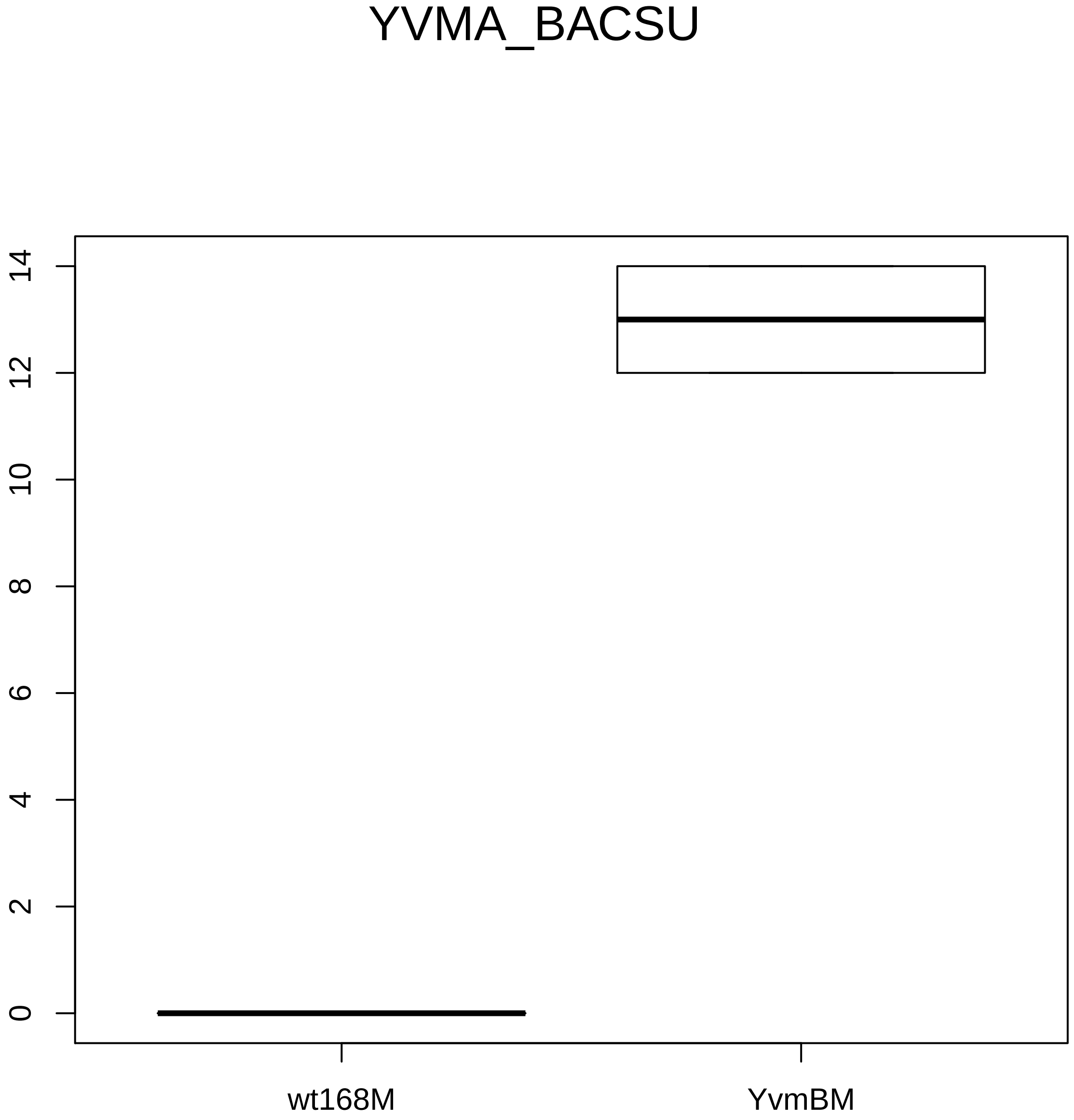
YVMA_BACSU
0
2
4
6
8
10
12
14
wt168M
YvmBM
